# Supplementary material for: Antimicrobial Activity and Chemical Constitution of the Crude, Phenolic-Rich Extracts of Hibiscus sabdariffa, Brassica oleracea and Beta vulgaris
Source: Molecules. 2019 Nov 24;24(23):4280. doi: 10.3390/molecules24234280 (PMC6930538; doi:10.3390/molecules24234280)

Supplementary Materials

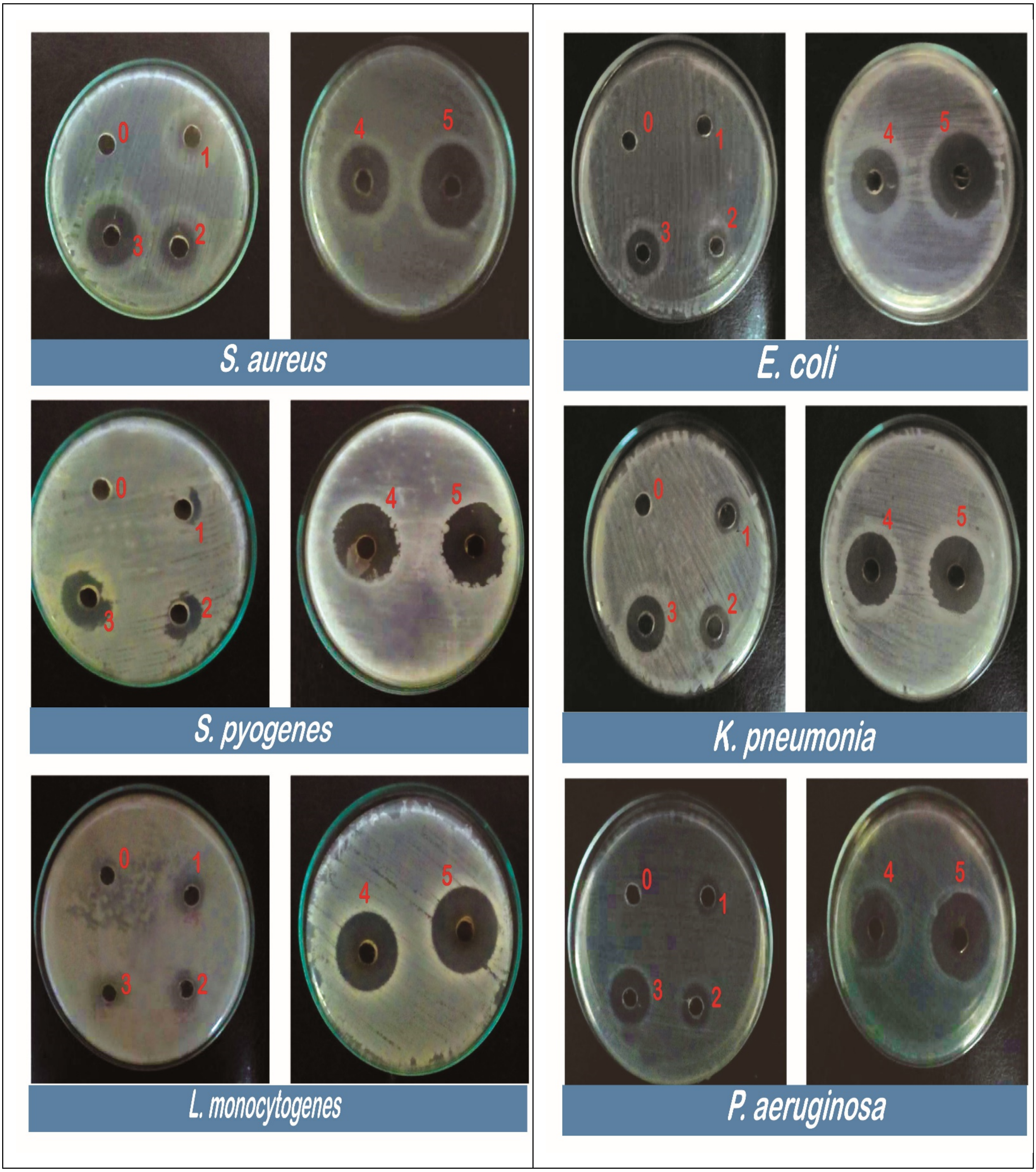

Figure S1. Antibacterial activity of HCPRE at different concentrations against Gram +ve bacteria. 0 ,1, 2, 3, 4 and 5 refer to (0, 25, 50, 100, 200 and 250 µg/mL, respectively).

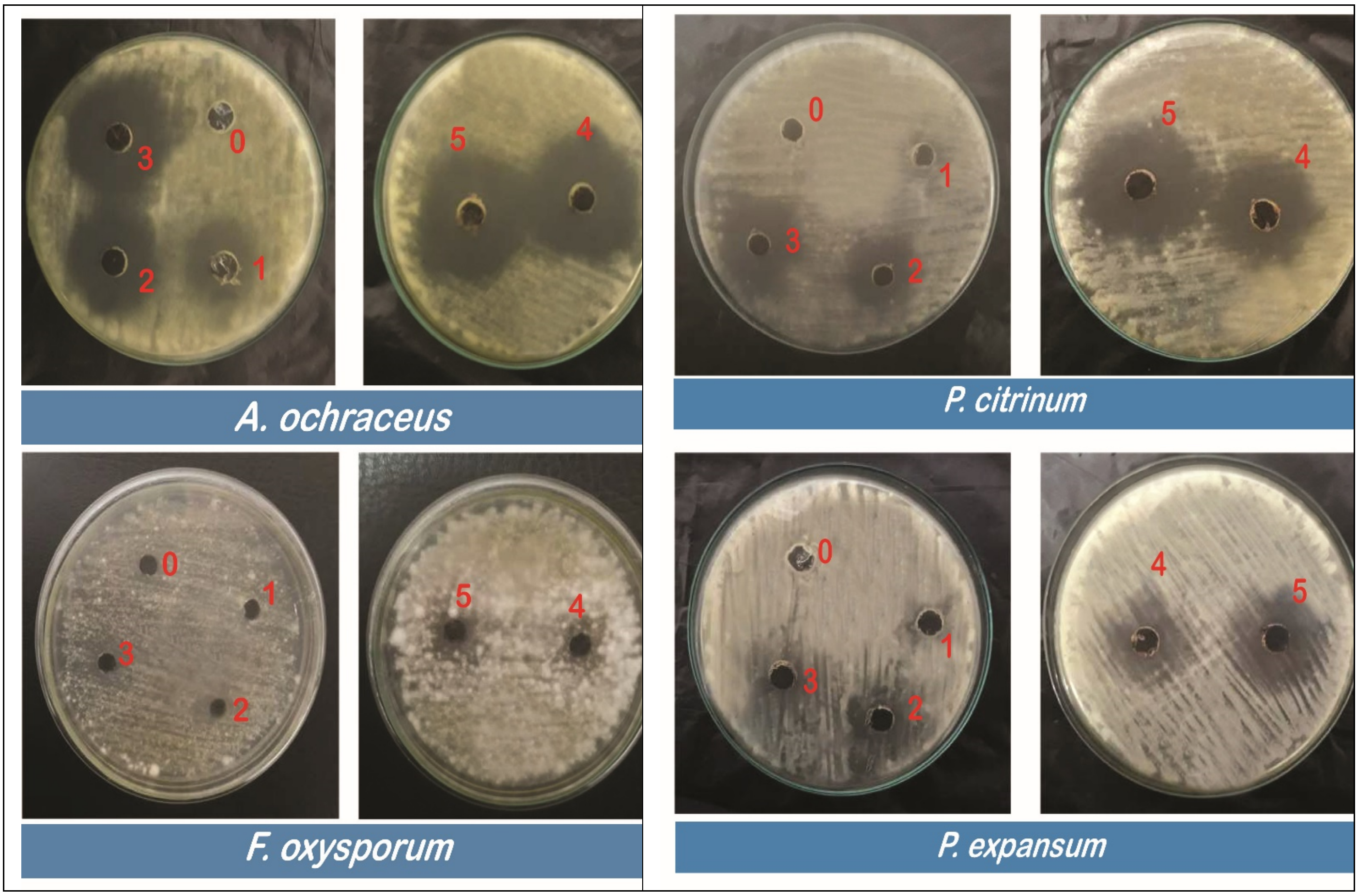

Figure S2. Antifungal activity of HCPRE at different concentrations against pathogenic fungi. 0, 1, 2, 3, 4 and 5 refer to (0,100,200,300,400 and 500 μg/mL, respectively).

My GC-MS Report

Low Mass(m/z):	45	Sample Name:	
High Mass(m/z):	500	Instrument Method:	C:\Xcalibur\methods\dr_zedan_15_5_2018_3.meth

Original Processing Method:

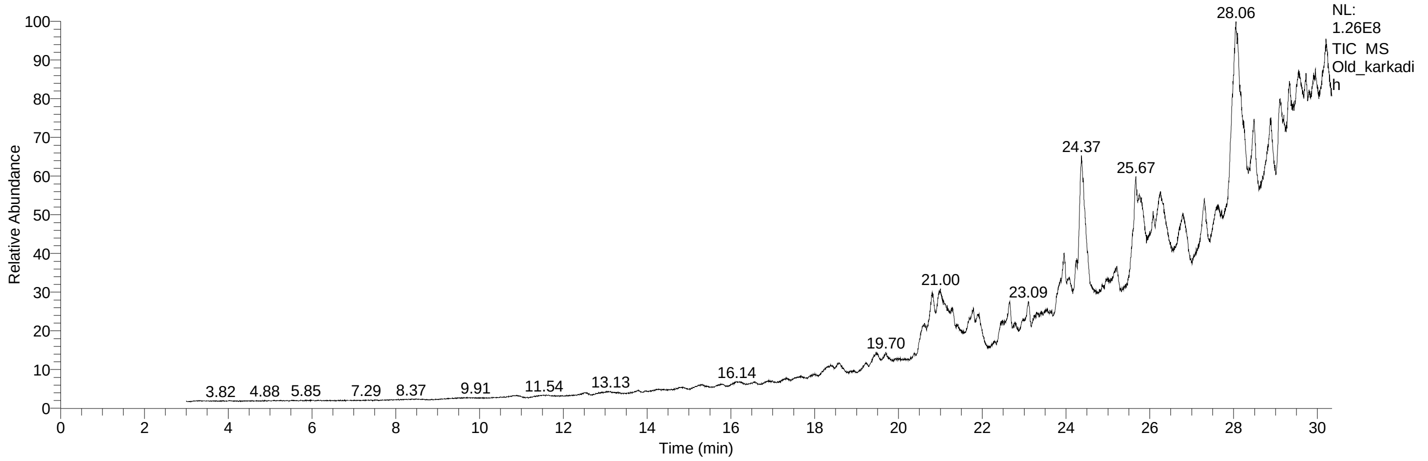

RT	Area %	Peak Height
10.88	0.14	538512.38
12.51	0.20	630104.23
12.92	0.19	642125.00
13.07	0.42	751828.45
13.79	0.15	562703.47
14.81	0.25	606298.63
15.28	0.53	1013902.24
15.79	0.26	700434.76
16.14	0.45	1009813.60
16.57	0.19	671097.30
16.89	0.23	608785.58
17.28	0.10	679623.87
17.34	0.13	734319.58
17.58	0.17	641155.85
17.67	0.15	691284.14
17.97	0.23	661513.92
18.30	0.40	1267231.33
18.37	0.22	1317397.10
18.59	0.55	1895282.49
19.22	0.31	1360199.60
19.46	0.91	2675821.18
19.70	0.49	2013635.02
20.37	0.09	754566.00
20.57	1.20	4029558.74
20.80	1.57	7502846.04
21.00	1.86	6099129.53
21.30	0.64	3482214.05
21.41	0.10	835523.49
21.78	2.24	7537882.87
21.90	1.86	6400354.79
22.29	0.15	813813.16
22.46	1.22	4763386.07
22.65	2.23	8653218.10
22.78	0.28	1620654.42
22.97	0.49	2853198.32
23.10	1.60	7623824.67
23.24	0.32	2432460.41
23.31	0.56	3022505.07
23.41	0.49	2541686.28
23.53	0.96	2643637.54
23.95	4.89	14368473.34
24.06	1.14	5302989.69
24.23	0.33	3262606.93
24.37	7.72	29616094.26

My GC-MS Report

RT	Area %	Peak Height
24.86	0.10	1060139.74
24.96	0.30	1628386.21
25.21	1.26	5307599.93
25.66	5.60	23599657.98
25.74	4.19	16154381.26
26.08	0.46	3962072.11
26.25	4.18	11297098.03
26.79	3.79	10355995.76
27.30	2.97	12504928.95
27.58	2.35	6486876.44
28.05	13.79	37196398.48
28.25	0.28	2810545.29
28.49	3.50	16893329.32
28.88	4.29	15829430.41
29.10	4.04	16621609.26
29.33	1.96	11039890.71
29.55	2.26	8700632.45
29.73	0.67	6080665.72
29.80	0.10	1309023.41
29.95	1.57	6611743.95
30.21	3.74	13877138.82

RT	Compound Name	Molecular Weight	Molecular Formula	Cas #	Area %	Library
10.88	DOTRIACONTANE	450	C32H66	544-85-4	0.14	WileyRegistry8e
10.88	2,2,3,3,4,4 HEXADEUTERO OCTADECANAL	274	C18H30D6O	56554-51-9	0.14	WileyRegistry8e
10.88	1-HEXADECANOL, 2-METHYL-	256	C17H36O	2490-48-4	0.14	WileyRegistry8e
10.88	1-Hexadecanol, 2-methyl-	256	C17H36O	2490-48-4	0.14	mainlib

Hit Spectrum	Compound Structure

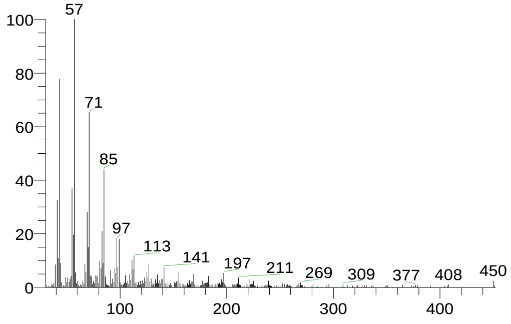

DOTRIACONTANE
Formula C32H66, MW 450, CAS# 544-85-4, Entry# 274478
AI3-52367

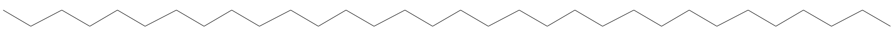

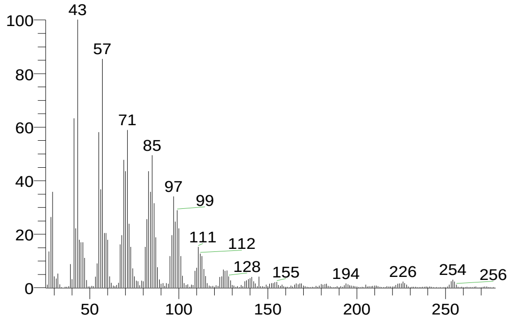

2,2,3,3,4,4 HEXADEUTERO OCTADECANAL
Formula C18H30D6O, MW 274, CAS# 56554-51-9, Entry# 159360

My GC-MS Report

Hit Spectrum	Compound Structure

1-HEXADECANOL, 2-METHYL-
Formula C17H36O, MW 256, CAS# 2490-48-4, Entry# 146911
2-METHYLHEXADECAN-1-OL

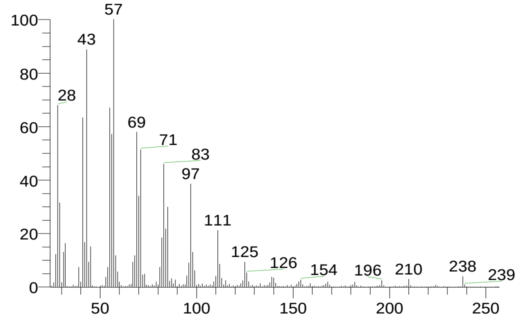

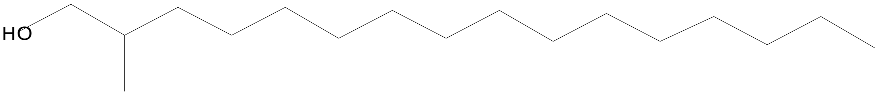

1-Hexadecanol, 2-methyl-
Formula C17H36O, MW 256, CAS# 2490-48-4, Entry# 20717
2-Methylhexadecan-1-ol

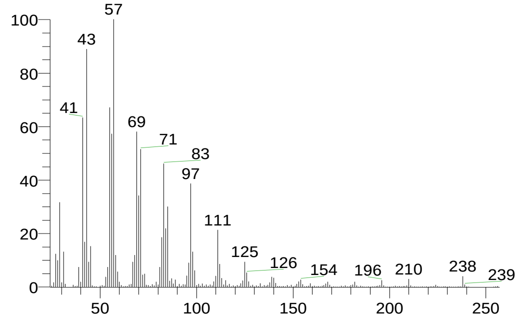

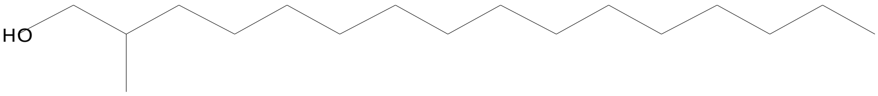

RT	Compound Name	Molecular Weight	Molecular Formula	Cas #	Area %	Library
12.51	2,2-DIDEUTERO OCTADECANAL	270	C18H34D2O	56555-07-8	0.20	WileyRegistry8e
12.51	2,2,3,3,4,4 HEXADEUTERO OCTADECANAL	274	C18H30D6O	56554-51-9	0.20	WileyRegistry8e
12.51	DOTRIACONTANE	450	C32H66	544-85-4	0.20	WileyRegistry8e
12.51	1-HEXADECANOL, 2-METHYL-	256	C17H36O	2490-48-4	0.20	WileyRegistry8e

Hit Spectrum	Compound Structure

2,2-DIDEUTERO OCTADECANAL
Formula C18H34D2O, MW 270, CAS# 56555-07-8, Entry# 159359

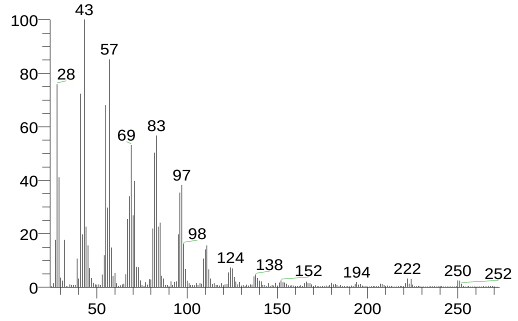

My GC-MS Report

Hit Spectrum | Compound Structure

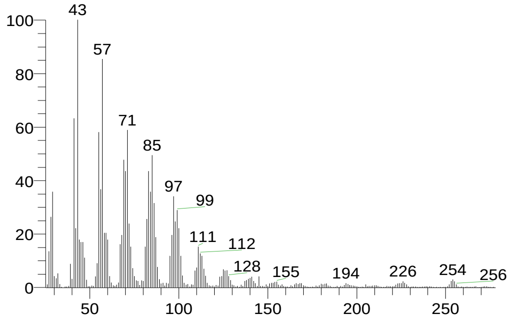

2,2,3,3,4,4 HEXADEUTERO OCTADECANAL
Formula C18H30D6O, MW 274, CAS# 56554-51-9, Entry# 159360

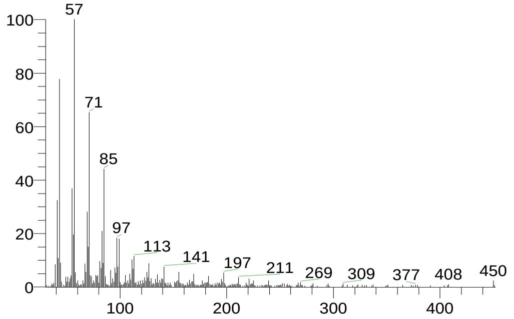

DOTRIACONTANE
Formula C32H66, MW 450, CAS# 544-85-4, Entry# 274478
AI3-52367

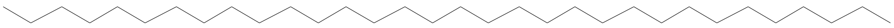

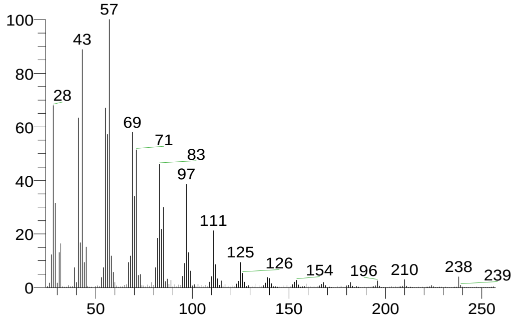

1-HEXADECANOL, 2-METHYL-
Formula C17H36O, MW 256, CAS# 2490-48-4, Entry# 146911
2-METHYLHEXADECAN-1-OL

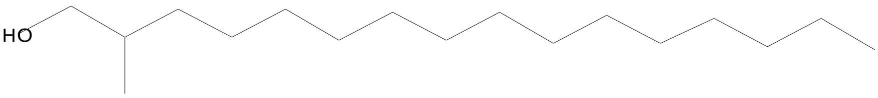

RT	Compound Name	Molecular Weight	Molecular Formula	Cas #	Area %	Library
12.92	DOTRIACONTANE	450	C32H66	544-85-4	0.19	WileyRegistry8e
12.92	1-HEXADECANOL, 2-METHYL-	256	C17H36O	2490-48-4	0.19	WileyRegistry8e
12.92	2,2-DIDEUTERO OCTADECANAL	270	C18H34D2O	56555-07-8	0.19	WileyRegistry8e
12.92	2,2,3,3,4,4 HEXADEUTERO OCTADECANAL	274	C18H30D6O	56554-51-9	0.19	WileyRegistry8e

My GC-MS Report

Hit Spectrum | Compound Structure

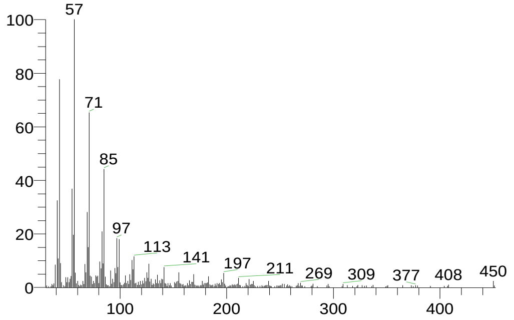

DOTRIACONTANE
Formula C32H66, MW 450, CAS# 544-85-4, Entry# 274478
AI3-52367

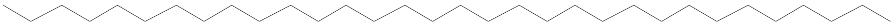

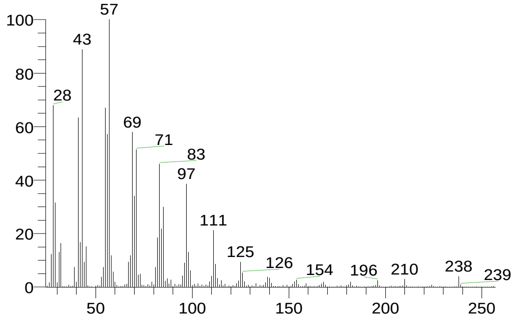

1-HEXADECANOL, 2-METHYL-
Formula C17H36O, MW 256, CAS# 2490-48-4, Entry# 146911
2-METHYLHEXADECAN-1-OL

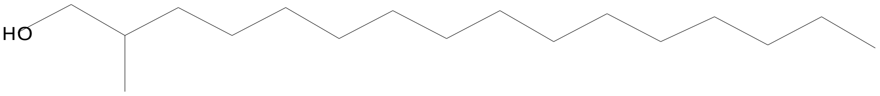

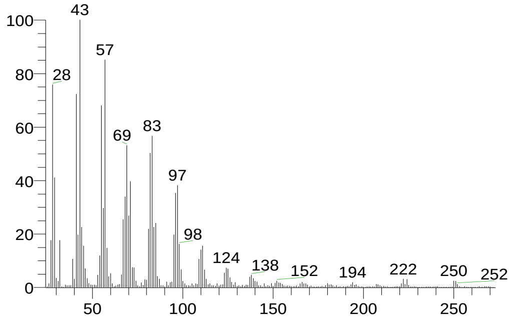

2,2-DIDEUTERO OCTADECANAL
Formula C18H34D2O, MW 270, CAS# 56555-07-8, Entry# 159359

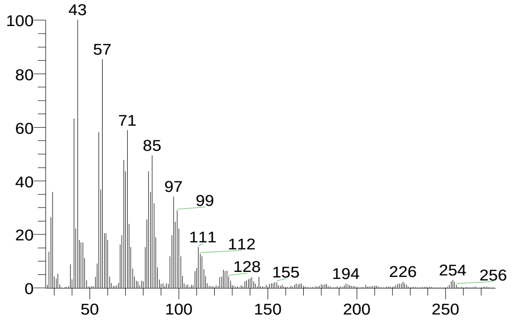

2,2,3,3,4,4 HEXADEUTERO OCTADECANAL
Formula C18H30D6O, MW 274, CAS# 56554-51-9, Entry# 159360

RT	Compound Name	Molecular Weight	Molecular Formula	Cas #	Area %	Library
13.07	DOTRIACONTANE	450	C32H66	544-85-4	0.42	WileyRegistry8e
13.07	2,2-DIDEUTERO OCTADECANAL	270	C18H34D2O	56555-07-8	0.42	WileyRegistry8e
13.07	2,2,3,3,4,4 HEXADEUTERO OCTADECANAL	274	C18H30D6O	56554-51-9	0.42	WileyRegistry8e
13.07	Hexadecane, 1,1-bis(dodecyloxy)-	594	C40H82O2	56554-64-4	0.42	mainlib

My GC-MS Report

Hit Spectrum | Compound Structure

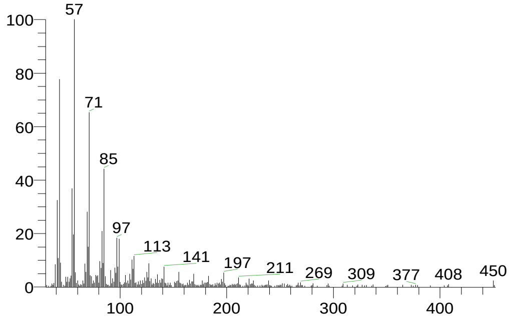

DOTRIACONTANE
Formula C32H66, MW 450, CAS# 544-85-4, Entry# 274478
AI3-52367

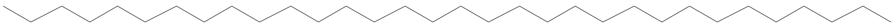

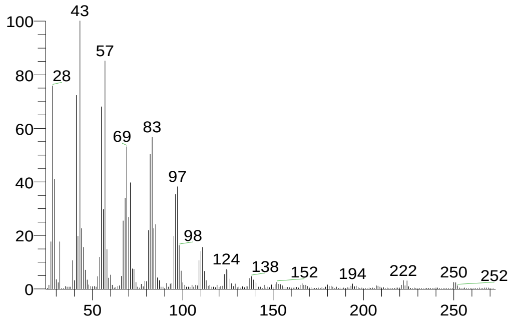

2,2-DIDEUTERO OCTADECANAL
Formula C18H34D2O, MW 270, CAS# 56555-07-8, Entry# 159359

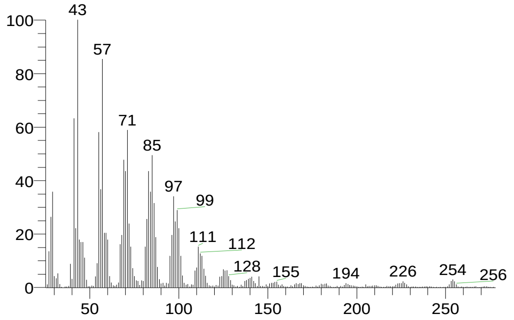

2,2,3,3,4,4 HEXADEUTERO OCTADECANAL
Formula C18H30D6O, MW 274, CAS# 56554-51-9, Entry# 159360

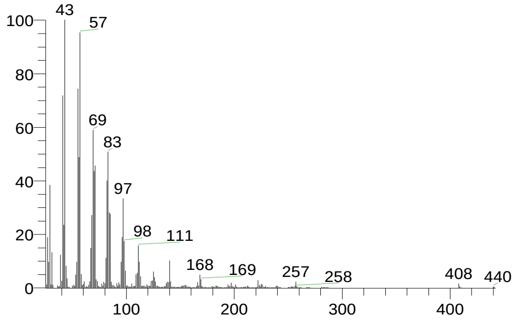

Hexadecane, 1,1-bis(dodecyloxy)-
Formula C40H82O2, MW 594, CAS# 56554-64-4, Entry# 6971
1,1-Bis(dodecyloxy)hexadecane #

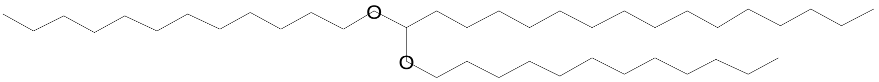

RT	Compound Name	Molecular Weight	Molecular Formula	Cas #	Area %	Library
13.79	2,2,3,3,4,4 HEXADEUTERO OCTADECANAL	274	C18H30D6O	56554-51-9	0.15	WileyRegistry8e
13.79	1-HEXADECANOL, 2-METHYL-	256	C17H36O	2490-48-4	0.15	WileyRegistry8e
13.79	DOTRIACONTANE	450	C32H66	544-85-4	0.15	WileyRegistry8e
13.79	1-Hexadecanol, 2-methyl-	256	C17H36O	2490-48-4	0.15	mainlib

My GC-MS Report

Hit Spectrum | Compound Structure

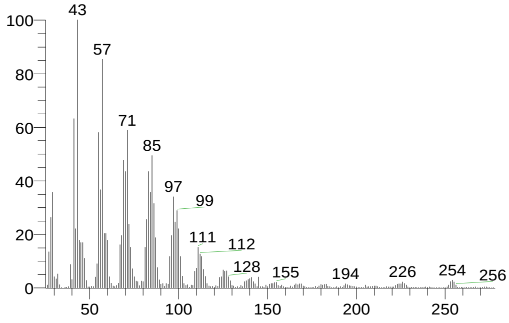

2,2,3,3,4,4 HEXADEUTERO OCTADECANAL
Formula C18H30D6O, MW 274, CAS# 56554-51-9, Entry# 159360

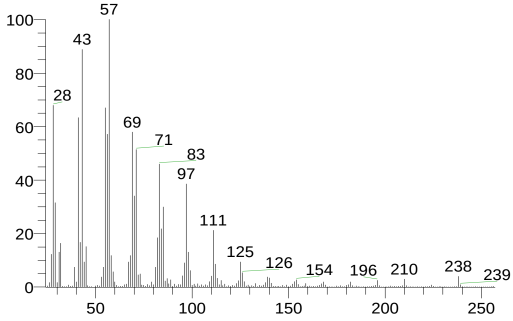

1-HEXADECANOL, 2-METHYL-
Formula C17H36O, MW 256, CAS# 2490-48-4, Entry# 146911
2-METHYLHEXADECAN-1-OL

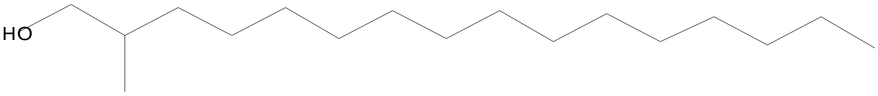

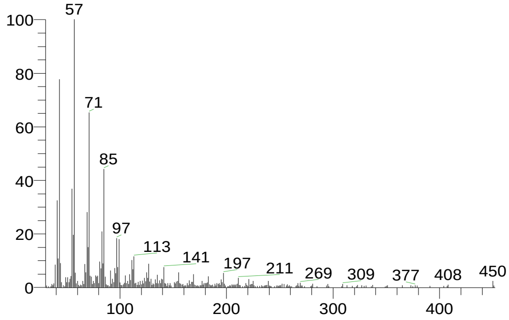

DOTRIACONTANE
Formula C32H66, MW 450, CAS# 544-85-4, Entry# 274478
AI3-52367

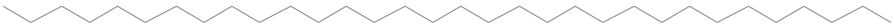

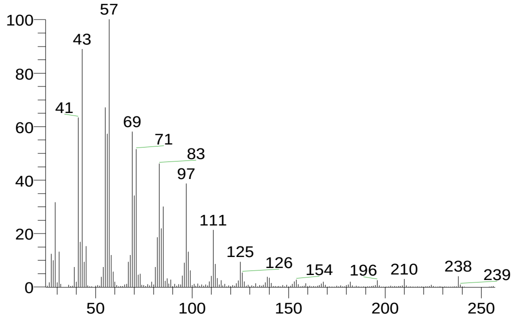

1-Hexadecanol, 2-methyl-
Formula C17H36O, MW 256, CAS# 2490-48-4, Entry# 20717
2-Methylhexadecan-1-ol

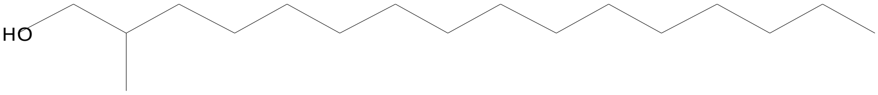

RT	Compound Name	Molecular Weight	Molecular Formula	Cas #	Area %	Library
14.81	2,2-DIDEUTERO OCTADECANAL	270	C18H34D2O	56555-07-8	0.25	WileyRegistry8e
14.81	DOTRIACONTANE	450	C32H66	544-85-4	0.25	WileyRegistry8e
14.81	1,3,5-TRIAZINE-2,4-DIAMINE, 6-CHLORO-N-ETHYL-	173	C5H8ClN5	1007-28-9	0.25	WileyRegistry8e
14.81	14-á-H-PREGNA	288	C21H36	NA	0.25	WileyRegistry8e

My GC-MS Report

Hit Spectrum | Compound Structure

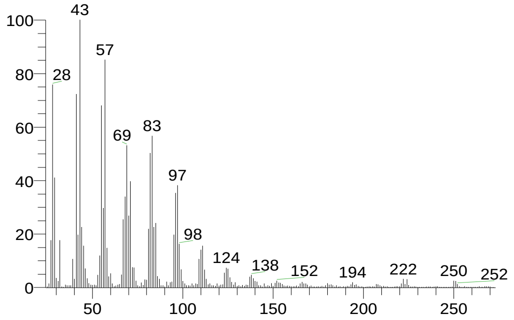

2,2-DIDEUTERO OCTADECANAL
Formula C18H34D2O, MW 270, CAS# 56555-07-8, Entry# 159359

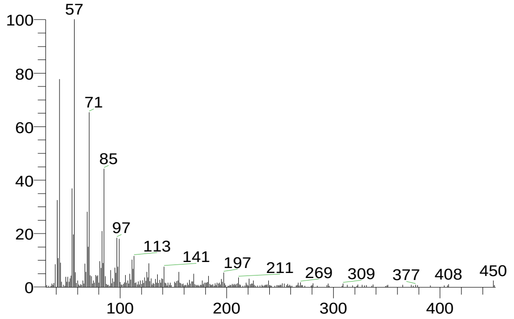

DOTRIACONTANE
Formula C32H66, MW 450, CAS# 544-85-4, Entry# 274478
AI3-52367

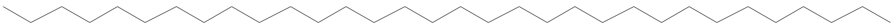

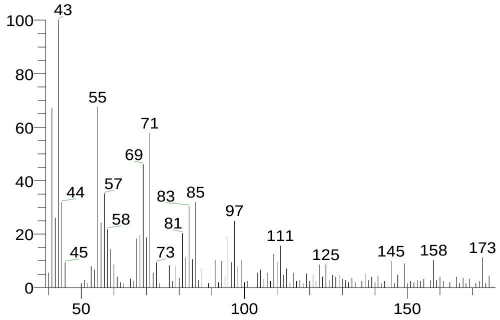

1,3,5-TRIAZINE-2,4-DIAMINE, 6-CHLORO-N-ETHYL-
Formula C5H8ClN5, MW 173, CAS# 1007-28-9, Entry# 56163
1,3, 5-TRIAZINE-2,4-DIAMINE, 6-CHLORO-N-ETHYL-

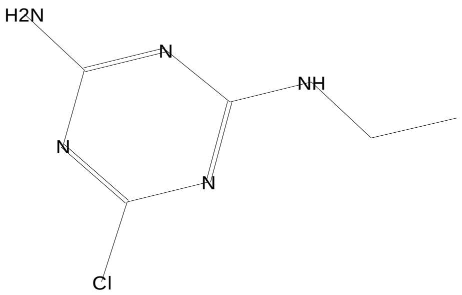

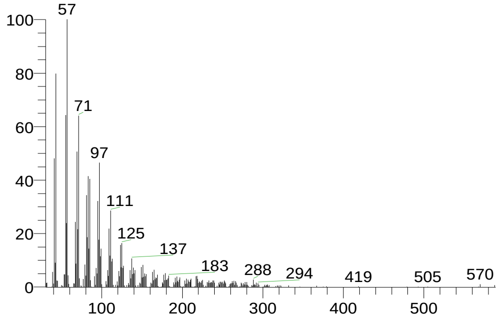

14-á-H-PREGNA
Formula C21H36, MW 288, CAS# NA, Entry# 178939
14-á-PREGNA

RT	Compound Name	Molecular Weight	Molecular Formula	Cas #	Area %	Library
15.28	2,2-DIDEUTERO OCTADECANAL	270	C18H34D2O	56555-07-8	0.53	WileyRegistry8e
15.28	DOTRIACONTANE	450	C32H66	544-85-4	0.53	WileyRegistry8e
15.28	12-Methyl-E,E-2,13-octadecadien-1-ol	280	C19H36O	NA	0.53	mainlib
15.28	7-Methyl-Z-tetradecen-1-ol acetate	268	C17H32O2	NA	0.53	mainlib

My GC-MS Report

Hit Spectrum	Compound Structure

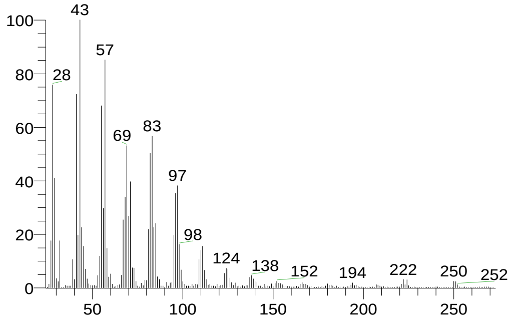

2,2-DIDEUTERO OCTADECANAL
Formula C18H34D2O, MW 270, CAS# 56555-07-8, Entry# 159359

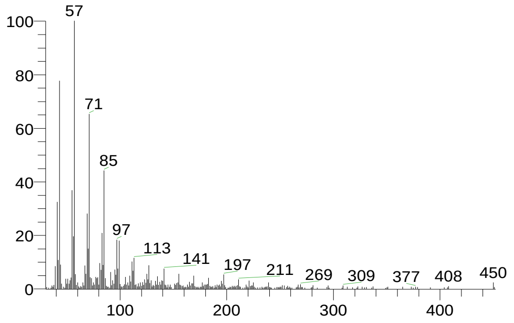

DOTRIACONTANE
Formula C32H66, MW 450, CAS# 544-85-4, Entry# 274478
AI3-52367

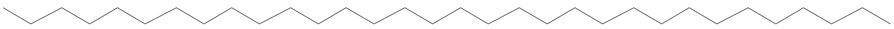

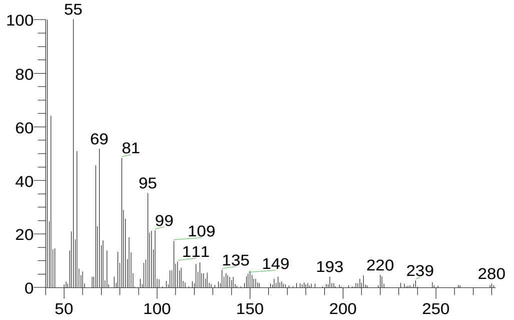

12-Methyl-E,E-2,13-octadecadien-1-ol
Formula C19H36O, MW 280, CAS# NA, Entry# 16455

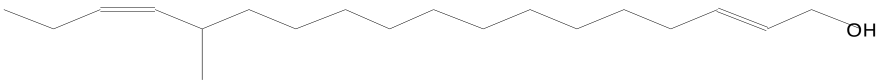

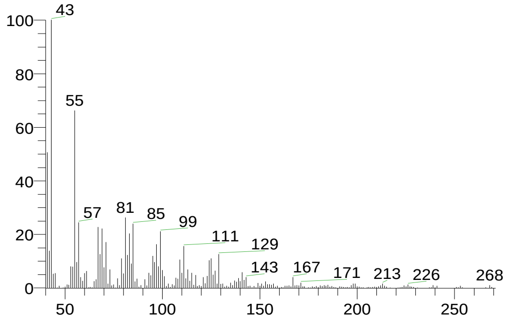

7-Methyl-Z-tetradecen-1-ol acetate
Formula C17H32O2, MW 268, CAS# NA, Entry# 6342

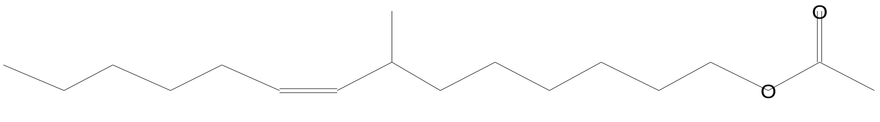

RT	Compound Name	Molecular Weight	Molecular Formula	Cas #	Area %	Library
15.79	2,2-DIDEUTERO OCTADECANAL	270	C18H34D2O	56555-07-8	0.26	WileyRegistry8e
15.79	1-HEXADECANOL, 2-METHYL-	256	C17H36O	2490-48-4	0.26	WileyRegistry8e
15.79	14-á-H-PREGNA	288	C21H36	NA	0.26	WileyRegistry8e
15.79	1-Hexadecanol, 2-methyl-	256	C17H36O	2490-48-4	0.26	mainlib

My GC-MS Report

Hit Spectrum | Compound Structure

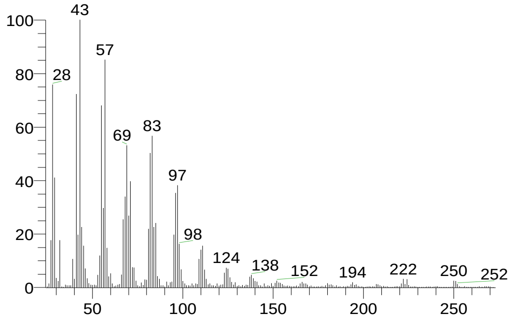

2,2-DIDEUTERO OCTADECANAL
Formula C18H34D2O, MW 270, CAS# 56555-07-8, Entry# 159359

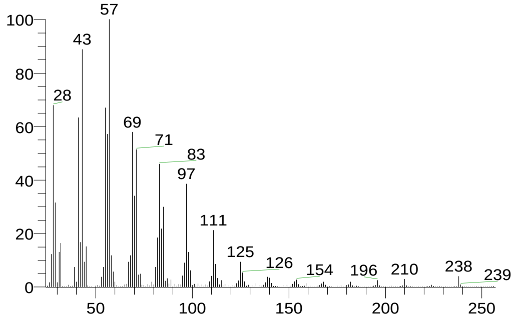

1-HEXADECANOL, 2-METHYL-
Formula C17H36O, MW 256, CAS# 2490-48-4, Entry# 146911
2-METHYLHEXADECAN-1-OL

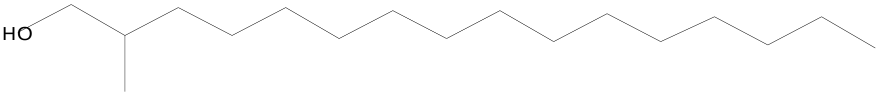

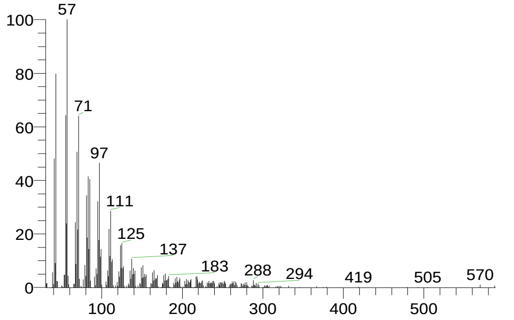

14-á-H-PREGNA
Formula C21H36, MW 288, CAS# NA, Entry# 178939
14-á-PREGNA

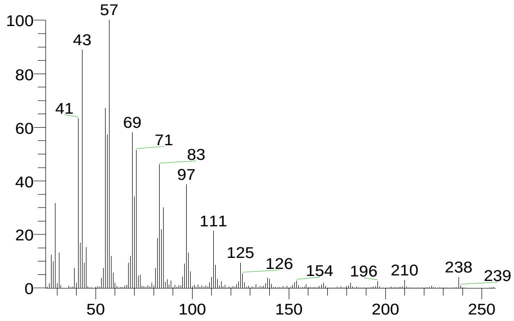

1-Hexadecanol, 2-methyl-
Formula C17H36O, MW 256, CAS# 2490-48-4, Entry# 20717
2-Methylhexadecan-1-ol

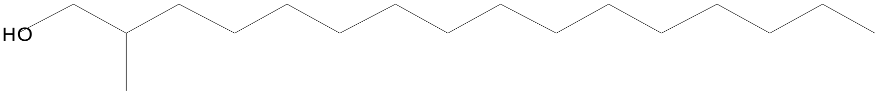

RT	Compound Name	Molecular Weight	Molecular Formula	Cas #	Area %	Library
16.14	2,2-DIDEUTERO OCTADECANAL	270	C18H34D2O	56555-07-8	0.45	WileyRegistry8e
16.14	DOTRIACONTANE	450	C32H66	544-85-4	0.45	WileyRegistry8e
16.14	1-HEXADECANOL, 2-METHYL-	256	C17H36O	2490-48-4	0.45	WileyRegistry8e
16.14	2,2,3,3,4,4 HEXADEUTERO OCTADECANAL	274	C18H30D6O	56554-51-9	0.45	WileyRegistry8e

My GC-MS Report

Hit Spectrum | Compound Structure

2,2-DIDEUTERO OCTADECANAL
Formula C18H34D2O, MW 270, CAS# 56555-07-8, Entry# 159359

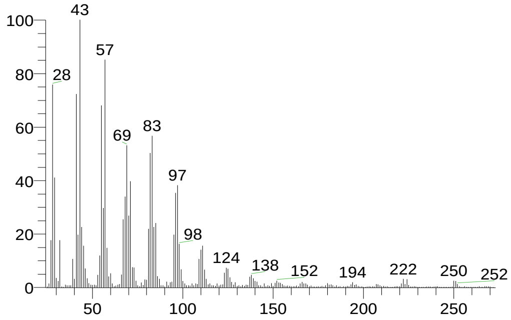

DOTRIACONTANE
Formula C32H66, MW 450, CAS# 544-85-4, Entry# 274478
AI3-52367

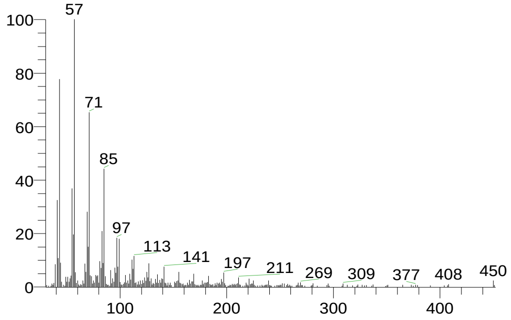

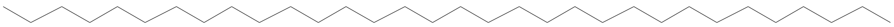

1-HEXADECANOL, 2-METHYL-
Formula C17H36O, MW 256, CAS# 2490-48-4, Entry# 146911
2-METHYLHEXADECAN-1-OL

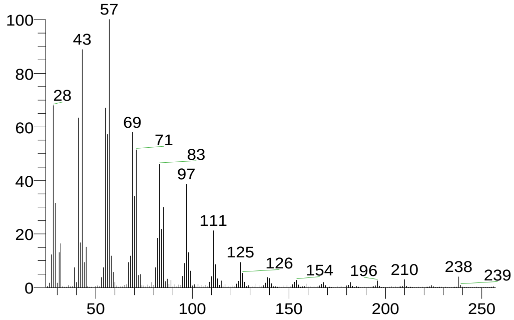

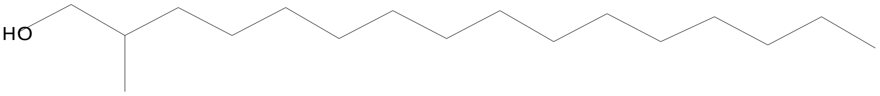

2,2,3,3,4,4 HEXADEUTERO OCTADECANAL
Formula C18H30D6O, MW 274, CAS# 56554-51-9, Entry# 159360

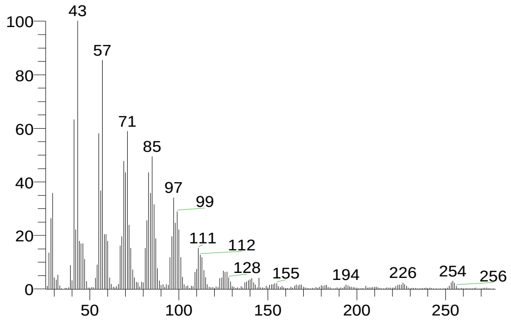

RT	Compound Name	Molecular Weight	Molecular Formula	Cas #	Area %	Library
16.57	DOTRIACONTANE	450	C32H66	544-85-4	0.19	WileyRegistry8e
16.57	14-á-H-PREGNA	288	C21H36	NA	0.19	WileyRegistry8e
16.57	2,2-DIDEUTERO OCTADECANAL	270	C18H34D2O	56555-07-8	0.19	WileyRegistry8e
16.57	1-HEXADECANOL, 2-METHYL-	256	C17H36O	2490-48-4	0.19	WileyRegistry8e

My GC-MS Report

Hit Spectrum | Compound Structure

DOTRIACONTANE
Formula C32H66, MW 450, CAS# 544-85-4, Entry# 274478
AI3-52367

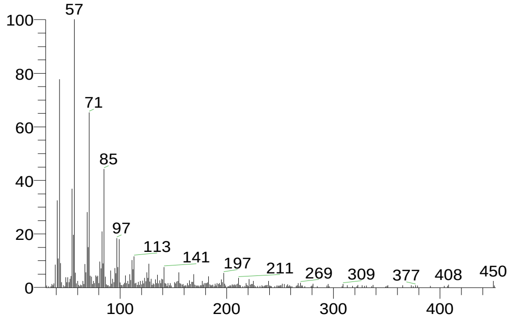

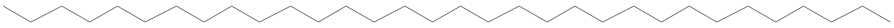

14-á-H-PREGNA
Formula C21H36, MW 288, CAS# NA, Entry# 178939
14-á-PREGNA

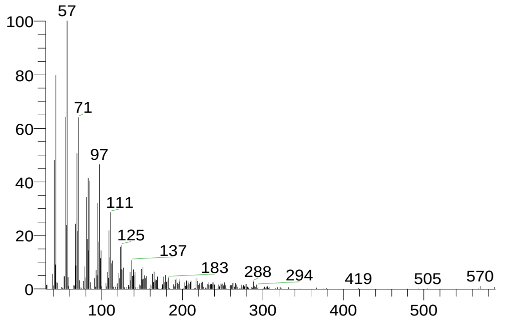

2,2-DIDEUTERO OCTADECANAL
Formula C18H34D2O, MW 270, CAS# 56555-07-8, Entry# 159359

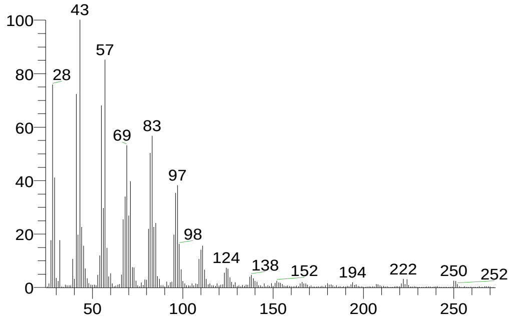

1-HEXADECANOL, 2-METHYL-
Formula C17H36O, MW 256, CAS# 2490-48-4, Entry# 146911
2-METHYLHEXADECAN-1-OL

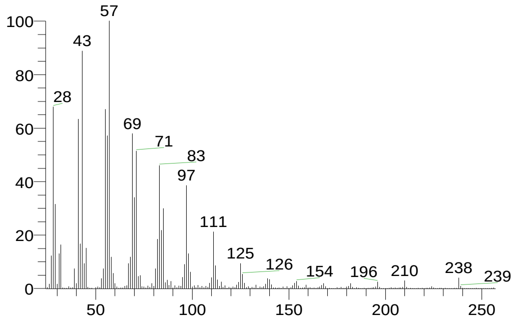

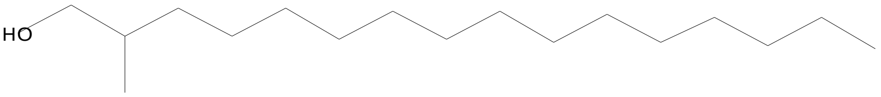

RT	Compound Name	Molecular Weight	Molecular Formula	Cas #	Area %	Library
16.89	DOTRIACONTANE	450	C32H66	544-85-4	0.23	WileyRegistry8e
16.89	14-á-H-PREGNA	288	C21H36	NA	0.23	WileyRegistry8e
16.89	tert-Hexadecanethiol	258	C16H34S	25360-09-2	0.23	mainlib
16.89	2,2,3,3,4,4 HEXADEUTERO OCTADECANAL	274	C18H30D6O	56554-51-9	0.23	WileyRegistry8e

My GC-MS Report

Hit Spectrum | Compound Structure

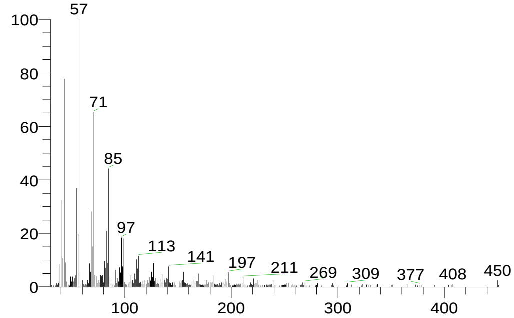

DOTRIACONTANE
Formula C32H66, MW 450, CAS# 544-85-4, Entry# 274478
AI3-52367

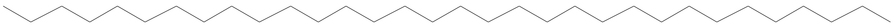

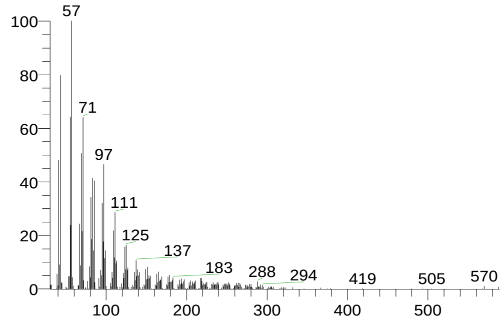

14-á-H-PREGNA
Formula C21H36, MW 288, CAS# NA, Entry# 178939
14-á-PREGNA

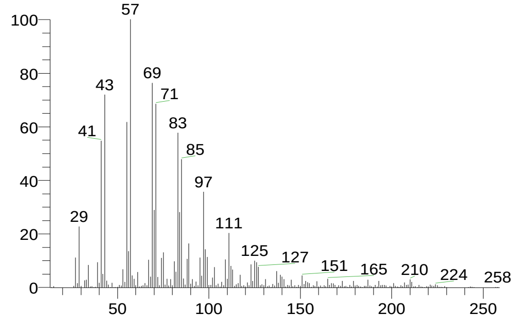

tert-Hexadecanethiol
Formula C16H34S, MW 258, CAS# 25360-09-2, Entry# 21483
1,1-Dimethyltetradecyl hydrosulfide #

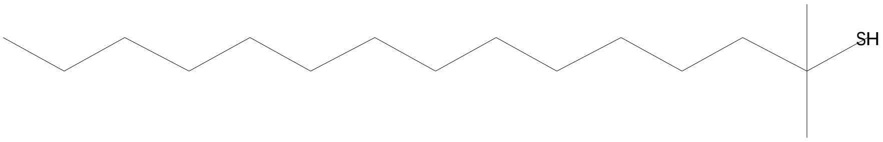

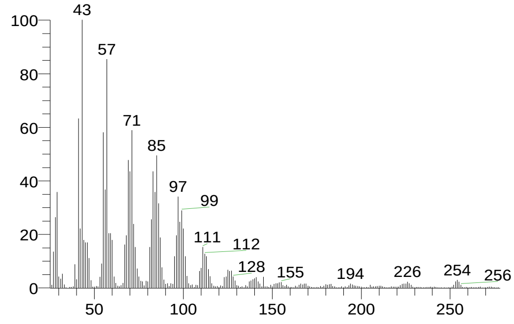

2,2,3,3,4,4 HEXADEUTERO OCTADECANAL
Formula C18H30D6O, MW 274, CAS# 56554-51-9, Entry# 159360

RT	Compound Name	Molecular Weight	Molecular Formula	Cas #	Area %	Library
17.28	DOTRIACONTANE	450	C32H66	544-85-4	0.10	WileyRegistry8e
17.28	2,2-DIDEUTERO OCTADECANAL	270	C18H34D2O	56555-07-8	0.10	WileyRegistry8e
17.28	14-á-H-PREGNA	288	C21H36	NA	0.10	WileyRegistry8e
17.28	2,2,3,3,4,4 HEXADEUTERO OCTADECANAL	274	C18H30D6O	56554-51-9	0.10	WileyRegistry8e

My GC-MS Report

Hit Spectrum	Compound Structure

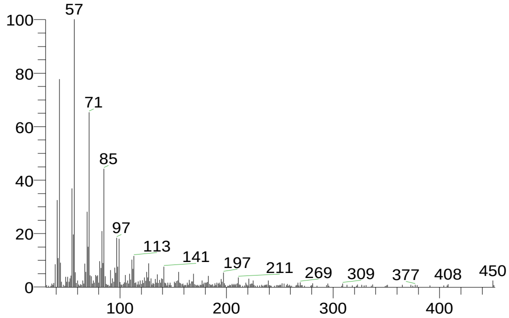

DOTRIACONTANE
Formula C32H66, MW 450, CAS# 544-85-4, Entry# 274478
AI3-52367

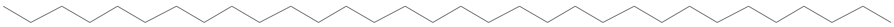

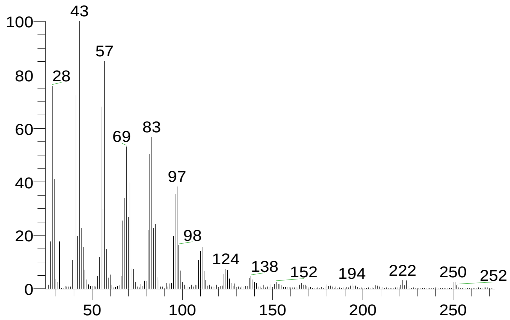

2,2-DIDEUTERO OCTADECANAL
Formula C18H34D2O, MW 270, CAS# 56555-07-8, Entry# 159359

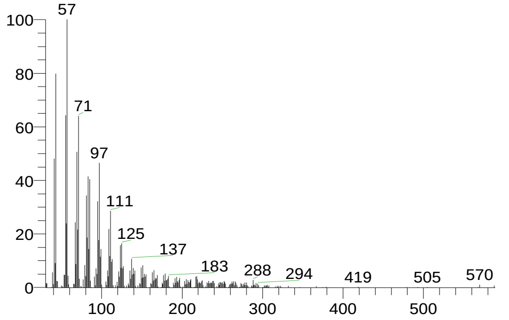

14-á-H-PREGNA
Formula C21H36, MW 288, CAS# NA, Entry# 178939
14-á-PREGNA

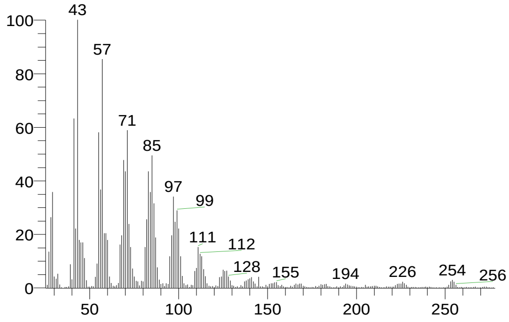

2,2,3,3,4,4 HEXADEUTERO OCTADECANAL
Formula C18H30D6O, MW 274, CAS# 56554-51-9, Entry# 159360

RT	Compound Name	Molecular Weight	Molecular Formula	Cas #	Area %	Library
17.34	DOTRIACONTANE	450	C32H66	544-85-4	0.13	WileyRegistry8e
17.34	2,2-DIDEUTERO OCTADECANAL	270	C18H34D2O	56555-07-8	0.13	WileyRegistry8e
17.34	14-á-H-PREGNA	288	C21H36	NA	0.13	WileyRegistry8e
17.34	1-Hexadecanol, 2-methyl-	256	C17H36O	2490-48-4	0.13	mainlib

My GC-MS Report

Hit Spectrum | Compound Structure

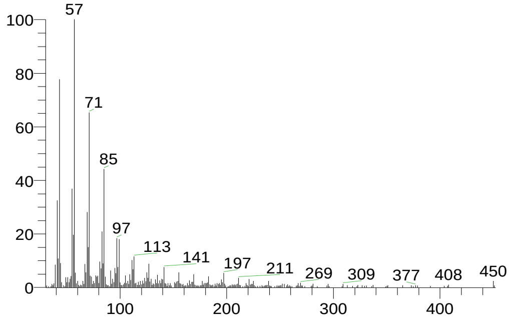

DOTRIACONTANE
Formula C32H66, MW 450, CAS# 544-85-4, Entry# 274478
AI3-52367

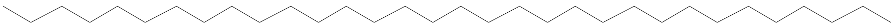

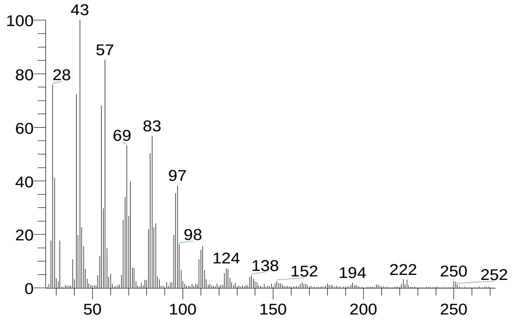

2,2-DIDEUTERO OCTADECANAL
Formula C18H34D2O, MW 270, CAS# 56555-07-8, Entry# 159359

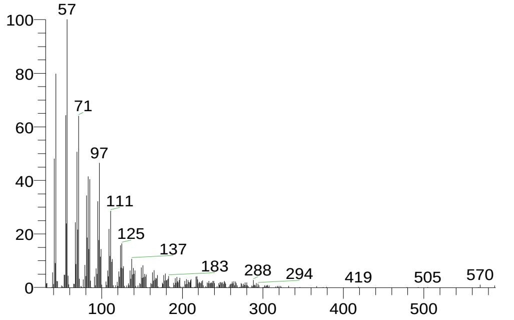

14-á-H-PREGNA
Formula C21H36, MW 288, CAS# NA, Entry# 178939
14-á-PREGNA

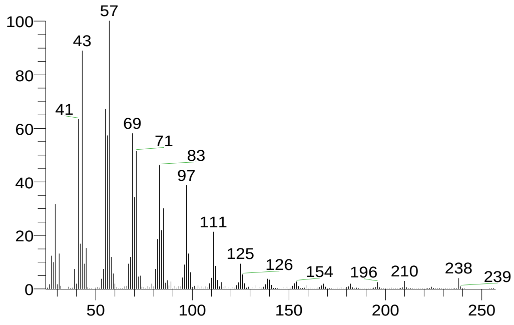

1-Hexadecanol, 2-methyl-
Formula C17H36O, MW 256, CAS# 2490-48-4, Entry# 20717
2-Methylhexadecan-1-ol

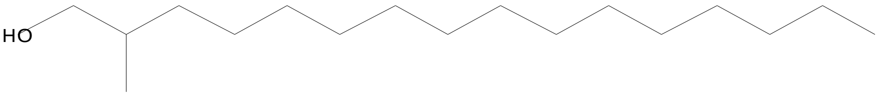

RT	Compound Name	Molecular Weight	Molecular Formula	Cas #	Area %	Library
17.58	DOTRIACONTANE	450	C32H66	544-85-4	0.17	WileyRegistry8e
17.58	2,2-DIDEUTERO OCTADECANAL	270	C18H34D2O	56555-07-8	0.17	WileyRegistry8e
17.58	14-á-H-PREGNA	288	C21H36	NA	0.17	WileyRegistry8e
17.58	1-NONADECENE	266	C19H38	18435-45-5	0.17	WileyRegistry8e

My GC-MS Report

Hit Spectrum	Compound Structure

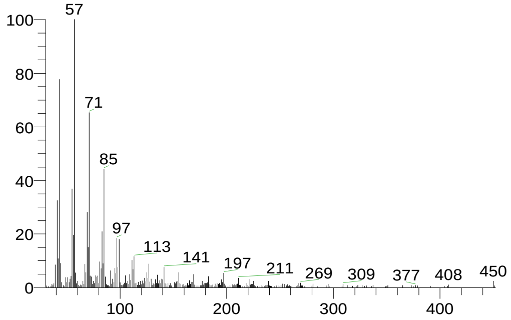

DOTRIACONTANE
Formula C32H66, MW 450, CAS# 544-85-4, Entry# 274478
AI3-52367

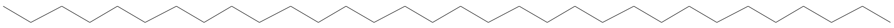

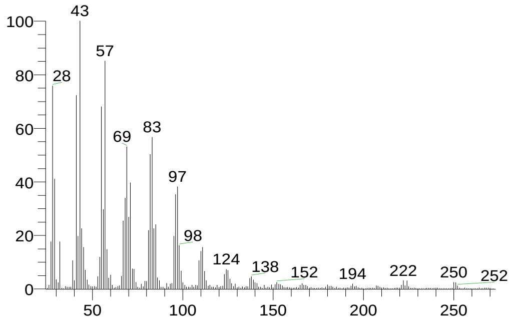

2,2-DIDEUTERO OCTADECANAL
Formula C18H34D2O, MW 270, CAS# 56555-07-8, Entry# 159359

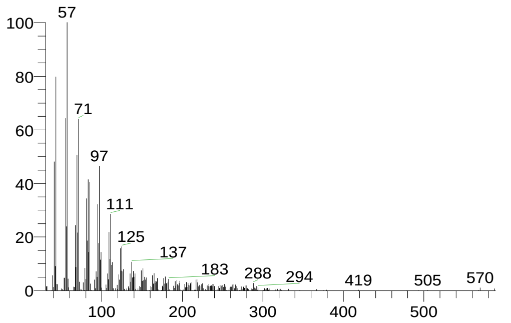

14-á-H-PREGNA
Formula C21H36, MW 288, CAS# NA, Entry# 178939
14-á-PREGNA

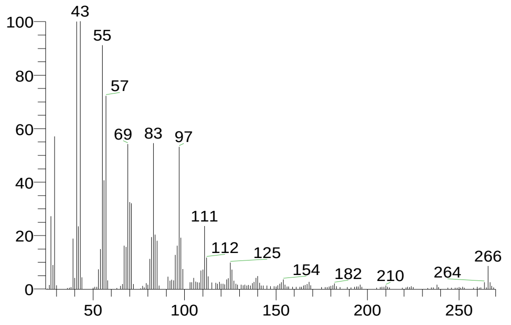

1-NONADECENE
Formula C19H38, MW 266, CAS# 18435-45-5, Entry# 157387
AI3-36475

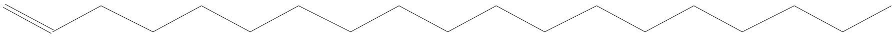

RT	Compound Name	Molecular Weight	Molecular Formula	Cas #	Area %	Library
17.67	DOTRIACONTANE	450	C32H66	544-85-4	0.15	WileyRegistry8e
17.67	2,2-DIDEUTERO OCTADECANAL	270	C18H34D2O	56555-07-8	0.15	WileyRegistry8e
17.67	14-á-H-PREGNA	288	C21H36	NA	0.15	WileyRegistry8e
17.67	2,2,3,3,4,4 HEXADEUTERO OCTADECANAL	274	C18H30D6O	56554-51-9	0.15	WileyRegistry8e

My GC-MS Report

Hit Spectrum | Compound Structure

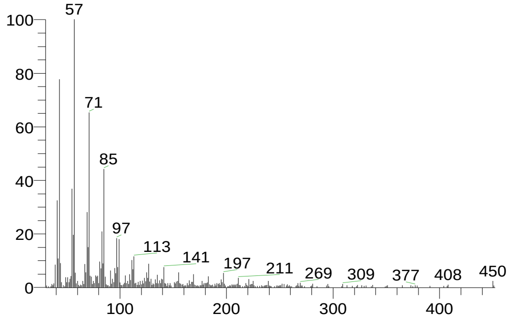

DOTRIACONTANE
Formula C32H66, MW 450, CAS# 544-85-4, Entry# 274478
AI3-52367

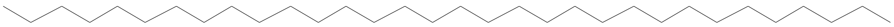

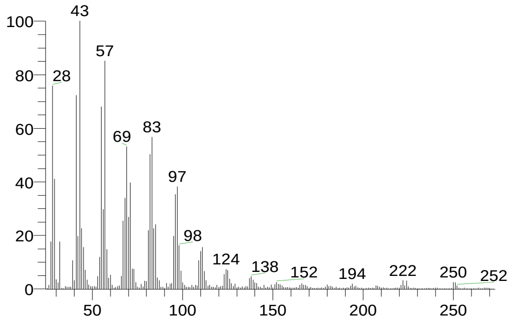

2,2-DIDEUTERO OCTADECANAL
Formula C18H34D2O, MW 270, CAS# 56555-07-8, Entry# 159359

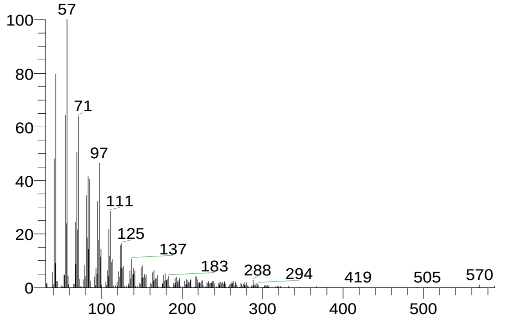

14-á-H-PREGNA
Formula C21H36, MW 288, CAS# NA, Entry# 178939
14-á-PREGNA

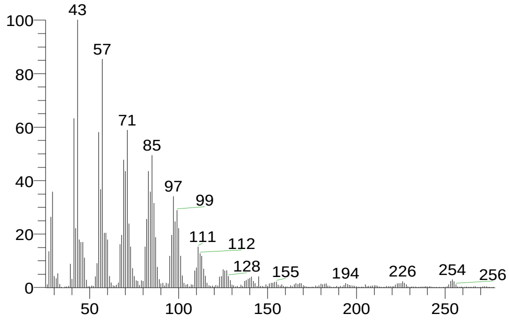

2,2,3,3,4,4 HEXADEUTERO OCTADECANAL
Formula C18H30D6O, MW 274, CAS# 56554-51-9, Entry# 159360

RT	Compound Name	Molecular Weight	Molecular Formula	Cas #	Area %	Library
17.97	2,2-DIDEUTERO OCTADECANAL	270	C18H34D2O	56555-07-8	0.23	WileyRegistry8e
17.97	14-á-H-PREGNA	288	C21H36	NA	0.23	WileyRegistry8e
17.97	1-HEXADECANOL, 2-METHYL-	256	C17H36O	2490-48-4	0.23	WileyRegistry8e
17.97	1-NONADECENE	266	C19H38	18435-45-5	0.23	WileyRegistry8e

My GC-MS Report

Hit Spectrum | Compound Structure

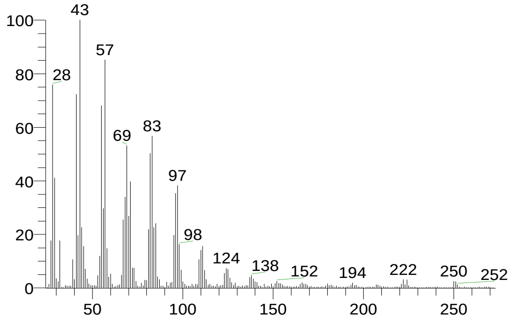

2,2-DIDEUTERO OCTADECANAL
Formula C18H34D2O, MW 270, CAS# 56555-07-8, Entry# 159359

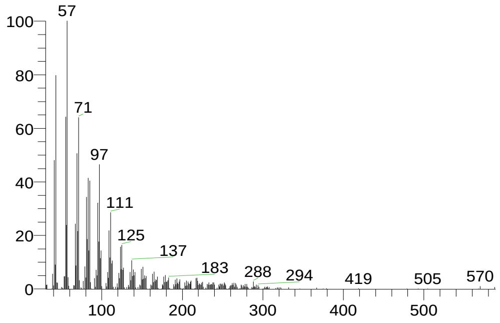

14-á-H-PREGNA
Formula C21H36, MW 288, CAS# NA, Entry# 178939
14-á-PREGNA

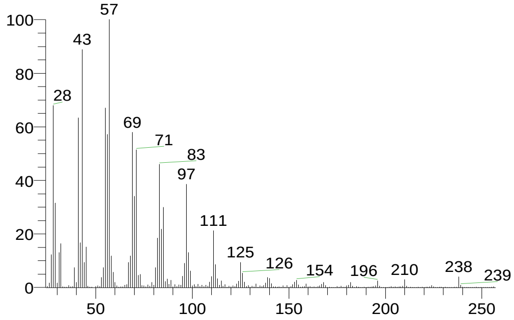

1-HEXADECANOL, 2-METHYL-
Formula C17H36O, MW 256, CAS# 2490-48-4, Entry# 146911
2-METHYLHEXADECAN-1-OL

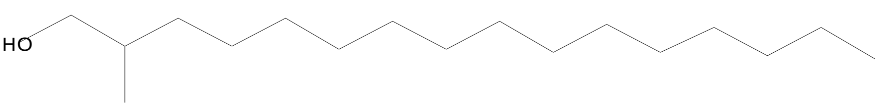

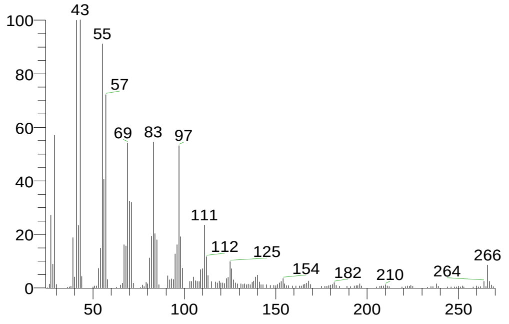

1-NONADECENE
Formula C19H38, MW 266, CAS# 18435-45-5, Entry# 157387
AI3-36475

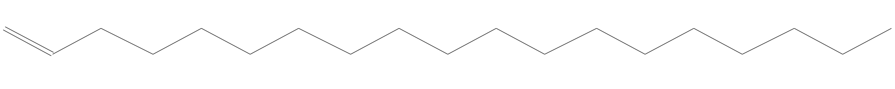

RT	Compound Name	Molecular Weight	Molecular Formula	Cas #	Area %	Library
18.30	14-á-H-PREGNA	288	C21H36	NA	0.40	WileyRegistry8e
18.30	01297107001 TETRANEURIN - A - DIOL	280	C15H20O5	NA	0.40	WileyRegistry8e
18.30	DOTRIACONTANE	450	C32H66	544-85-4	0.40	WileyRegistry8e
18.30	2,2-DIDEUTERO OCTADECANAL	270	C18H34D2O	56555-07-8	0.40	WileyRegistry8e

My GC-MS Report

Hit Spectrum | Compound Structure

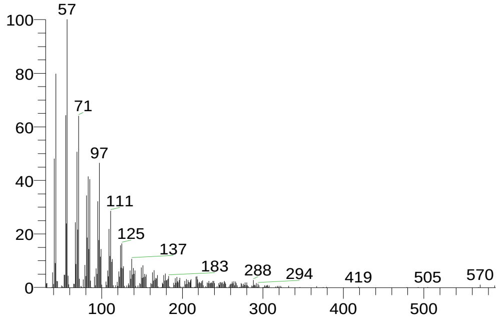

14-á-H-PREGNA
Formula C21H36, MW 288, CAS# NA, Entry# 178939
14-á-PREGNA

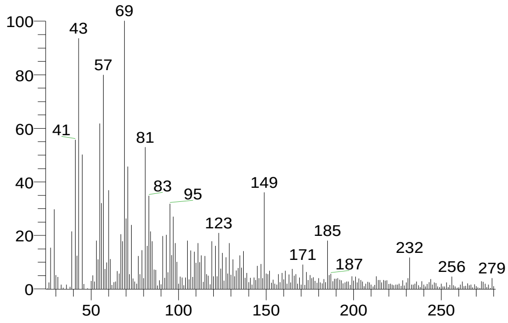

01297107001 TETRANEURIN - A - DIOL
Formula C15H20O5, MW 280, CAS# NA, Entry# 170378

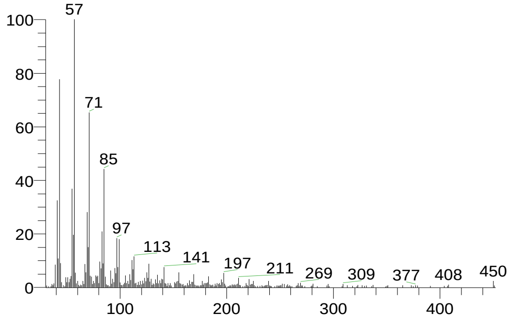

DOTRIACONTANE
Formula C32H66, MW 450, CAS# 544-85-4, Entry# 274478
AI3-52367

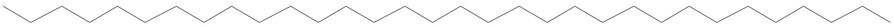

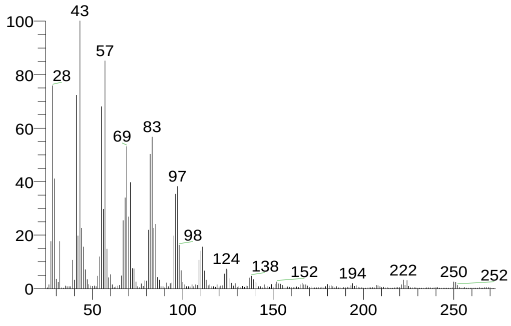

2,2-DIDEUTERO OCTADECANAL
Formula C18H34D2O, MW 270, CAS# 56555-07-8, Entry# 159359

RT	Compound Name	Molecular Weight	Molecular Formula	Cas #	Area %	Library
18.37	1-Heptatriacotanol	536	C37H76O	105794-58-9	0.22	mainlib
18.37	HEXADECADIENOIC ACID, METHYL ESTER	266	C17H30O2	29961-54-4	0.22	WileyRegistry8e
18.37	14-á-H-PREGNA	288	C21H36	NA	0.22	WileyRegistry8e
18.37	Cholestan-3-ol, 2-methylene-, (3á,5à)-	400	C28H48O	22599-96-8	0.22	mainlib

My GC-MS Report

Hit Spectrum | Compound Structure

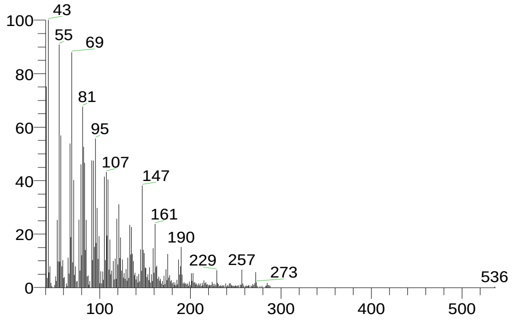

1-Heptatriacotanol
Formula C37H76O, MW 536, CAS# 105794-58-9, Entry# 6567
1-Heptatriacontanol #

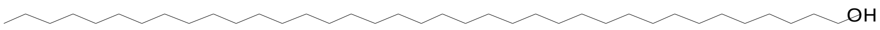

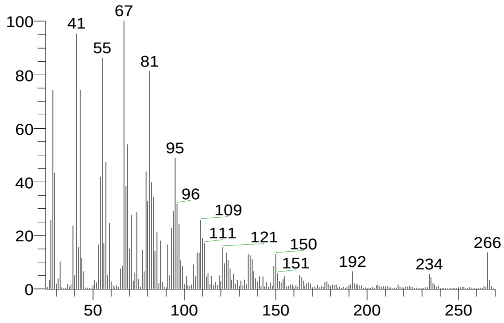

HEXADECADIENOIC ACID, METHYL ESTER
Formula C17H30O2, MW 266, CAS# 29961-54-4, Entry# 157129
METHYL HEXADECADIENOATE

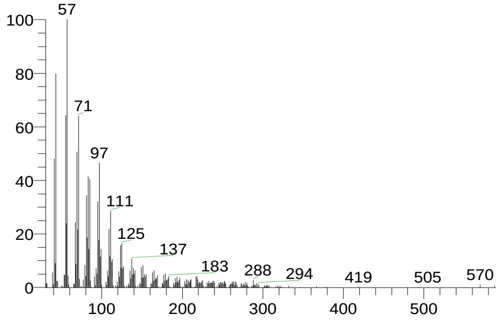

14-á-H-PREGNA
Formula C21H36, MW 288, CAS# NA, Entry# 178939
14-á-PREGNA

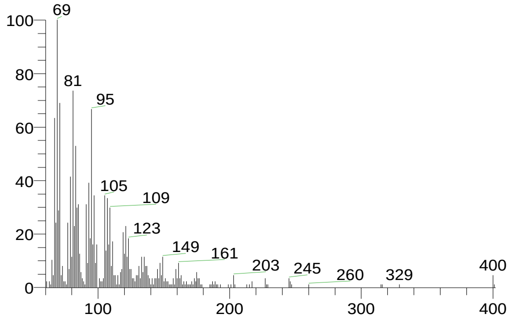

Cholestan-3-ol, 2-methylene-, (3á,5à)-
Formula C28H48O, MW 400, CAS# 22599-96-8, Entry# 28998
5à-Cholestan-3á-ol, 2-methylene-

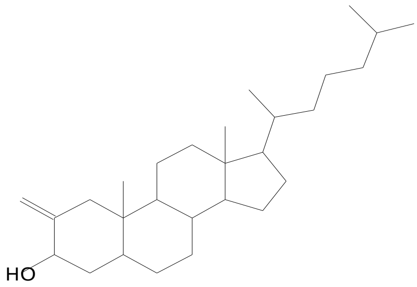

RT	Compound Name	Molecular Weight	Molecular Formula	Cas #	Area %	Library
18.59	Cholestan-3-ol, 2-methylene-, (3á,5à)-	400	C28H48O	22599-96-8	0.55	mainlib
18.59	CHOLESTAN-3-OL, 2-METHYLENE-, (3á,5à)-	400	C28H48O	22599-96-8	0.55	WileyRegistry8e
18.59	1-Heptatriacotanol	536	C37H76O	105794-58-9	0.55	mainlib
18.59	14-á-H-PREGNA	288	C21H36	NA	0.55	WileyRegistry8e

My GC-MS Report

Hit Spectrum | Compound Structure

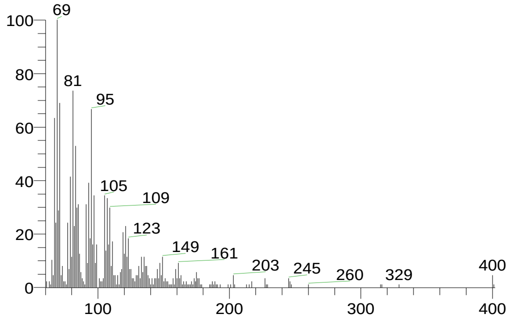

Cholestan-3-ol, 2-methylene-, (3á,5à)-
Formula C28H48O, MW 400, CAS# 22599-96-8, Entry# 28998
5à-Cholestan-3á-ol, 2-methylene-

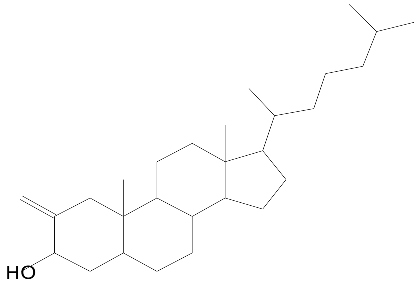

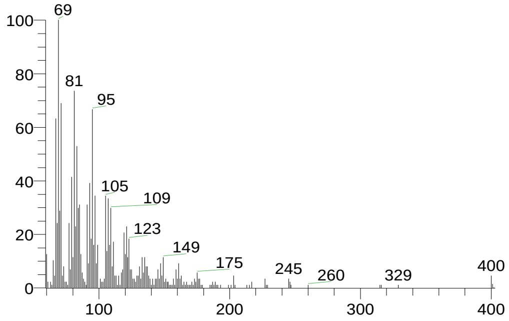

CHOLESTAN-3-OL, 2-METHYLENE-, (3á,5à)-
Formula C28H48O, MW 400, CAS# 22599-96-8, Entry# 256365
2-METHYLENECHOLESTAN-3-OL #

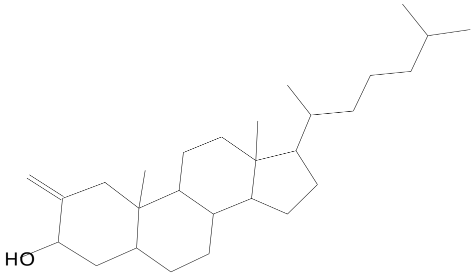

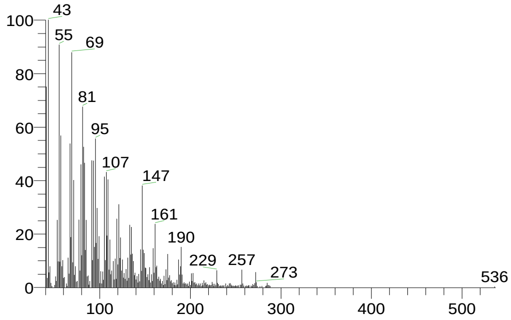

1-Heptatriacotanol
Formula C37H76O, MW 536, CAS# 105794-58-9, Entry# 6567
1-Heptatriacontanol #

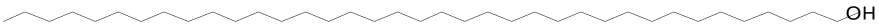

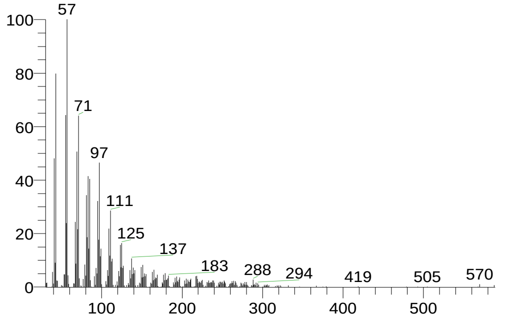

14-á-H-PREGNA
Formula C21H36, MW 288, CAS# NA, Entry# 178939
14-á-PREGNA

RT	Compound Name	Molecular Weight	Molecular Formula	Cas #	Area %	Library
19.22	DOTRIACONTANE	450	C32H66	544-85-4	0.31	WileyRegistry8e
19.22	14-á-H-PREGNA	288	C21H36	NA	0.31	WileyRegistry8e
19.22	14-á-H-PREGNA	288	C21H36	NA	0.31	WileyRegistry8e
19.22	2,2-DIDEUTERO OCTADECANAL	270	C18H34D2O	56555-07-8	0.31	WileyRegistry8e

My GC-MS Report

Hit Spectrum | Compound Structure

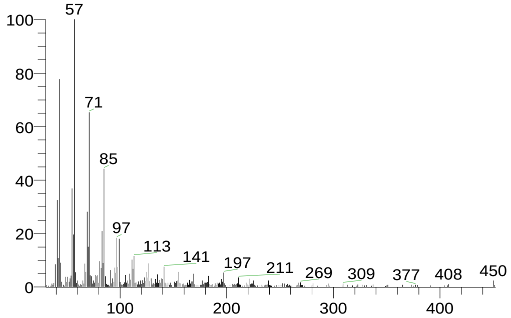

DOTRIACONTANE
Formula C32H66, MW 450, CAS# 544-85-4, Entry# 274478
AI3-52367

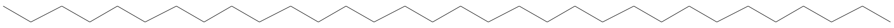

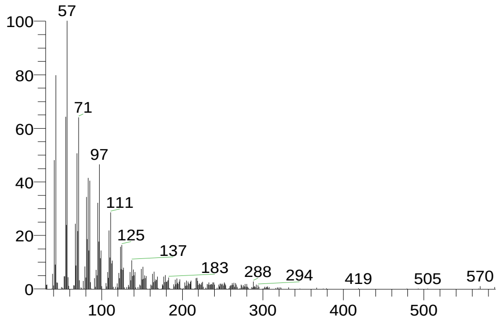

14-á-H-PREGNA
Formula C21H36, MW 288, CAS# NA, Entry# 178939
14-á-PREGNA

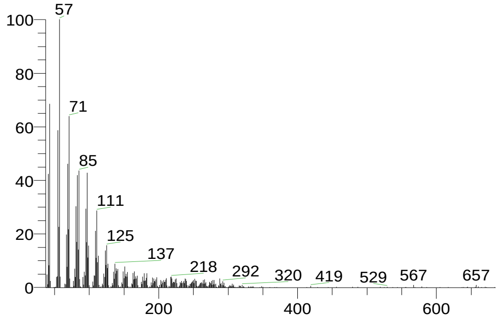

14-á-H-PREGNA
Formula C21H36, MW 288, CAS# NA, Entry# 178938
14-á-PREGNA

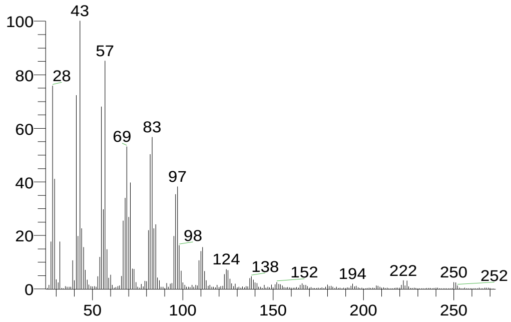

2,2-DIDEUTERO OCTADECANAL
Formula C18H34D2O, MW 270, CAS# 56555-07-8, Entry# 159359

RT	Compound Name	Molecular Weight	Molecular Formula	Cas #	Area %	Library
19.46	DOTRIACONTANE	450	C32H66	544-85-4	0.91	WileyRegistry8e
19.46	14-á-H-PREGNA	288	C21H36	NA	0.91	WileyRegistry8e
19.46	2,2-DIDEUTERO OCTADECANAL	270	C18H34D2O	56555-07-8	0.91	WileyRegistry8e
19.46	1-HEXADECANOL, 2-METHYL-	256	C17H36O	2490-48-4	0.91	WileyRegistry8e

My GC-MS Report

Hit Spectrum | Compound Structure

DOTRIACONTANE
Formula C32H66, MW 450, CAS# 544-85-4, Entry# 274478
AI3-52367

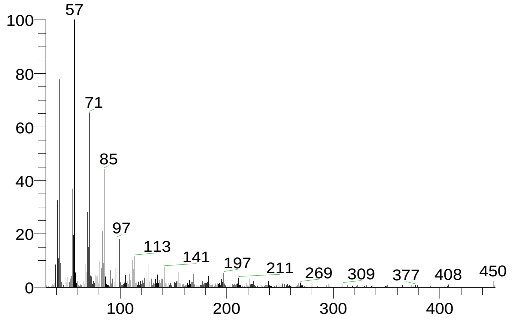

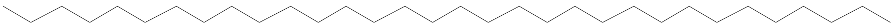

14-á-H-PREGNA
Formula C21H36, MW 288, CAS# NA, Entry# 178939
14-á-PREGNA

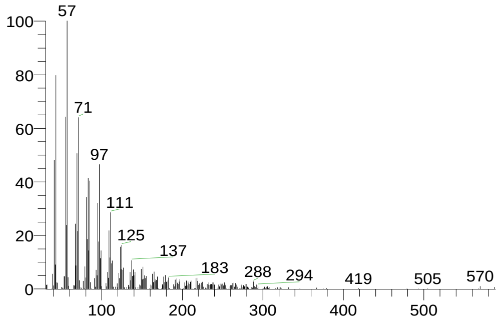

2,2-DIDEUTERO OCTADECANAL
Formula C18H34D2O, MW 270, CAS# 56555-07-8, Entry# 159359

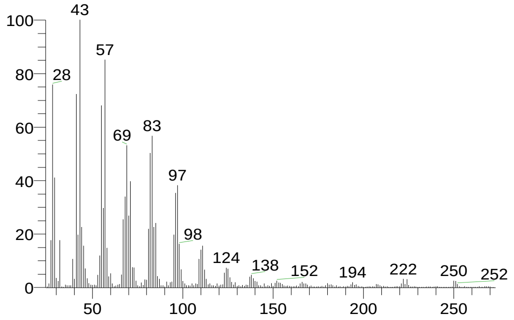

1-HEXADECANOL, 2-METHYL-
Formula C17H36O, MW 256, CAS# 2490-48-4, Entry# 146911
2-METHYLHEXADECAN-1-OL

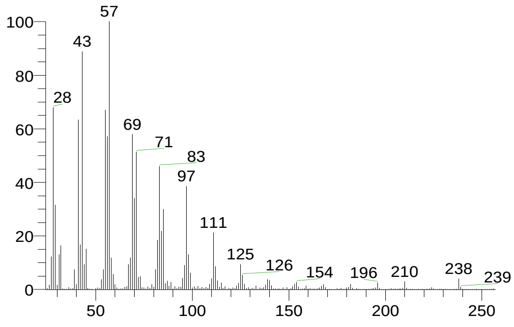

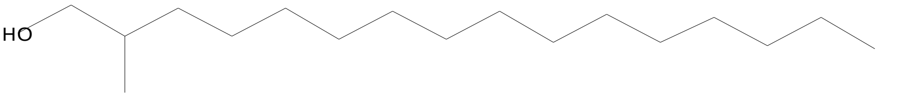

RT	Compound Name	Molecular Weight	Molecular Formula	Cas #	Area %	Library
19.70	2,2-DIDEUTERO OCTADECANAL	270	C18H34D2O	56555-07-8	0.49	WileyRegistry8e
19.70	1-HEXADECANOL, 2-METHYL-	256	C17H36O	2490-48-4	0.49	WileyRegistry8e
19.70	1-Hexadecanol, 2-methyl-	256	C17H36O	2490-48-4	0.49	mainlib
19.70	DOTRIACONTANE	450	C32H66	544-85-4	0.49	WileyRegistry8e

My GC-MS Report

Hit Spectrum | Compound Structure

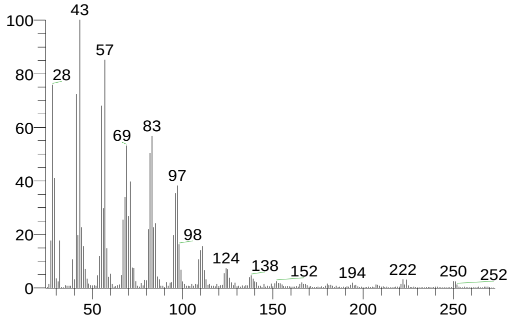

2,2-DIDEUTERO OCTADECANAL
Formula C18H34D2O, MW 270, CAS# 56555-07-8, Entry# 159359

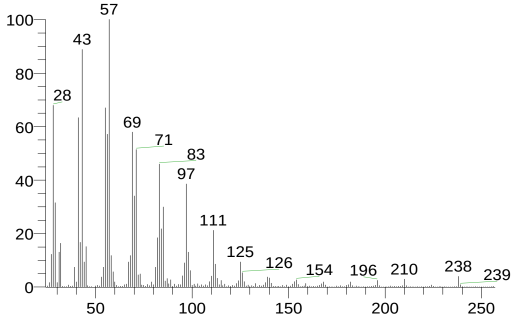

1-HEXADECANOL, 2-METHYL-
Formula C17H36O, MW 256, CAS# 2490-48-4, Entry# 146911
2-METHYLHEXADECAN-1-OL

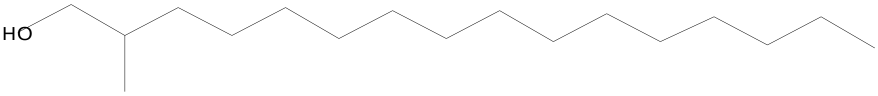

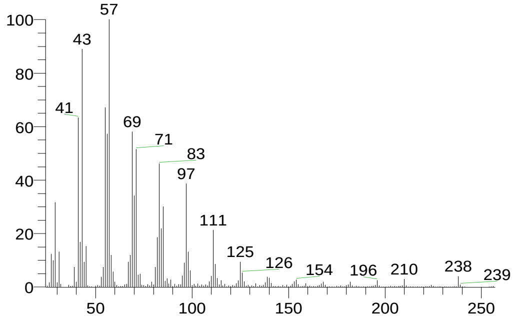

1-Hexadecanol, 2-methyl-
Formula C17H36O, MW 256, CAS# 2490-48-4, Entry# 20717
2-Methylhexadecan-1-ol

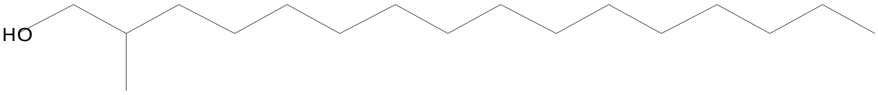

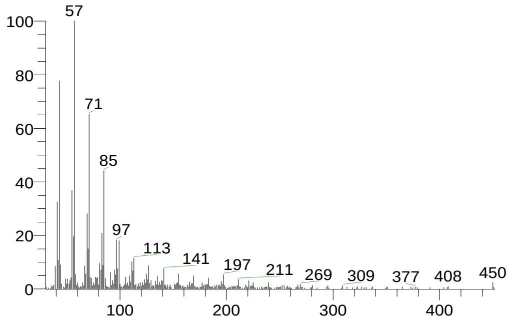

DOTRIACONTANE
Formula C32H66, MW 450, CAS# 544-85-4, Entry# 274478
AI3-52367

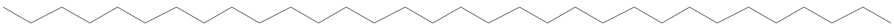

RT	Compound Name	Molecular Weight	Molecular Formula	Cas #	Area %	Library
20.37	14-á-H-PREGNA	288	C21H36	NA	0.09	WileyRegistry8e
20.37	DOTRIACONTANE	450	C32H66	544-85-4	0.09	WileyRegistry8e
20.37	1-Hexadecanol, 2-methyl-	256	C17H36O	2490-48-4	0.09	mainlib
20.37	1-HEXADECANOL, 2-METHYL-	256	C17H36O	2490-48-4	0.09	WileyRegistry8e

My GC-MS Report

Hit Spectrum | Compound Structure

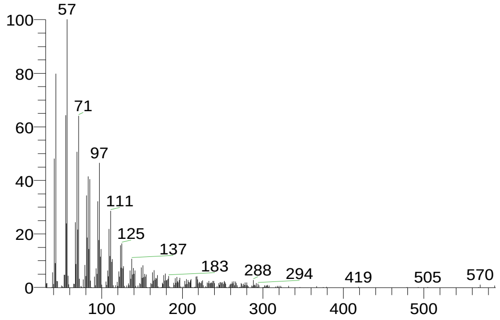

14-á-H-PREGNA
Formula C21H36, MW 288, CAS# NA, Entry# 178939
14-á-PREGNA

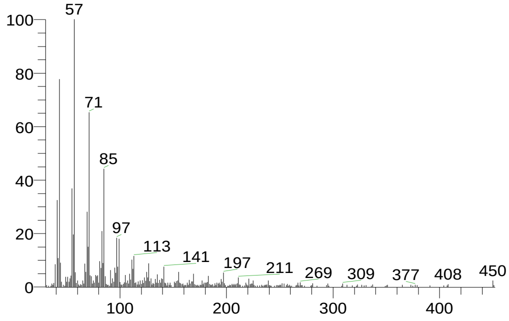

DOTRIACONTANE
Formula C32H66, MW 450, CAS# 544-85-4, Entry# 274478
AI3-52367

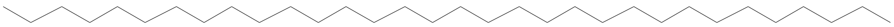

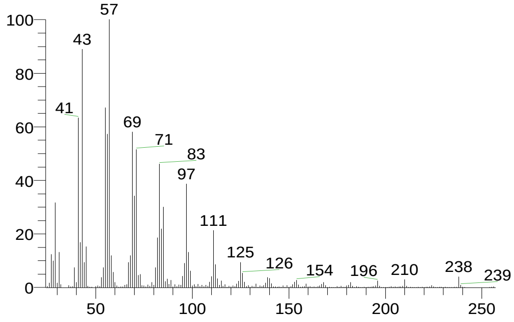

1-Hexadecanol, 2-methyl-
Formula C17H36O, MW 256, CAS# 2490-48-4, Entry# 20717
2-Methylhexadecan-1-ol

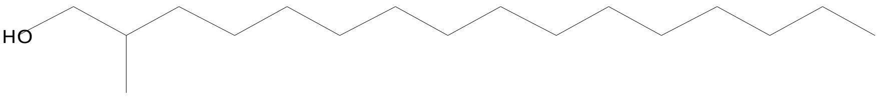

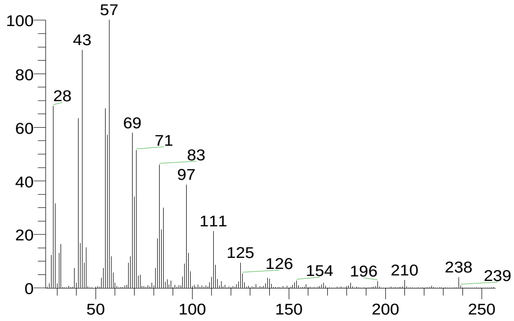

1-HEXADECANOL, 2-METHYL-
Formula C17H36O, MW 256, CAS# 2490-48-4, Entry# 146911
2-METHYLHEXADECAN-1-OL

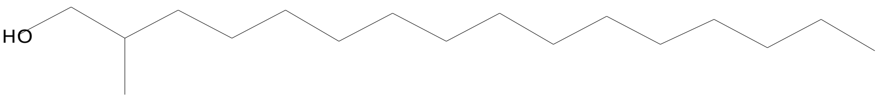

RT	Compound Name	Molecular Weight	Molecular Formula	Cas #	Area %	Library
20.57	DOTRIACONTANE	450	C32H66	544-85-4	1.20	WileyRegistry8e
20.57	1-CHLOROOCTADECANE	288	C18H37Cl	3386-33-2	1.20	WileyRegistry8e
20.57	DOCOSANE	310	C22H46	629-97-0	1.20	WileyRegistry8e
20.57	1-HEXADECANOL, 2-METHYL-	256	C17H36O	2490-48-4	1.20	WileyRegistry8e

My GC-MS Report

Hit Spectrum | Compound Structure

DOTRIACONTANE
Formula C32H66, MW 450, CAS# 544-85-4, Entry# 274478
AI3-52367

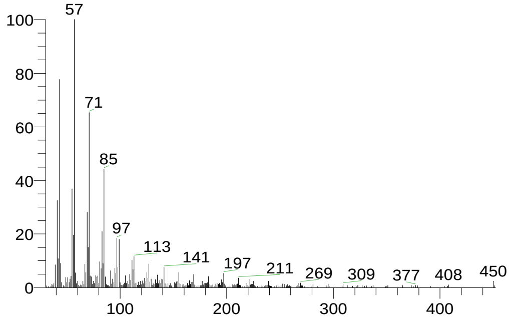

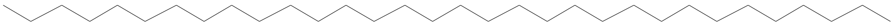

1-CHLOROOCTADECANE
Formula C18H37Cl, MW 288, CAS# 3386-33-2, Entry# 359171
1-CHLOROCTADECAN

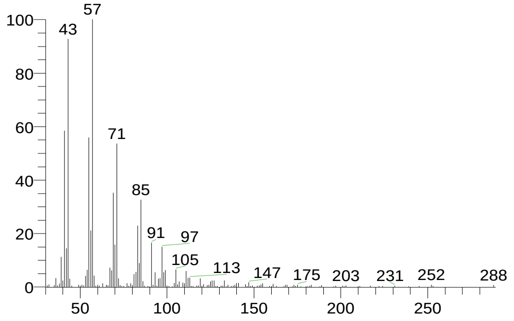

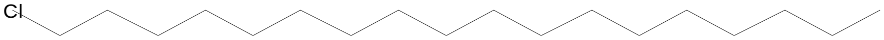

DOCOSANE
Formula C22H46, MW 310, CAS# 629-97-0, Entry# 198706
C22H46 STANDARD

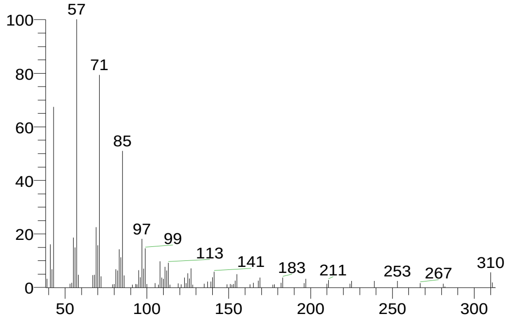

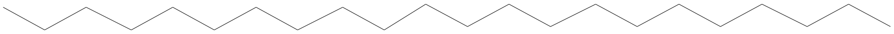

1-HEXADECANOL, 2-METHYL-
Formula C17H36O, MW 256, CAS# 2490-48-4, Entry# 146911
2-METHYLHEXADECAN-1-OL

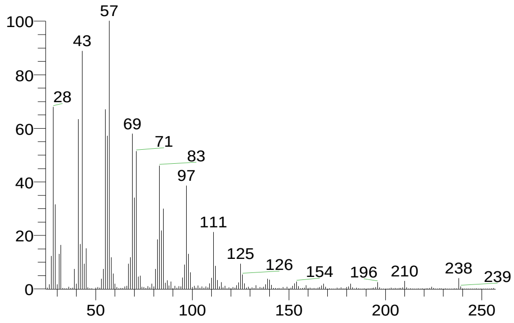

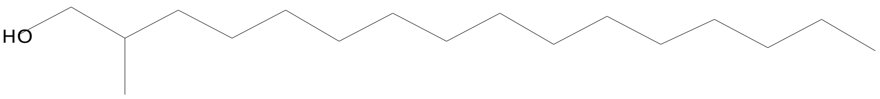

RT	Compound Name	Molecular Weight	Molecular Formula	Cas #	Area %	Library
20.80	DOCOSANE	310	C22H46	629-97-0	1.57	WileyRegistry8e
20.80	1-CHLOROOCTADECANE	288	C18H37Cl	3386-33-2	1.57	WileyRegistry8e
20.80	1-Hexadecanol, 2-methyl-	256	C17H36O	2490-48-4	1.57	mainlib
20.80	HENEICOSANE	296	C21H44	629-94-7	1.57	WileyRegistry8e

My GC-MS Report

Hit Spectrum | Compound Structure

DOCOSANE
Formula C22H46, MW 310, CAS# 629-97-0, Entry# 198707
C22H46 STANDARD

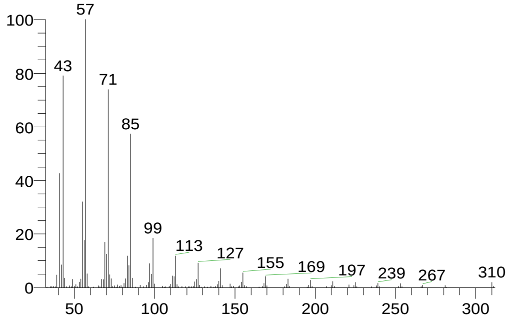

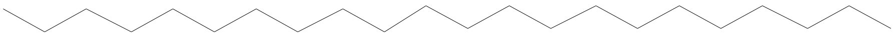

1-CHLOROOCTADECANE
Formula C18H37Cl, MW 288, CAS# 3386-33-2, Entry# 359171
1-CHLOROCTADECAN

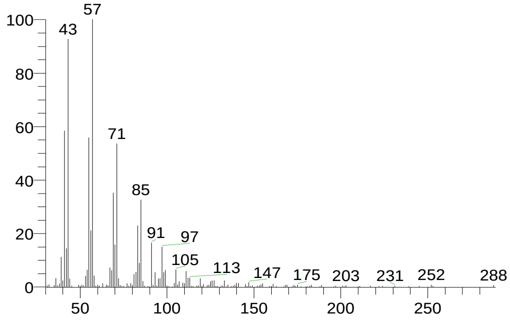

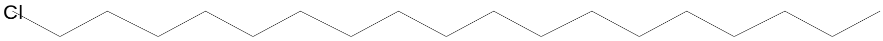

1-Hexadecanol, 2-methyl-
Formula C17H36O, MW 256, CAS# 2490-48-4, Entry# 20717
2-Methylhexadecan-1-ol

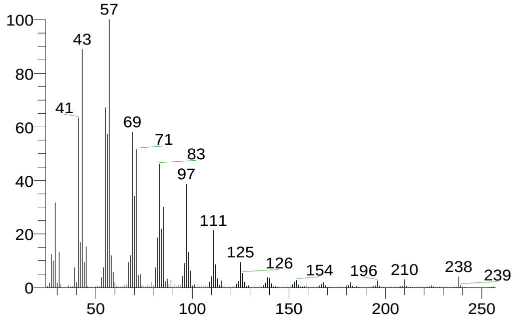

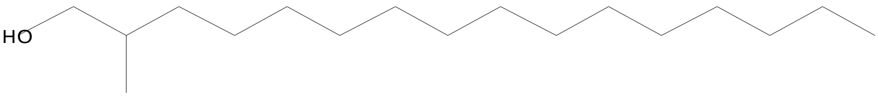

HENEICOSANE
Formula C21H44, MW 296, CAS# 629-94-7, Entry# 186361
HENICOSANE

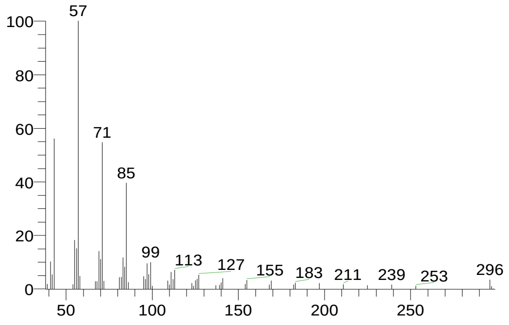

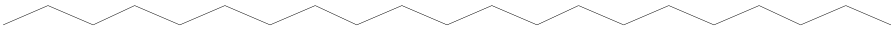

RT	Compound Name	Molecular Weight	Molecular Formula	Cas #	Area %	Library
21.00	DOTRIACONTANE	450	C32H66	544-85-4	1.86	WileyRegistry8e
21.00	1-CHLOROOCTADECANE	288	C18H37Cl	3386-33-2	1.86	WileyRegistry8e
21.00	14-á-H-PREGNA	288	C21H36	NA	1.86	WileyRegistry8e
21.00	1-Chloroeicosane	316	C20H41Cl	42217-02-7	1.86	mainlib

My GC-MS Report

Hit Spectrum | Compound Structure

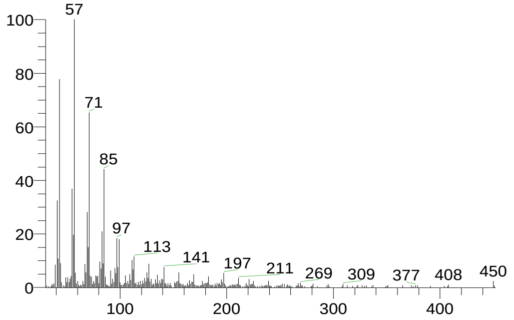

DOTRIACONTANE
Formula C32H66, MW 450, CAS# 544-85-4, Entry# 274478
AI3-52367

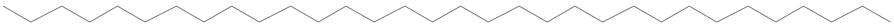

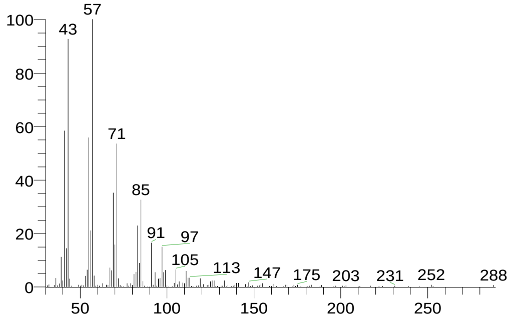

1-CHLOROOCTADECANE
Formula C18H37Cl, MW 288, CAS# 3386-33-2, Entry# 359171
1-CHLOROCTADECAN

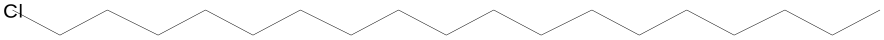

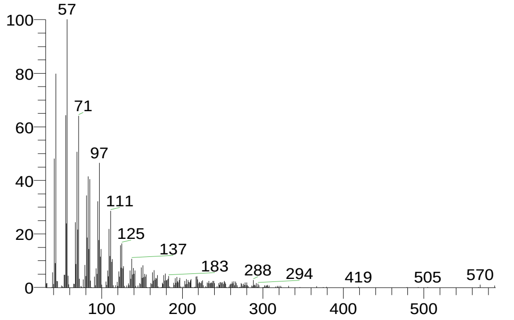

14-á-H-PREGNA
Formula C21H36, MW 288, CAS# NA, Entry# 178939
14-á-PREGNA

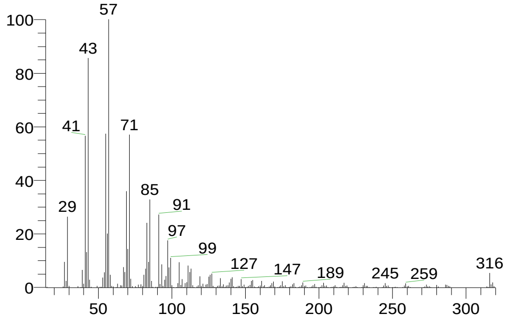

1-Chloroeicosane
Formula C20H41Cl, MW 316, CAS# 42217-02-7, Entry# 20712
Eicosane, 1-chloro-

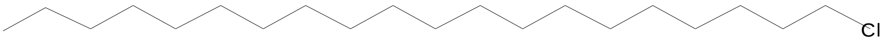

RT	Compound Name	Molecular Weight	Molecular Formula	Cas #	Area %	Library
21.30	14-á-H-PREGNA	288	C21H36	NA	0.64	WileyRegistry8e
21.30	DOTRIACONTANE	450	C32H66	544-85-4	0.64	WileyRegistry8e
21.30	14-á-H-PREGNA	288	C21H36	NA	0.64	WileyRegistry8e
21.30	2,2-DIDEUTERO OCTADECANAL	270	C18H34D2O	56555-07-8	0.64	WileyRegistry8e

My GC-MS Report

Hit Spectrum | Compound Structure

14-á-H-PREGNA
Formula C21H36, MW 288, CAS# NA, Entry# 178939
14-á-PREGNA

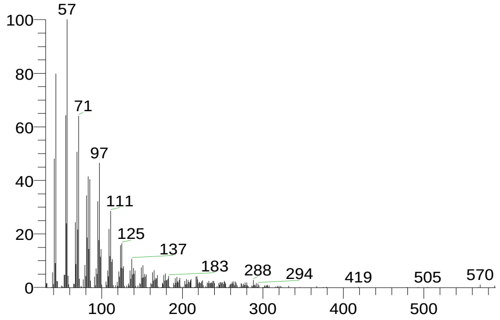

DOTRIACONTANE
Formula C32H66, MW 450, CAS# 544-85-4, Entry# 274478
AI3-52367

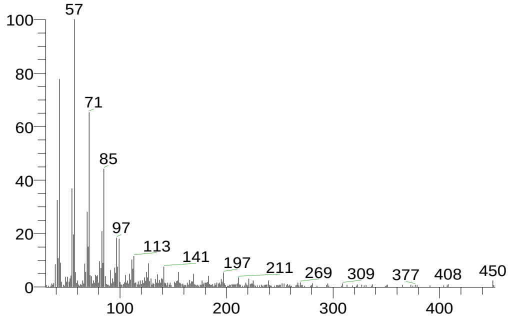

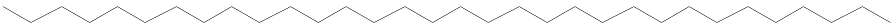

14-á-H-PREGNA
Formula C21H36, MW 288, CAS# NA, Entry# 178938
14-á-PREGNA

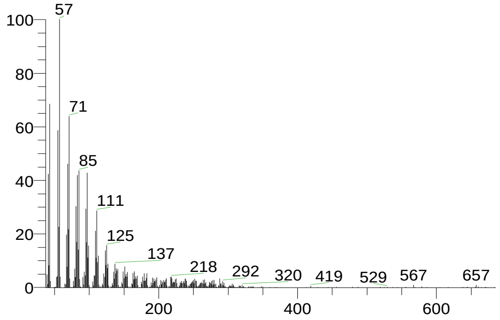

2,2-DIDEUTERO OCTADECANAL
Formula C18H34D2O, MW 270, CAS# 56555-07-8, Entry# 159359

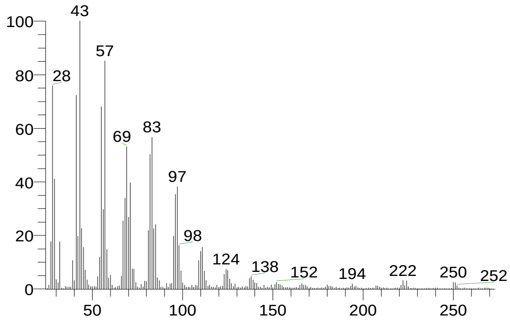

RT	Compound Name	Molecular Weight	Molecular Formula	Cas #	Area %	Library
21.41	14-á-H-PREGNA	288	C21H36	NA	0.10	WileyRegistry8e
21.41	DOTRIACONTANE	450	C32H66	544-85-4	0.10	WileyRegistry8e
21.41	14-á-H-PREGNA	288	C21H36	NA	0.10	WileyRegistry8e
21.41	2,2-DIDEUTERO OCTADECANAL	270	C18H34D2O	56555-07-8	0.10	WileyRegistry8e

My GC-MS Report

Hit Spectrum | Compound Structure

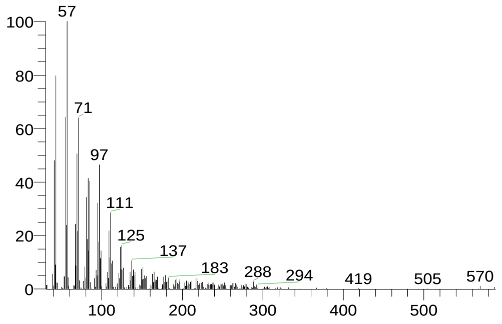

14-á-H-PREGNA
Formula C21H36, MW 288, CAS# NA, Entry# 178939
14-á-PREGNA

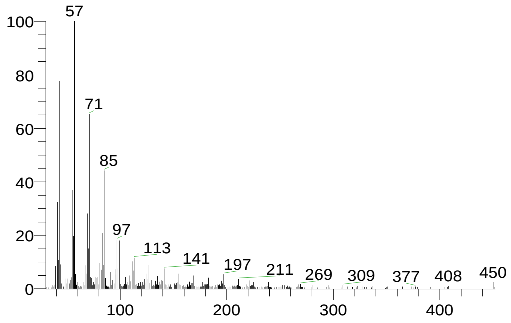

DOTRIACONTANE
Formula C32H66, MW 450, CAS# 544-85-4, Entry# 274478
AI3-52367

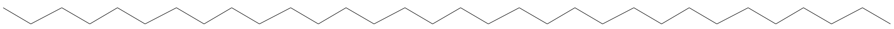

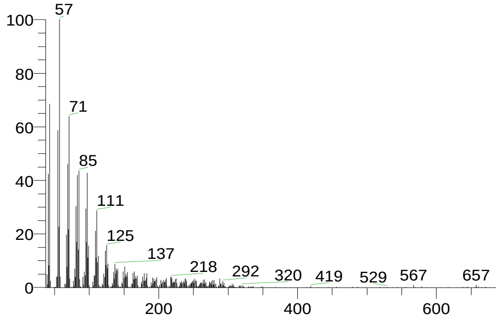

14-á-H-PREGNA
Formula C21H36, MW 288, CAS# NA, Entry# 178938
14-á-PREGNA

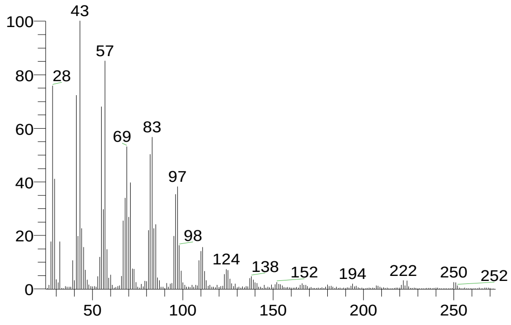

2,2-DIDEUTERO OCTADECANAL
Formula C18H34D2O, MW 270, CAS# 56555-07-8, Entry# 159359

RT	Compound Name	Molecular Weight	Molecular Formula	Cas #	Area %	Library
21.78	14-á-H-PREGNA	288	C21H36	NA	2.24	WileyRegistry8e
21.78	DOTRIACONTANE	450	C32H66	544-85-4	2.24	WileyRegistry8e
21.78	14-á-H-PREGNA	288	C21H36	NA	2.24	WileyRegistry8e
21.78	2,2-DIDEUTERO OCTADECANAL	270	C18H34D2O	56555-07-8	2.24	WileyRegistry8e

My GC-MS Report

Hit Spectrum | Compound Structure

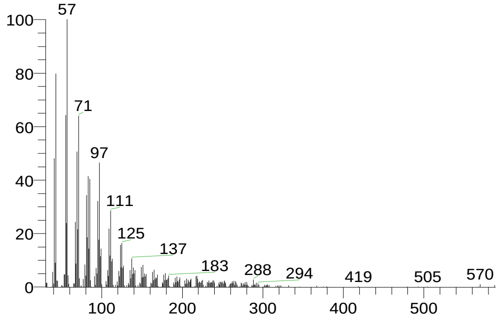

14-á-H-PREGNA
Formula C21H36, MW 288, CAS# NA, Entry# 178939
14-á-PREGNA

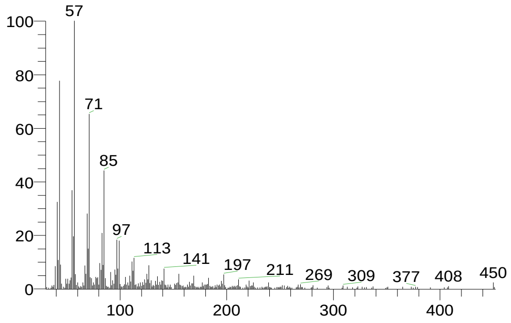

DOTRIACONTANE
Formula C32H66, MW 450, CAS# 544-85-4, Entry# 274478
AI3-52367

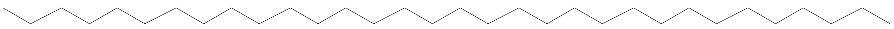

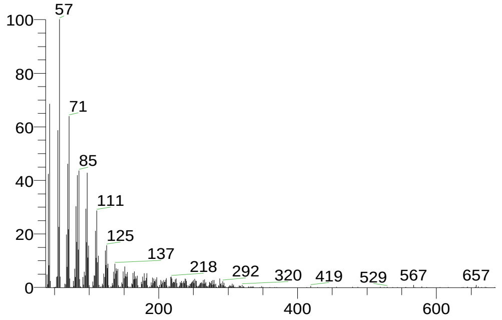

14-á-H-PREGNA
Formula C21H36, MW 288, CAS# NA, Entry# 178938
14-á-PREGNA

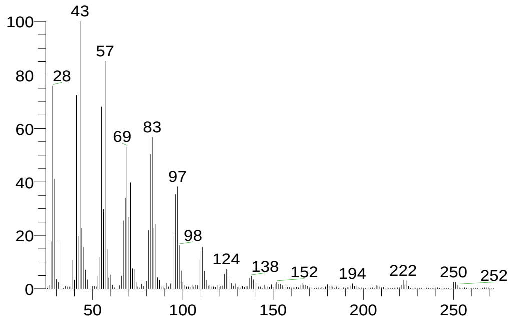

2,2-DIDEUTERO OCTADECANAL
Formula C18H34D2O, MW 270, CAS# 56555-07-8, Entry# 159359

RT	Compound Name	Molecular Weight	Molecular Formula	Cas #	Area %	Library
21.90	DOTRIACONTANE	450	C32H66	544-85-4	1.86	WileyRegistry8e
21.90	14-á-H-PREGNA	288	C21H36	NA	1.86	WileyRegistry8e
21.90	1-Hexadecanol, 2-methyl-	256	C17H36O	2490-48-4	1.86	mainlib
21.90	1-HEXADECANOL, 2-METHYL-	256	C17H36O	2490-48-4	1.86	WileyRegistry8e

My GC-MS Report

Hit Spectrum | Compound Structure

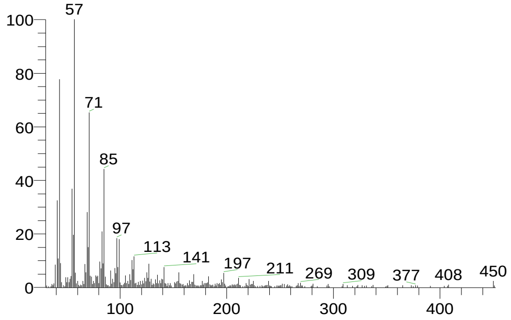

DOTRIACONTANE
Formula C32H66, MW 450, CAS# 544-85-4, Entry# 274478
AI3-52367

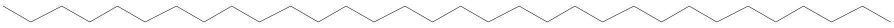

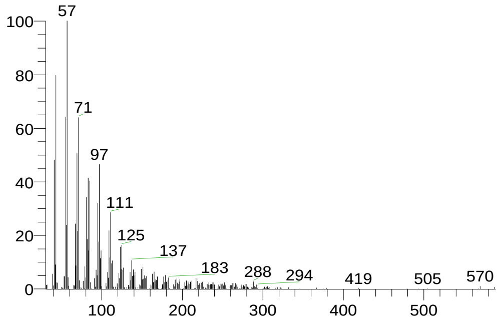

14-á-H-PREGNA
Formula C21H36, MW 288, CAS# NA, Entry# 178939
14-á-PREGNA

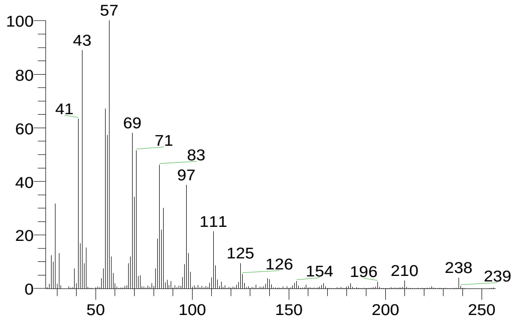

1-Hexadecanol, 2-methyl-
Formula C17H36O, MW 256, CAS# 2490-48-4, Entry# 20717
2-Methylhexadecan-1-ol

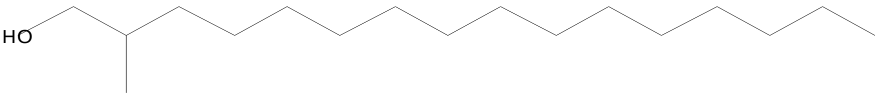

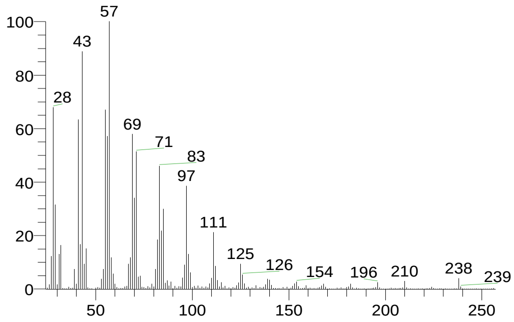

1-HEXADECANOL, 2-METHYL-
Formula C17H36O, MW 256, CAS# 2490-48-4, Entry# 146911
2-METHYLHEXADECAN-1-OL

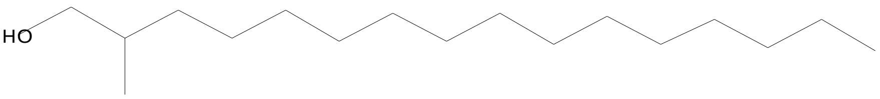

RT	Compound Name	Molecular Weight	Molecular Formula	Cas #	Area %	Library
22.29	2,2-DIDEUTERO OCTADECANAL	270	C18H34D2O	56555-07-8	0.15	WileyRegistry8e
22.29	14-á-H-PREGNA	288	C21H36	NA	0.15	WileyRegistry8e
22.29	14-á-H-PREGNA	288	C21H36	NA	0.15	WileyRegistry8e
22.29	2-Dodecen-1-yl(-)succinic anhydride	266	C16H26O3	19780-11-1	0.15	mainlib

My GC-MS Report

Hit Spectrum | Compound Structure

2,2-DIDEUTERO OCTADECANAL
Formula C18H34D2O, MW 270, CAS# 56555-07-8, Entry# 159359

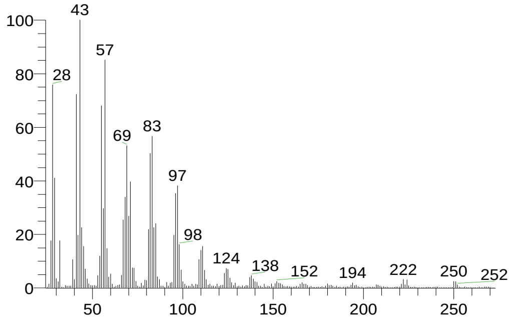

14-á-H-PREGNA
Formula C21H36, MW 288, CAS# NA, Entry# 178939
14-á-PREGNA

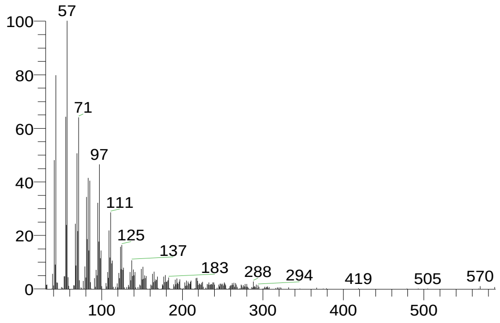

14-á-H-PREGNA
Formula C21H36, MW 288, CAS# NA, Entry# 178938
14-á-PREGNA

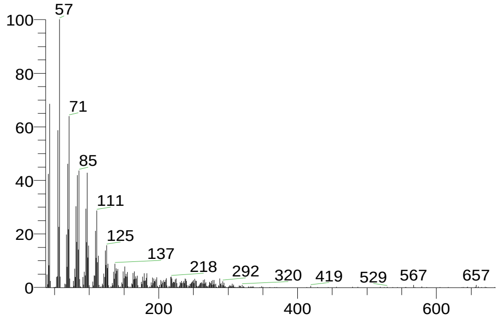

2-Dodecen-1-yl(-)succinic anhydride
Formula C16H26O3, MW 266, CAS# 19780-11-1, Entry# 2388
2,5-Furandione, 3-dodecenyl-

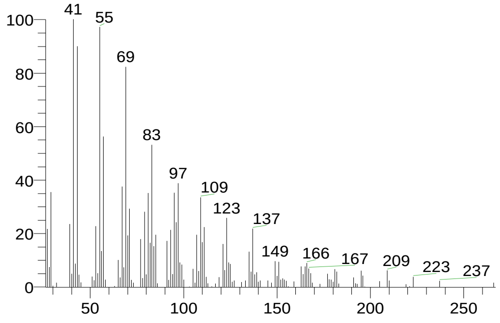

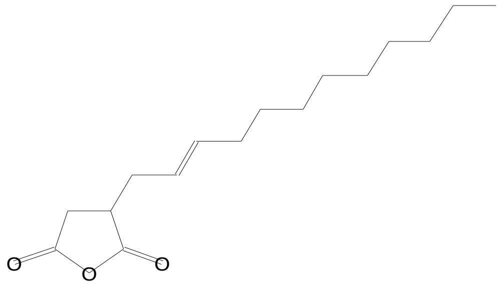

RT	Compound Name	Molecular Weight	Molecular Formula	Cas #	Area %	Library
22.46	2,2-DIDEUTERO OCTADECANAL	270	C18H34D2O	56555-07-8	1.22	WileyRegistry8e
22.46	14-á-H-PREGNA	288	C21H36	NA	1.22	WileyRegistry8e
22.46	DOTRIACONTANE	450	C32H66	544-85-4	1.22	WileyRegistry8e
22.46	14-á-H-PREGNA	288	C21H36	NA	1.22	WileyRegistry8e

My GC-MS Report

Hit Spectrum | Compound Structure

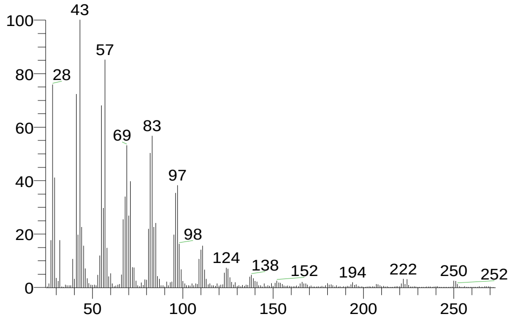

2,2-DIDEUTERO OCTADECANAL
Formula C18H34D2O, MW 270, CAS# 56555-07-8, Entry# 159359

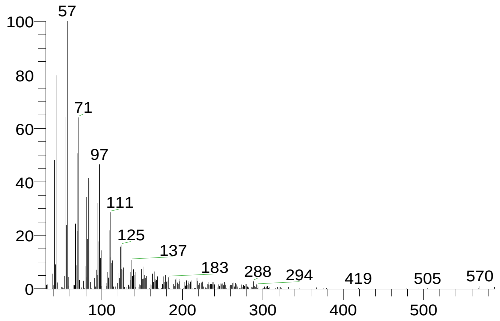

14-á-H-PREGNA
Formula C21H36, MW 288, CAS# NA, Entry# 178939
14-á-PREGNA

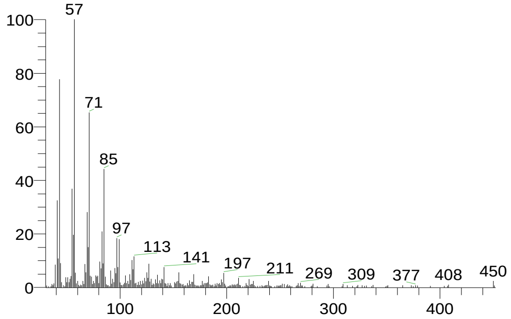

DOTRIACONTANE
Formula C32H66, MW 450, CAS# 544-85-4, Entry# 274478
AI3-52367

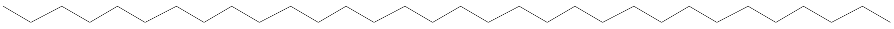

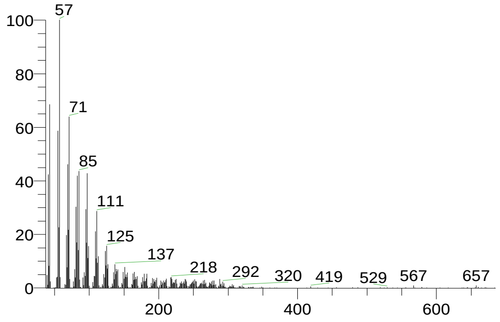

14-á-H-PREGNA
Formula C21H36, MW 288, CAS# NA, Entry# 178938
14-á-PREGNA

RT	Compound Name	Molecular Weight	Molecular Formula	Cas #	Area %	Library
22.65	DOTRIACONTANE	450	C32H66	544-85-4	2.23	WileyRegistry8e
22.65	14-á-H-PREGNA	288	C21H36	NA	2.23	WileyRegistry8e
22.65	1-Hexadecanol, 2-methyl-	256	C17H36O	2490-48-4	2.23	mainlib
22.65	1-HEXADECANOL, 2-METHYL-	256	C17H36O	2490-48-4	2.23	WileyRegistry8e

My GC-MS Report

Hit Spectrum | Compound Structure

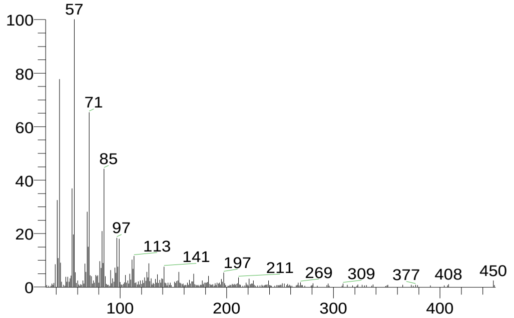

DOTRIACONTANE
Formula C32H66, MW 450, CAS# 544-85-4, Entry# 274478
AI3-52367

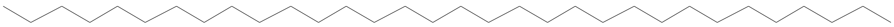

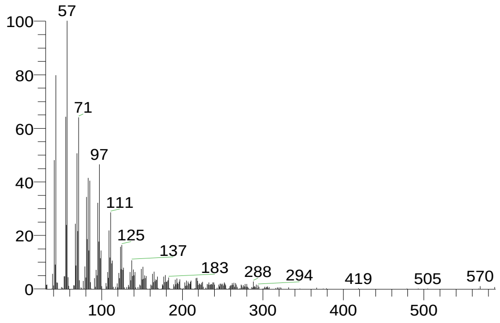

14-á-H-PREGNA
Formula C21H36, MW 288, CAS# NA, Entry# 178939
14-á-PREGNA

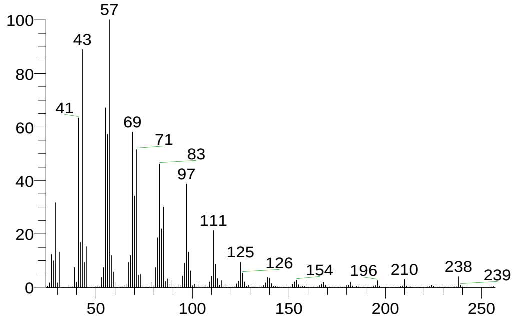

1-Hexadecanol, 2-methyl-
Formula C17H36O, MW 256, CAS# 2490-48-4, Entry# 20717
2-Methylhexadecan-1-ol

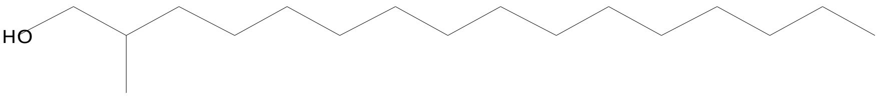

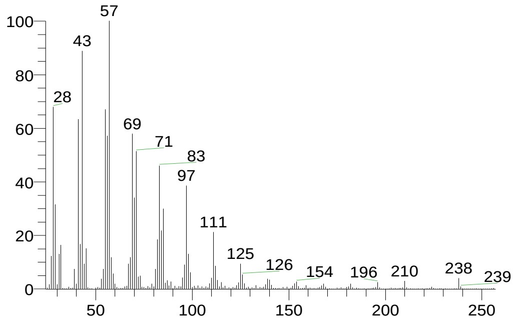

1-HEXADECANOL, 2-METHYL-
Formula C17H36O, MW 256, CAS# 2490-48-4, Entry# 146911
2-METHYLHEXADECAN-1-OL

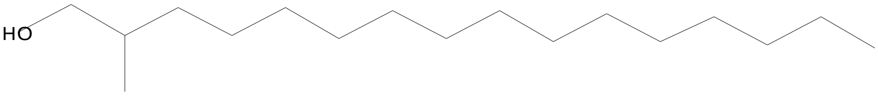

RT	Compound Name	Molecular Weight	Molecular Formula	Cas #	Area %	Library
22.78	DOTRIACONTANE	450	C32H66	544-85-4	0.28	WileyRegistry8e
22.78	14-á-H-PREGNA	288	C21H36	NA	0.28	WileyRegistry8e
22.78	2,2-DIDEUTERO OCTADECANAL	270	C18H34D2O	56555-07-8	0.28	WileyRegistry8e
22.78	1-Hexadecanol, 2-methyl-	256	C17H36O	2490-48-4	0.28	mainlib

My GC-MS Report

Hit Spectrum | Compound Structure

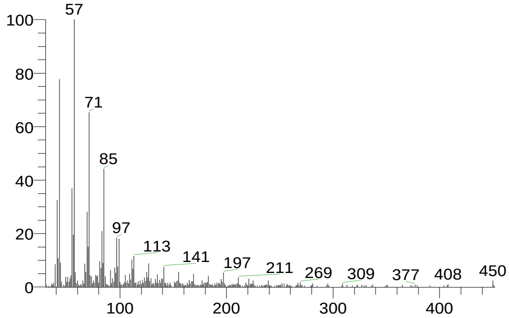

DOTRIACONTANE
Formula C32H66, MW 450, CAS# 544-85-4, Entry# 274478
AI3-52367

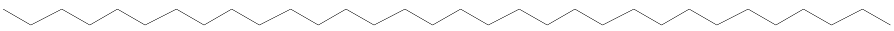

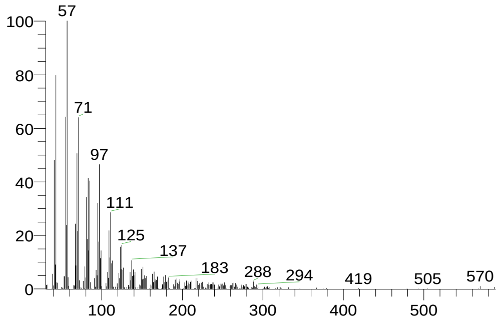

14-á-H-PREGNA
Formula C21H36, MW 288, CAS# NA, Entry# 178939
14-á-PREGNA

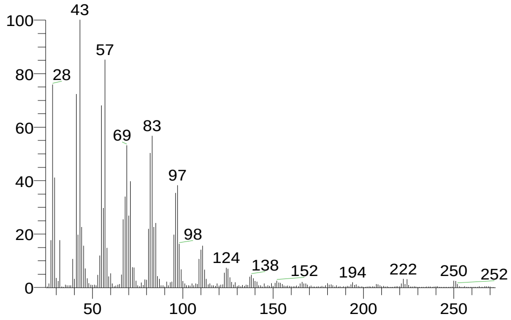

2,2-DIDEUTERO OCTADECANAL
Formula C18H34D2O, MW 270, CAS# 56555-07-8, Entry# 159359

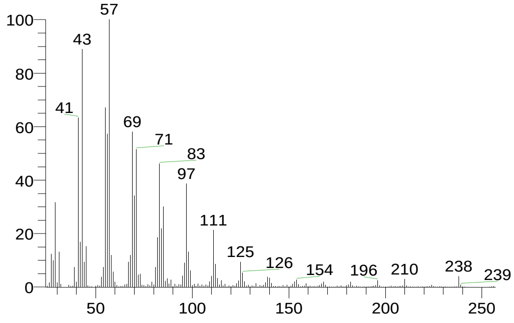

1-Hexadecanol, 2-methyl-
Formula C17H36O, MW 256, CAS# 2490-48-4, Entry# 20717
2-Methylhexadecan-1-ol

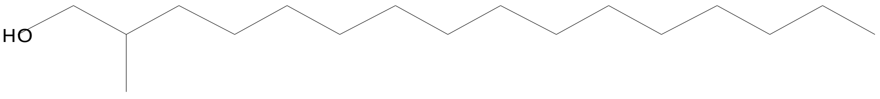

RT	Compound Name	Molecular Weight	Molecular Formula	Cas #	Area %	Library
22.97	DOTRIACONTANE	450	C32H66	544-85-4	0.49	WileyRegistry8e
22.97	14-á-H-PREGNA	288	C21H36	NA	0.49	WileyRegistry8e
22.97	2,2-DIDEUTERO OCTADECANAL	270	C18H34D2O	56555-07-8	0.49	WileyRegistry8e
22.97	14-á-H-PREGNA	288	C21H36	NA	0.49	WileyRegistry8e

My GC-MS Report

Hit Spectrum | Compound Structure

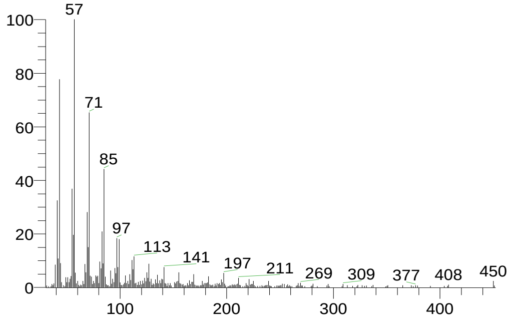

DOTRIACONTANE
Formula C32H66, MW 450, CAS# 544-85-4, Entry# 274478
AI3-52367

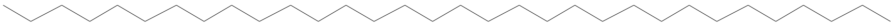

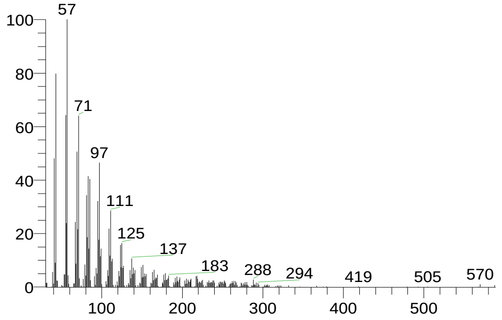

14-á-H-PREGNA
Formula C21H36, MW 288, CAS# NA, Entry# 178939
14-á-PREGNA

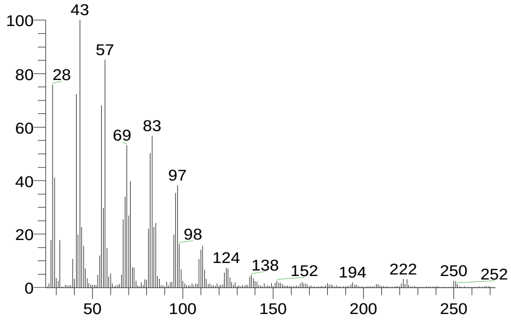

2,2-DIDEUTERO OCTADECANAL
Formula C18H34D2O, MW 270, CAS# 56555-07-8, Entry# 159359

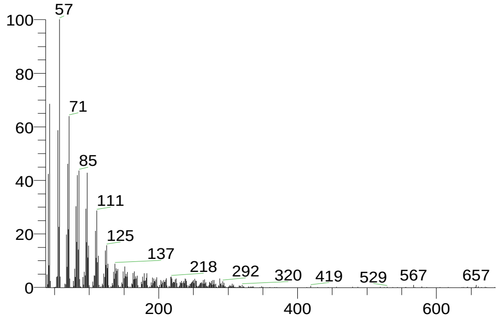

14-á-H-PREGNA
Formula C21H36, MW 288, CAS# NA, Entry# 178938
14-á-PREGNA

RT	Compound Name	Molecular Weight	Molecular Formula	Cas #	Area %	Library
23.10	DOTRIACONTANE	450	C32H66	544-85-4	1.60	WileyRegistry8e
23.10	14-á-H-PREGNA	288	C21H36	NA	1.60	WileyRegistry8e
23.10	2-OCTADECYLOXY-1,1,2,2-TETRADEUTEROETHANOL	318	C20H38D4O2	56599-39-4	1.60	WileyRegistry8e
23.10	14-á-H-PREGNA	288	C21H36	NA	1.60	WileyRegistry8e

My GC-MS Report

Hit Spectrum | Compound Structure

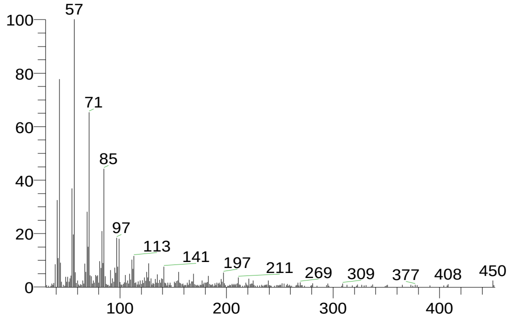

DOTRIACONTANE
Formula C32H66, MW 450, CAS# 544-85-4, Entry# 274478
AI3-52367

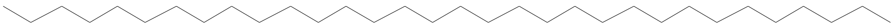

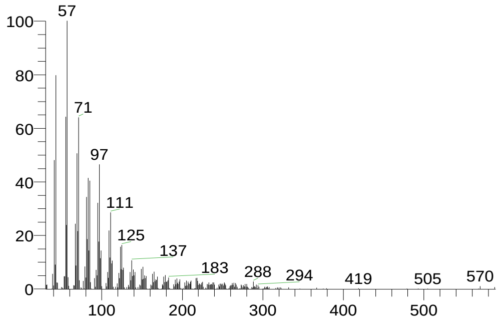

14-á-H-PREGNA
Formula C21H36, MW 288, CAS# NA, Entry# 178939
14-á-PREGNA

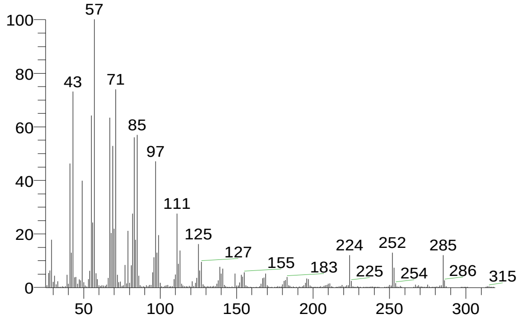

2-OCTADECYLOXY-1,1,2,2-TETRADEUTEROETHANOL
Formula C20H38D4O2, MW 318, CAS# 56599-39-4, Entry# 202042

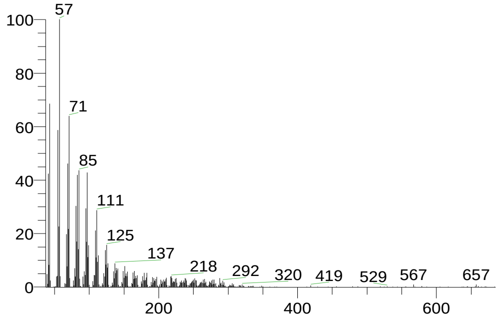

14-á-H-PREGNA
Formula C21H36, MW 288, CAS# NA, Entry# 178938
14-á-PREGNA

RT	Compound Name	Molecular Weight	Molecular Formula	Cas #	Area %	Library
23.24	DOTRIACONTANE	450	C32H66	544-85-4	0.32	WileyRegistry8e
23.24	14-á-H-PREGNA	288	C21H36	NA	0.32	WileyRegistry8e
23.24	2,2-DIDEUTERO OCTADECANAL	270	C18H34D2O	56555-07-8	0.32	WileyRegistry8e
23.24	14-á-H-PREGNA	288	C21H36	NA	0.32	WileyRegistry8e

My GC-MS Report

Hit Spectrum | Compound Structure

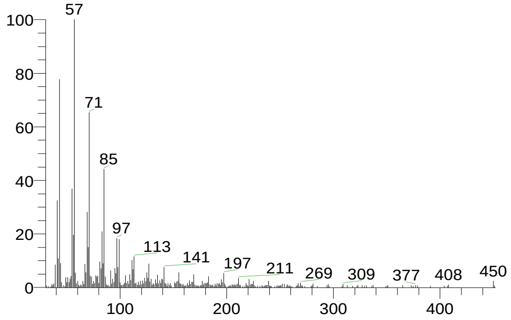

DOTRIACONTANE
Formula C32H66, MW 450, CAS# 544-85-4, Entry# 274478
AI3-52367

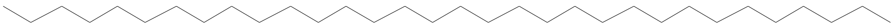

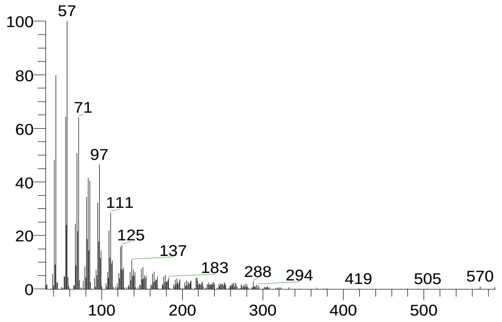

14-á-H-PREGNA
Formula C21H36, MW 288, CAS# NA, Entry# 178939
14-á-PREGNA

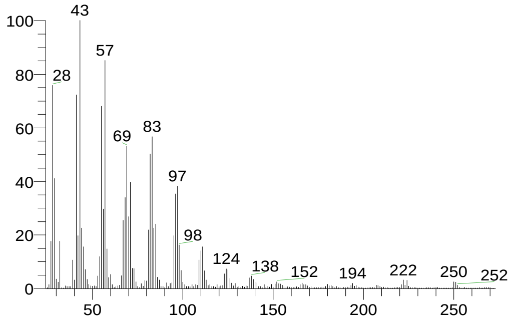

2,2-DIDEUTERO OCTADECANAL
Formula C18H34D2O, MW 270, CAS# 56555-07-8, Entry# 159359

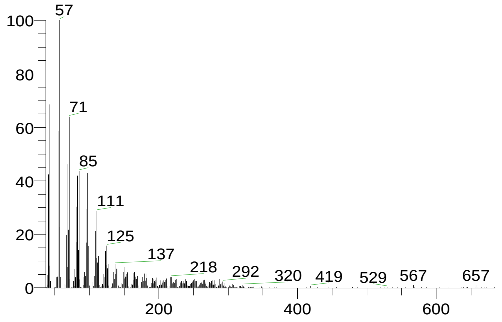

14-á-H-PREGNA
Formula C21H36, MW 288, CAS# NA, Entry# 178938
14-á-PREGNA

RT	Compound Name	Molecular Weight	Molecular Formula	Cas #	Area %	Library
23.31	DOTRIACONTANE	450	C32H66	544-85-4	0.56	WileyRegistry8e
23.31	14-á-H-PREGNA	288	C21H36	NA	0.56	WileyRegistry8e
23.31	2,2-DIDEUTERO OCTADECANAL	270	C18H34D2O	56555-07-8	0.56	WileyRegistry8e
23.31	2-OCTADECYLOXY-1,1,2,2-TETRADEUTEROETHANOL	318	C20H38D4O2	56599-39-4	0.56	WileyRegistry8e

My GC-MS Report

Hit Spectrum | Compound Structure

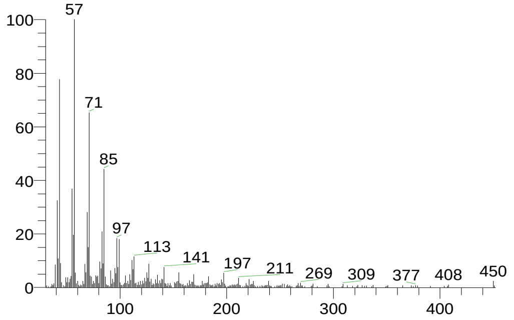

DOTRIACONTANE
Formula C32H66, MW 450, CAS# 544-85-4, Entry# 274478
AI3-52367

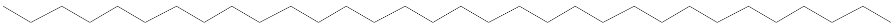

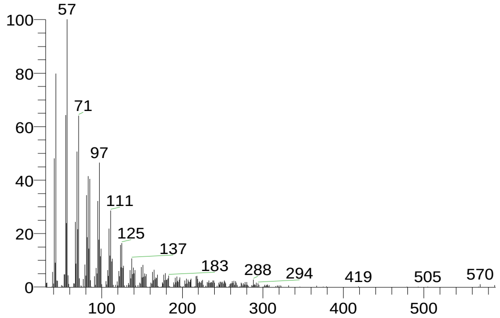

14-á-H-PREGNA
Formula C21H36, MW 288, CAS# NA, Entry# 178939
14-á-PREGNA

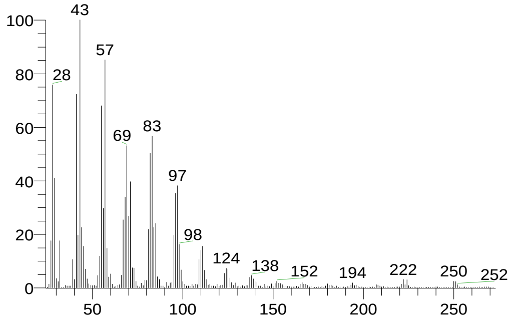

2,2-DIDEUTERO OCTADECANAL
Formula C18H34D2O, MW 270, CAS# 56555-07-8, Entry# 159359

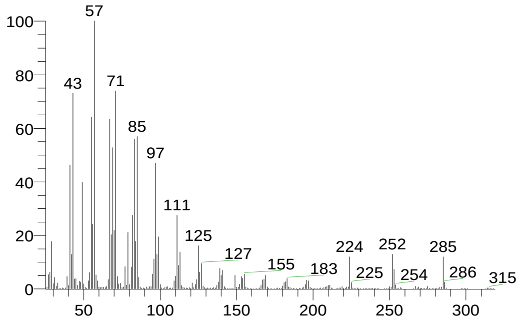

2-OCTADECYLOXY-1,1,2,2-TETRADEUTEROETHANOL
Formula C20H38D4O2, MW 318, CAS# 56599-39-4, Entry# 202042

RT	Compound Name	Molecular Weight	Molecular Formula	Cas #	Area %	Library
23.41	14-á-H-PREGNA	288	C21H36	NA	0.49	WileyRegistry8e
23.41	DOTRIACONTANE	450	C32H66	544-85-4	0.49	WileyRegistry8e
23.41	14-á-H-PREGNA	288	C21H36	NA	0.49	WileyRegistry8e
23.41	2,2-DIDEUTERO OCTADECANAL	270	C18H34D2O	56555-07-8	0.49	WileyRegistry8e

My GC-MS Report

Hit Spectrum | Compound Structure

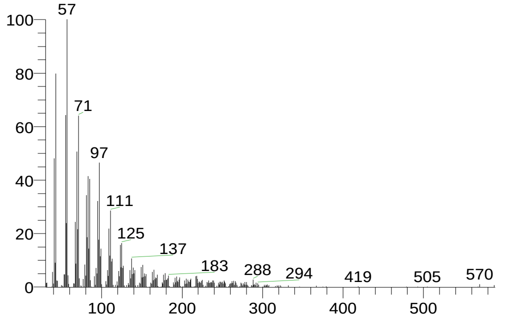

14-á-H-PREGNA
Formula C21H36, MW 288, CAS# NA, Entry# 178939
14-á-PREGNA

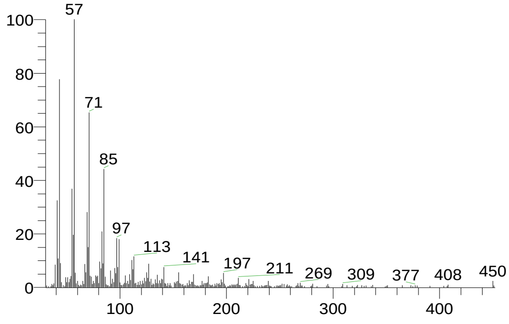

DOTRIACONTANE
Formula C32H66, MW 450, CAS# 544-85-4, Entry# 274478
AI3-52367

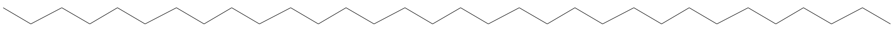

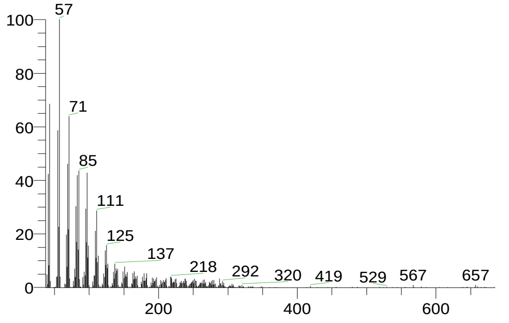

14-á-H-PREGNA
Formula C21H36, MW 288, CAS# NA, Entry# 178938
14-á-PREGNA

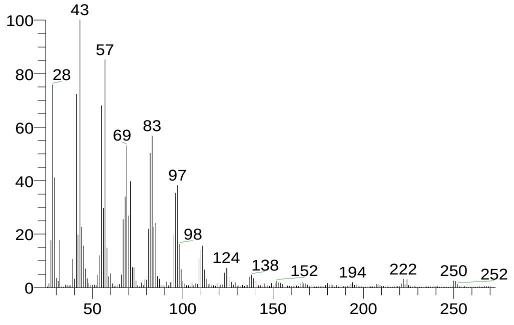

2,2-DIDEUTERO OCTADECANAL
Formula C18H34D2O, MW 270, CAS# 56555-07-8, Entry# 159359

RT	Compound Name	Molecular Weight	Molecular Formula	Cas #	Area %	Library
23.53	14-á-H-PREGNA	288	C21H36	NA	0.96	WileyRegistry8e
23.53	DOTRIACONTANE	450	C32H66	544-85-4	0.96	WileyRegistry8e
23.53	14-á-H-PREGNA	288	C21H36	NA	0.96	WileyRegistry8e
23.53	2,2-DIDEUTERO OCTADECANAL	270	C18H34D2O	56555-07-8	0.96	WileyRegistry8e

My GC-MS Report

Hit Spectrum | Compound Structure

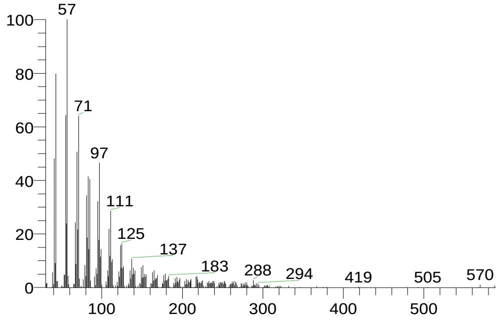

14-á-H-PREGNA
Formula C21H36, MW 288, CAS# NA, Entry# 178939
14-á-PREGNA

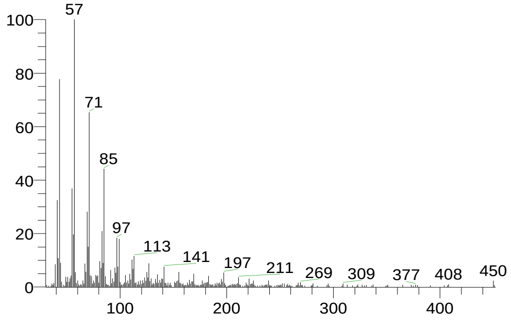

DOTRIACONTANE
Formula C32H66, MW 450, CAS# 544-85-4, Entry# 274478
AI3-52367

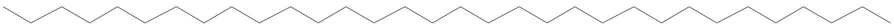

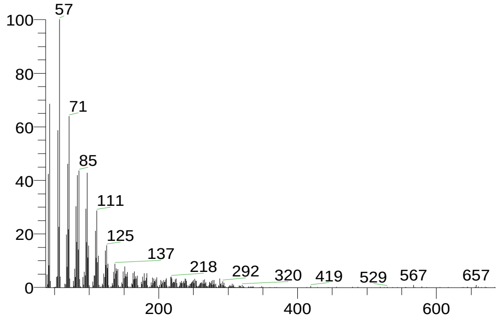

14-á-H-PREGNA
Formula C21H36, MW 288, CAS# NA, Entry# 178938
14-á-PREGNA

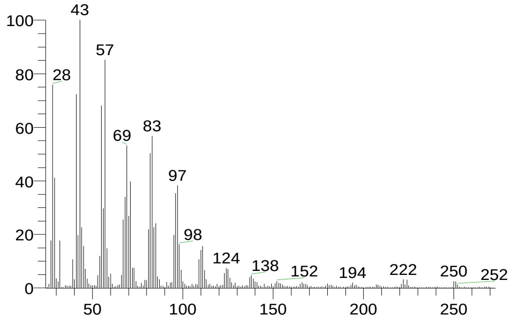

2,2-DIDEUTERO OCTADECANAL
Formula C18H34D2O, MW 270, CAS# 56555-07-8, Entry# 159359

RT	Compound Name	Molecular Weight	Molecular Formula	Cas #	Area %	Library
23.95	DOTRIACONTANE	450	C32H66	544-85-4	4.89	WileyRegistry8e
23.95	14-á-H-PREGNA	288	C21H36	NA	4.89	WileyRegistry8e
23.95	1-HEXADECANOL, 2-METHYL-	256	C17H36O	2490-48-4	4.89	WileyRegistry8e
23.95	1-Hexadecanol, 2-methyl-	256	C17H36O	2490-48-4	4.89	mainlib

My GC-MS Report

Hit Spectrum | Compound Structure

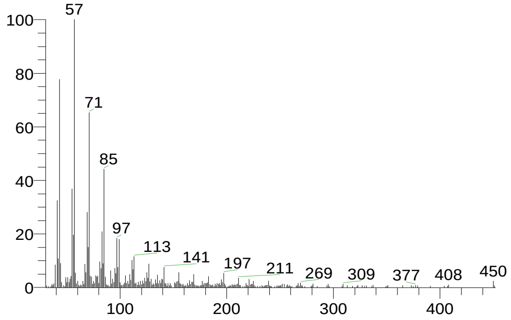

DOTRIACONTANE
Formula C32H66, MW 450, CAS# 544-85-4, Entry# 274478
AI3-52367

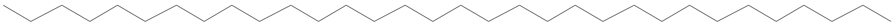

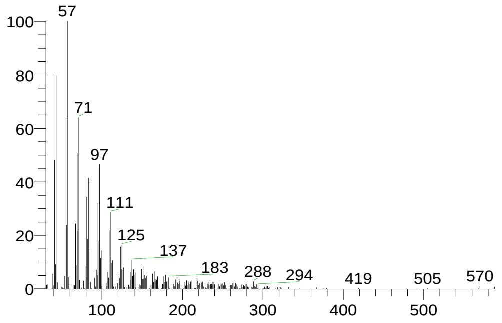

14-á-H-PREGNA
Formula C21H36, MW 288, CAS# NA, Entry# 178939
14-á-PREGNA

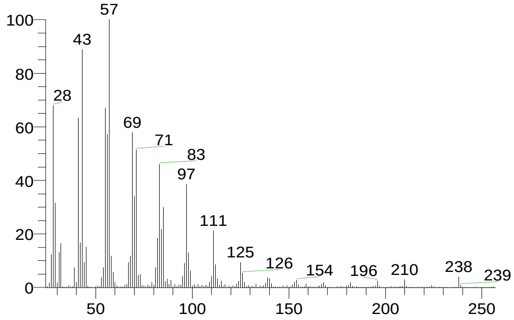

1-HEXADECANOL, 2-METHYL-
Formula C17H36O, MW 256, CAS# 2490-48-4, Entry# 146911
2-METHYLHEXADECAN-1-OL

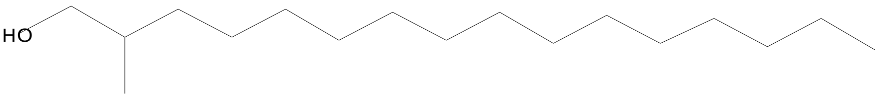

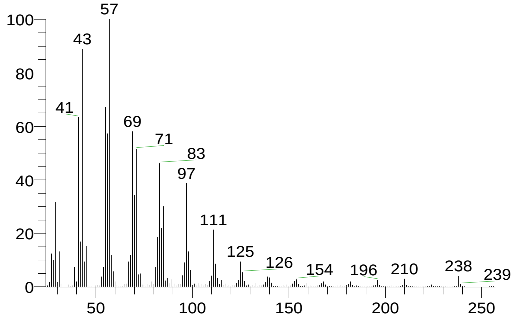

1-Hexadecanol, 2-methyl-
Formula C17H36O, MW 256, CAS# 2490-48-4, Entry# 20717
2-Methylhexadecan-1-ol

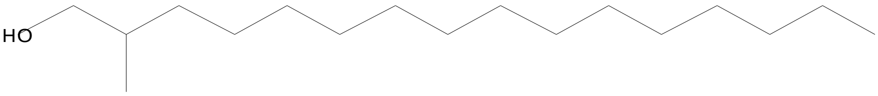

RT	Compound Name	Molecular Weight	Molecular Formula	Cas #	Area %	Library
24.06	DOTRIACONTANE	450	C32H66	544-85-4	1.14	WileyRegistry8e
24.06	14-á-H-PREGNA	288	C21H36	NA	1.14	WileyRegistry8e
24.06	1-Hexadecanol, 2-methyl-	256	C17H36O	2490-48-4	1.14	mainlib
24.06	1-HEXADECANOL, 2-METHYL-	256	C17H36O	2490-48-4	1.14	WileyRegistry8e

My GC-MS Report

Hit Spectrum | Compound Structure

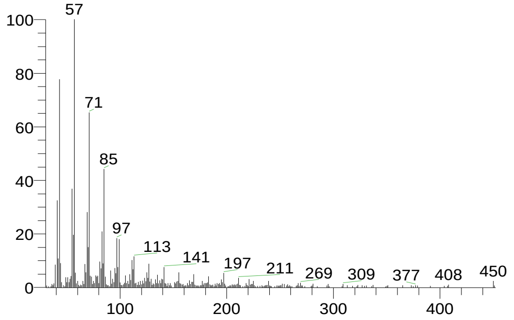

DOTRIACONTANE
Formula C32H66, MW 450, CAS# 544-85-4, Entry# 274478
AI3-52367

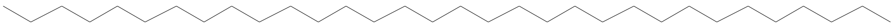

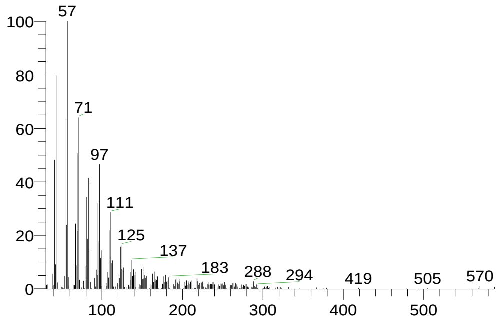

14-á-H-PREGNA
Formula C21H36, MW 288, CAS# NA, Entry# 178939
14-á-PREGNA

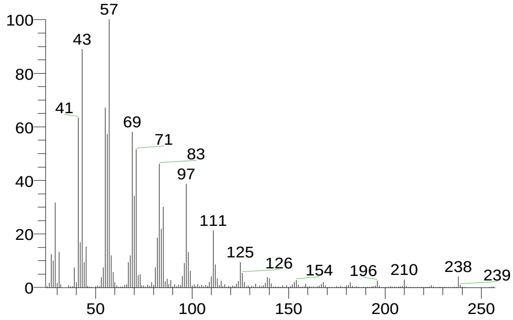

1-Hexadecanol, 2-methyl-
Formula C17H36O, MW 256, CAS# 2490-48-4, Entry# 20717
2-Methylhexadecan-1-ol

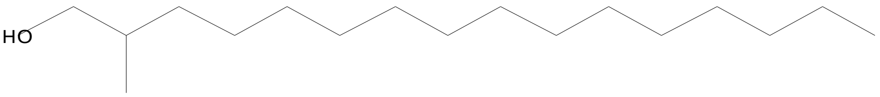

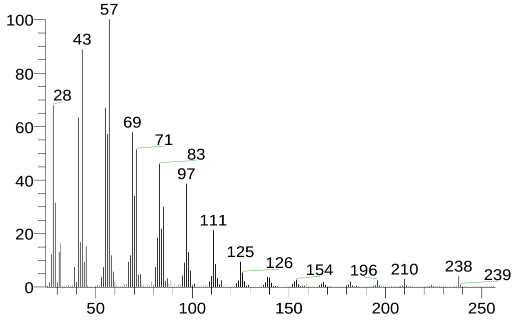

1-HEXADECANOL, 2-METHYL-
Formula C17H36O, MW 256, CAS# 2490-48-4, Entry# 146911
2-METHYLHEXADECAN-1-OL

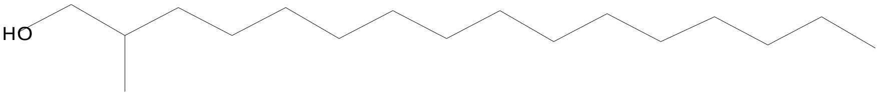

RT	Compound Name	Molecular Weight	Molecular Formula	Cas #	Area %	Library
24.23	DOTRIACONTANE	450	C32H66	544-85-4	0.33	WileyRegistry8e
24.23	ISOCHIAPIN B	346	C19H22O6	NA	0.33	WileyRegistry8e
24.23	ISOCHIAPIN B %2<	350	C19H26O6	NA	0.33	WileyRegistry8e
24.23	2,2-DIDEUTERO OCTADECANAL	270	C18H34D2O	56555-07-8	0.33	WileyRegistry8e

My GC-MS Report

Hit Spectrum | Compound Structure

DOTRIACONTANE
Formula C32H66, MW 450, CAS# 544-85-4, Entry# 274478
AI3-52367

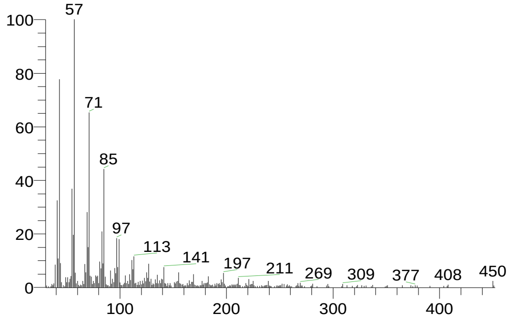

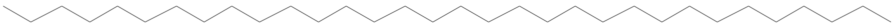

SI 735, RSI 743, WileyRegistry8e, Entry# 225807, CAS# NA, ISOCHIAPIN B

ISOCHIAPIN B
Formula C19H22O6, MW 346, CAS# NA, Entry# 225807

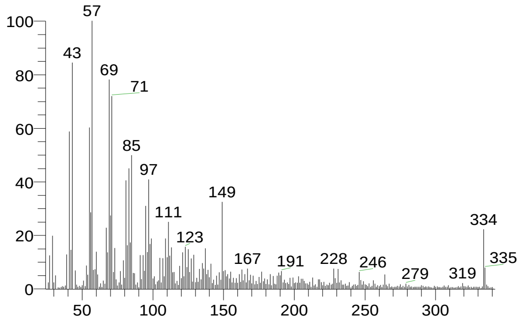

ISOCHIAPIN B %2<
Formula C19H26O6, MW 350, CAS# NA, Entry# 228500

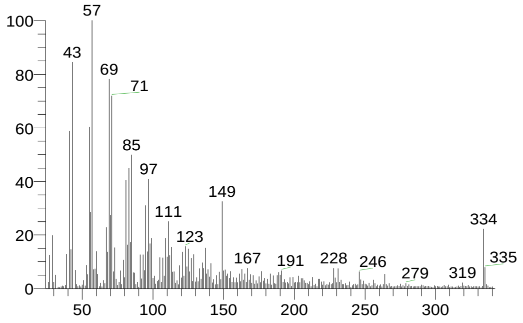

2,2-DIDEUTERO OCTADECANAL
Formula C18H34D2O, MW 270, CAS# 56555-07-8, Entry# 159359

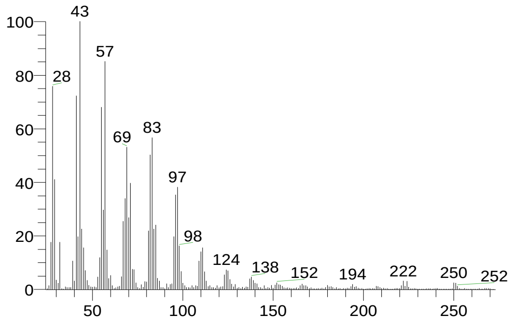

RT	Compound Name	Molecular Weight	Molecular Formula	Cas #	Area %	Library
24.37	ISOPROPYL TETRADECANOATE	270	C17H34O2	110-27-0	7.72	WileyRegistry8e
24.37	Isopropyl Myristate	270	C17H34O2	110-27-0	7.72	replib
24.37	ISOPROPYL MYRISTATE	270	C17H34O2	NA	7.72	WileyRegistry8e
24.37	Isopropyl Myristate	270	C17H34O2	110-27-0	7.72	replib

My GC-MS Report

Hit Spectrum | Compound Structure

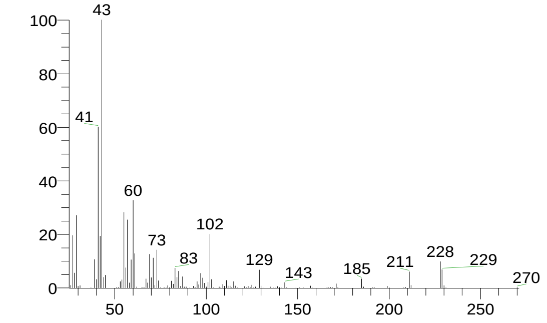

ISOPROPYL TETRADECANOATE
Formula C17H34O2, MW 270, CAS# 110-27-0, Entry# 161319
1-METHYLETHYL TETRADECANOATE

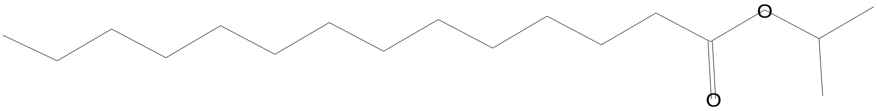

SI 784, RSI 845, replib, Entry# 2232, CAS# 110-27-0, Isopropyl Myristate

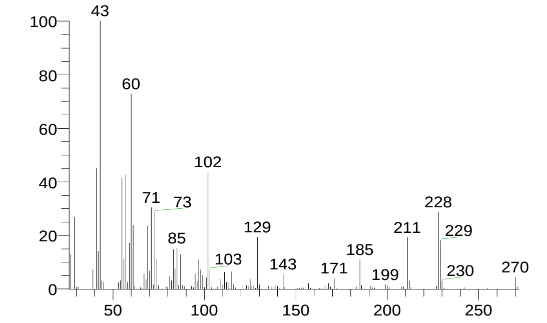

Isopropyl Myristate
Formula C17H34O2, MW 270, CAS# 110-27-0, Entry# 2232
Tetradecanoic acid, 1-methylethyl ester

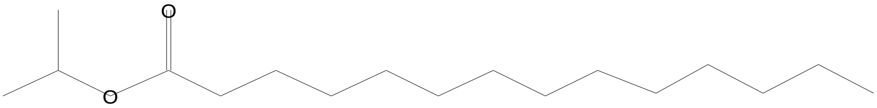

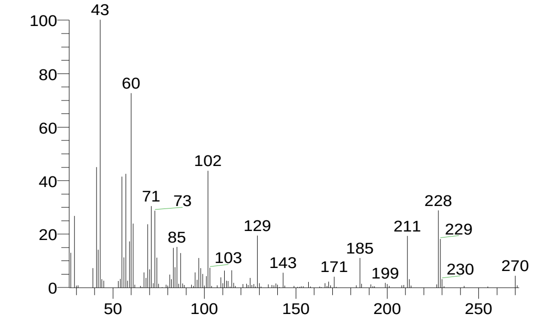

ISOPROPYL MYRISTATE
Formula C17H34O2, MW 270, CAS# NA, Entry# 384718
TETRADECANOIC ACID, 1-METHYLETHYL ESTER

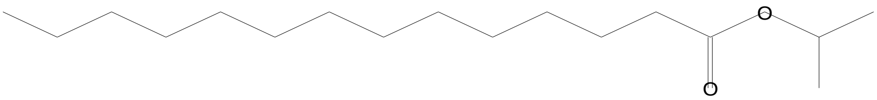

SI 780, RSI 830, replib, Entry# 2233, CAS# 110-27-0, Isopropyl Myristate

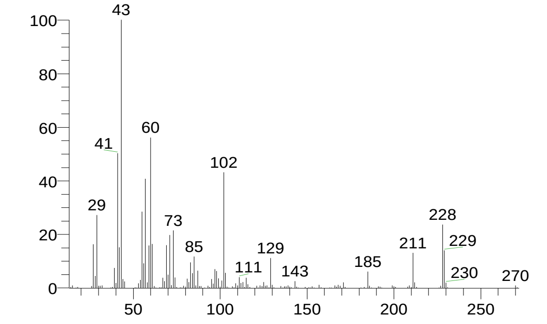

Isopropyl Myristate
Formula C17H34O2, MW 270, CAS# 110-27-0, Entry# 2233
Tetradecanoic acid, 1-methylethyl ester

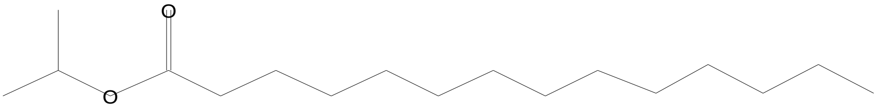

RT	Compound Name	Molecular Weight	Molecular Formula	Cas #	Area %	Library
24.86	DOTRIACONTANE	450	C32H66	544-85-4	0.10	WileyRegistry8e
24.86	14-á-H-PREGNA	288	C21H36	NA	0.10	WileyRegistry8e
24.86	ISOCHIAPIN B	346	C19H22O6	NA	0.10	WileyRegistry8e
24.86	ISOCHIAPIN B %2<	350	C19H26O6	NA	0.10	WileyRegistry8e

My GC-MS Report

Hit Spectrum | Compound Structure

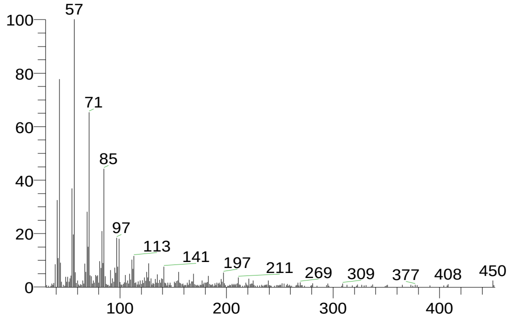

DOTRIACONTANE
Formula C32H66, MW 450, CAS# 544-85-4, Entry# 274478
AI3-52367

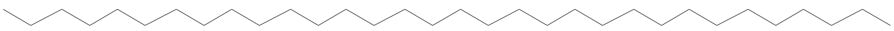

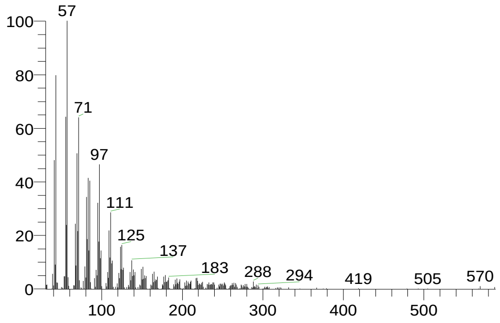

14-á-H-PREGNA
Formula C21H36, MW 288, CAS# NA, Entry# 178939
14-á-PREGNA

SI 760, RSI 772, WileyRegistry8e, Entry# 225807, CAS# NA, ISOCHIAPIN B

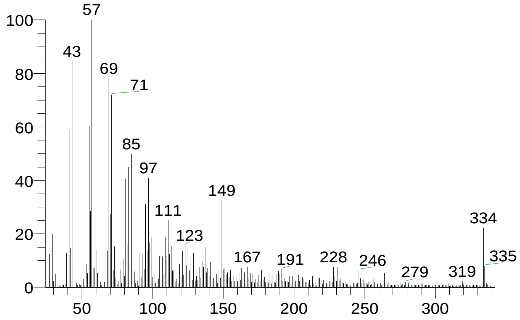

ISOCHIAPIN B
Formula C19H22O6, MW 346, CAS# NA, Entry# 225807

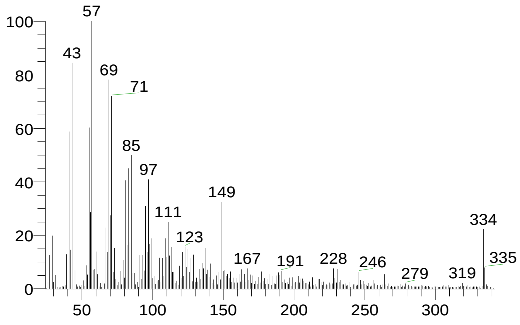

ISOCHIAPIN B %2<
Formula C19H26O6, MW 350, CAS# NA, Entry# 228500

RT	Compound Name	Molecular Weight	Molecular Formula	Cas #	Area %	Library
24.96	DOTRIACONTANE	450	C32H66	544-85-4	0.30	WileyRegistry8e
24.96	14-á-H-PREGNA	288	C21H36	NA	0.30	WileyRegistry8e
24.96	ISOCHIAPIN B	346	C19H22O6	NA	0.30	WileyRegistry8e
24.96	ISOCHIAPIN B %2<	350	C19H26O6	NA	0.30	WileyRegistry8e

My GC-MS Report

Hit Spectrum | Compound Structure

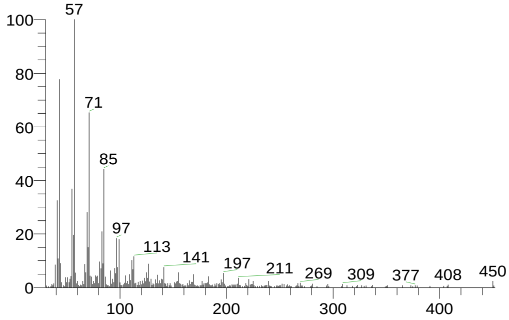

DOTRIACONTANE
Formula C32H66, MW 450, CAS# 544-85-4, Entry# 274478
AI3-52367

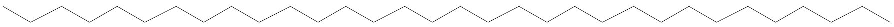

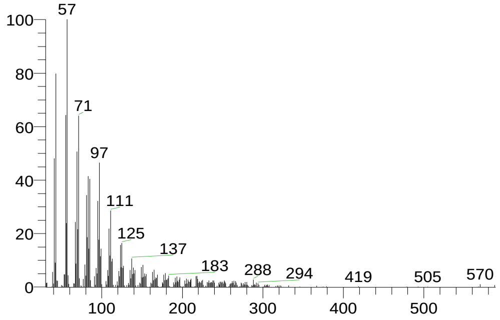

14-á-H-PREGNA
Formula C21H36, MW 288, CAS# NA, Entry# 178939
14-á-PREGNA

SI 750, RSI 763, WileyRegistry8e, Entry# 225807, CAS# NA, ISOCHIAPIN B

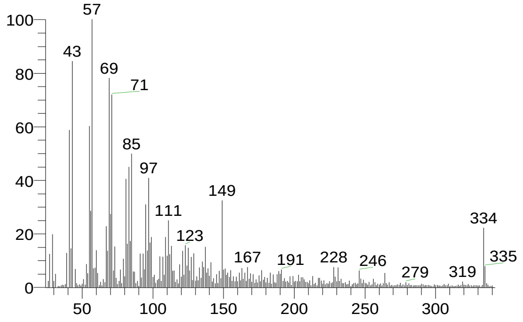

ISOCHIAPIN B
Formula C19H22O6, MW 346, CAS# NA, Entry# 225807

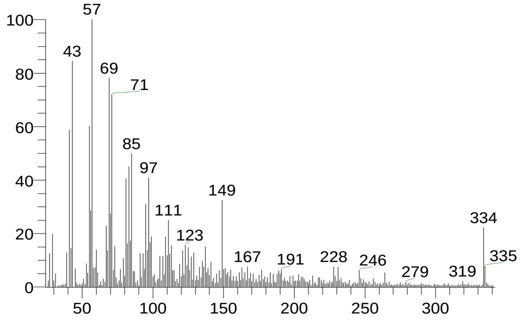

ISOCHIAPIN B %2<
Formula C19H26O6, MW 350, CAS# NA, Entry# 228500

RT	Compound Name	Molecular Weight	Molecular Formula	Cas #	Area %	Library
25.21	DOTRIACONTANE	450	C32H66	544-85-4	1.26	WileyRegistry8e
25.21	14-á-H-PREGNA	288	C21H36	NA	1.26	WileyRegistry8e
25.21	ISOCHIAPIN B	346	C19H22O6	NA	1.26	WileyRegistry8e
25.21	ISOCHIAPIN B %2<	350	C19H26O6	NA	1.26	WileyRegistry8e

My GC-MS Report

Hit Spectrum | Compound Structure

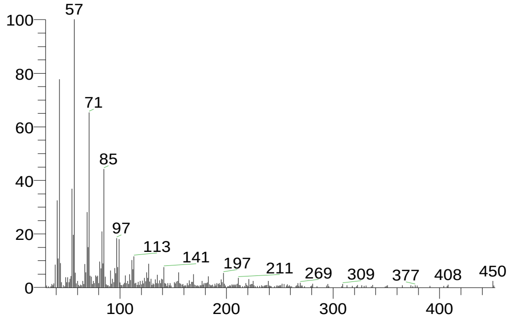

DOTRIACONTANE
Formula C32H66, MW 450, CAS# 544-85-4, Entry# 274478
AI3-52367

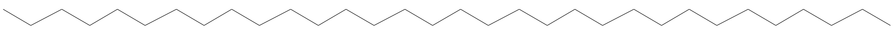

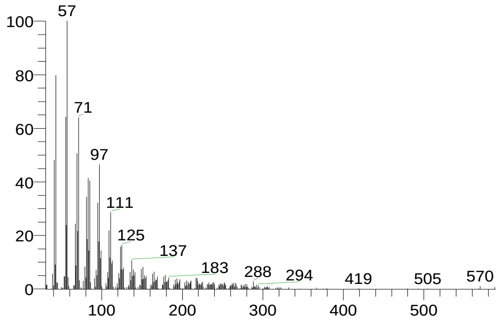

14-á-H-PREGNA
Formula C21H36, MW 288, CAS# NA, Entry# 178939
14-á-PREGNA

SI 722, RSI 743, WileyRegistry8e, Entry# 225807, CAS# NA, ISOCHIAPIN B

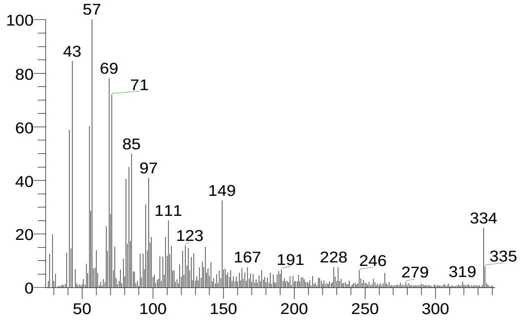

ISOCHIAPIN B
Formula C19H22O6, MW 346, CAS# NA, Entry# 225807

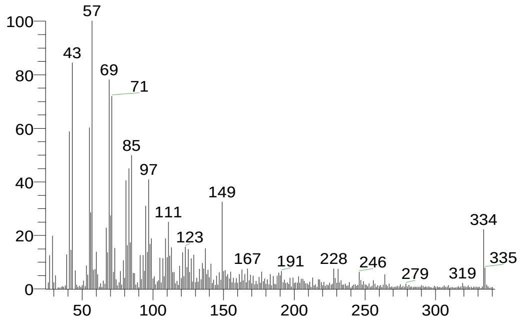

ISOCHIAPIN B %2<
Formula C19H26O6, MW 350, CAS# NA, Entry# 228500

RT	Compound Name	Molecular Weight	Molecular Formula	Cas #	Area %	Library
25.66	DOTRIACONTANE	450	C32H66	544-85-4	5.60	WileyRegistry8e
25.66	1-Hexadecanol, 2-methyl-	256	C17H36O	2490-48-4	5.60	mainlib
25.66	1-HEXADECANOL, 2-METHYL-	256	C17H36O	2490-48-4	5.60	WileyRegistry8e
25.66	14-á-H-PREGNA	288	C21H36	NA	5.60	WileyRegistry8e

My GC-MS Report

Hit Spectrum | Compound Structure

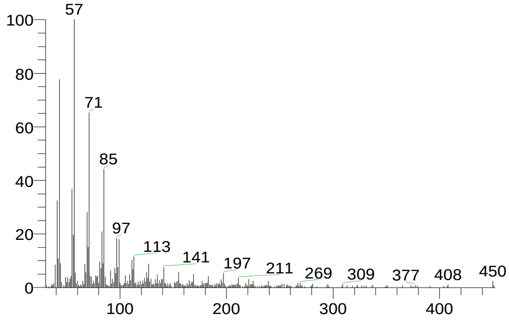

DOTRIACONTANE
Formula C32H66, MW 450, CAS# 544-85-4, Entry# 274478
AI3-52367

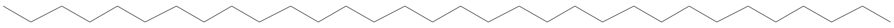

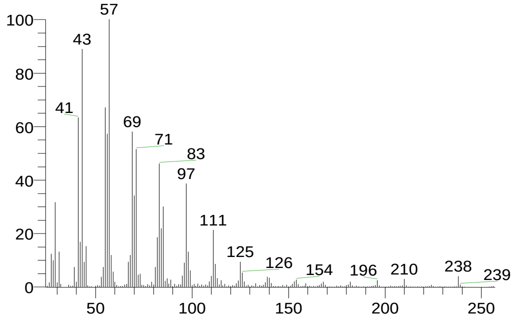

1-Hexadecanol, 2-methyl-
Formula C17H36O, MW 256, CAS# 2490-48-4, Entry# 20717
2-Methylhexadecan-1-ol

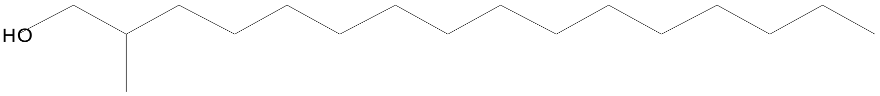

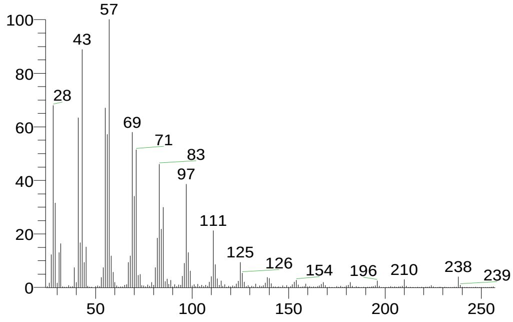

1-HEXADECANOL, 2-METHYL-
Formula C17H36O, MW 256, CAS# 2490-48-4, Entry# 146911
2-METHYLHEXADECAN-1-OL

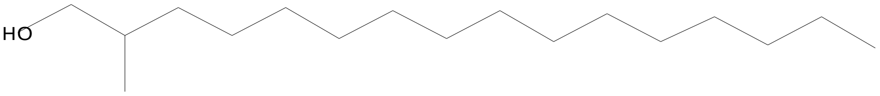

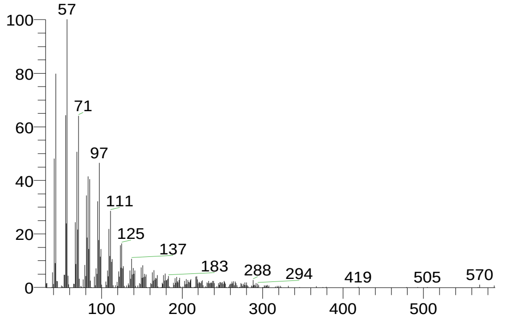

14-á-H-PREGNA
Formula C21H36, MW 288, CAS# NA, Entry# 178939
14-á-PREGNA

RT	Compound Name	Molecular Weight	Molecular Formula	Cas #	Area %	Library
25.74	DOTRIACONTANE	450	C32H66	544-85-4	4.19	WileyRegistry8e
25.74	14-á-H-PREGNA	288	C21H36	NA	4.19	WileyRegistry8e
25.74	1-HEXADECANOL, 2-METHYL-	256	C17H36O	2490-48-4	4.19	WileyRegistry8e
25.74	1-Hexadecanol, 2-methyl-	256	C17H36O	2490-48-4	4.19	mainlib

My GC-MS Report

Hit Spectrum | Compound Structure

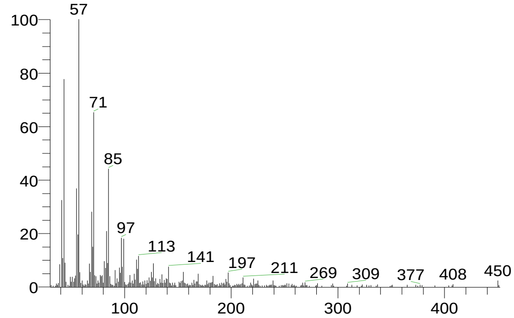

DOTRIACONTANE
Formula C32H66, MW 450, CAS# 544-85-4, Entry# 274478
AI3-52367

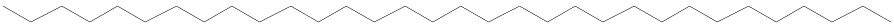

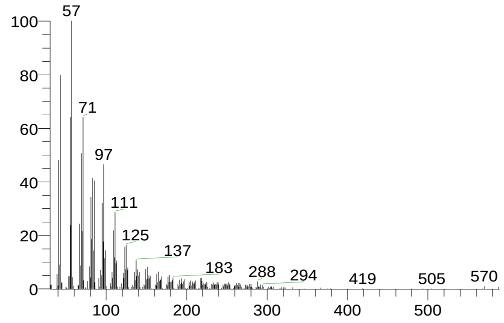

14-á-H-PREGNA
Formula C21H36, MW 288, CAS# NA, Entry# 178939
14-á-PREGNA

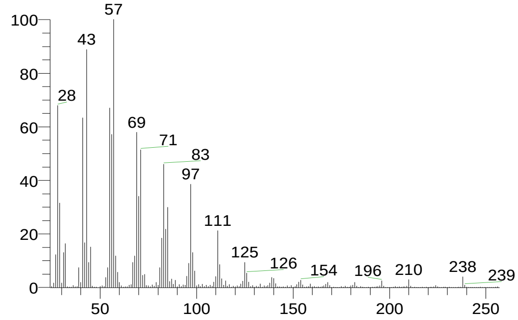

1-HEXADECANOL, 2-METHYL-
Formula C17H36O, MW 256, CAS# 2490-48-4, Entry# 146911
2-METHYLHEXADECAN-1-OL

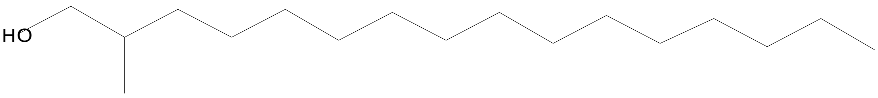

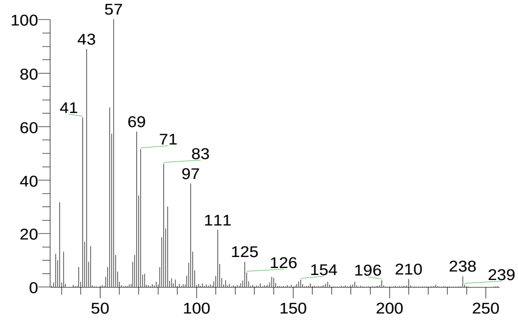

1-Hexadecanol, 2-methyl-
Formula C17H36O, MW 256, CAS# 2490-48-4, Entry# 20717
2-Methylhexadecan-1-ol

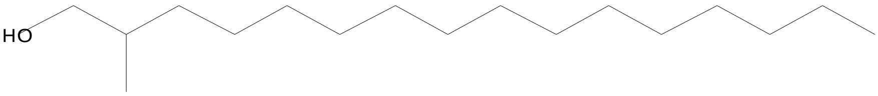

RT	Compound Name	Molecular Weight	Molecular Formula	Cas #	Area %	Library
26.08	DOTRIACONTANE	450	C32H66	544-85-4	0.46	WileyRegistry8e
26.08	14-á-H-PREGNA	288	C21H36	NA	0.46	WileyRegistry8e
26.08	14-á-H-PREGNA	288	C21H36	NA	0.46	WileyRegistry8e
26.08	Tetrapentacontane, 1,54-dibromo-	914	C54H108Br2	NA	0.46	mainlib

My GC-MS Report

Hit Spectrum | Compound Structure

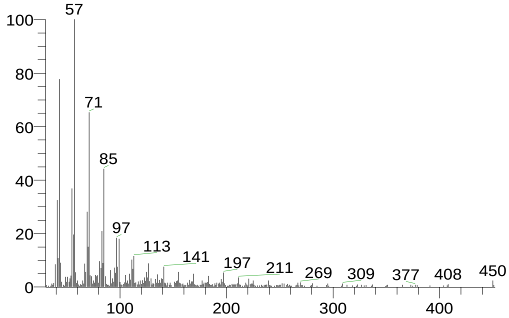

DOTRIACONTANE
Formula C32H66, MW 450, CAS# 544-85-4, Entry# 274478
AI3-52367

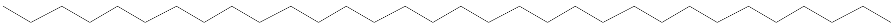

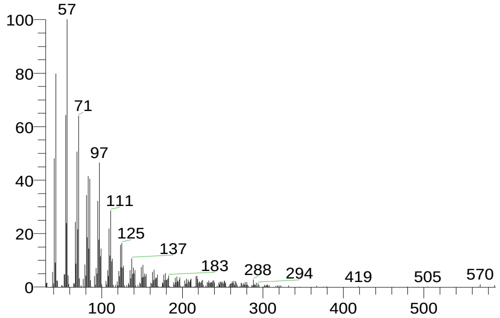

14-á-H-PREGNA
Formula C21H36, MW 288, CAS# NA, Entry# 178939
14-á-PREGNA

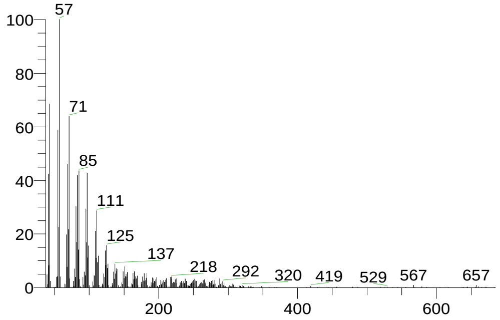

14-á-H-PREGNA
Formula C21H36, MW 288, CAS# NA, Entry# 178938
14-á-PREGNA

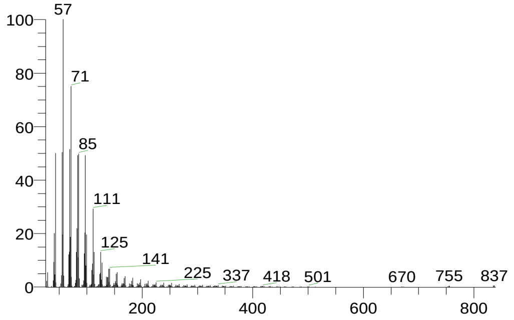

Tetrapentacontane, 1,54-dibromo-
Formula C54H108Br2, MW 914, CAS# NA, Entry# 21687

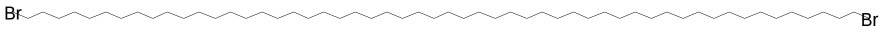

RT	Compound Name	Molecular Weight	Molecular Formula	Cas #	Area %	Library
26.25	DOTRIACONTANE	450	C32H66	544-85-4	4.18	WileyRegistry8e
26.25	14-á-H-PREGNA	288	C21H36	NA	4.18	WileyRegistry8e
26.25	14-á-H-PREGNA	288	C21H36	NA	4.18	WileyRegistry8e
26.25	ISOCHIAPIN B	346	C19H22O6	NA	4.18	WileyRegistry8e

My GC-MS Report

Hit Spectrum | Compound Structure

DOTRIACONTANE
Formula C32H66, MW 450, CAS# 544-85-4, Entry# 274478
AI3-52367

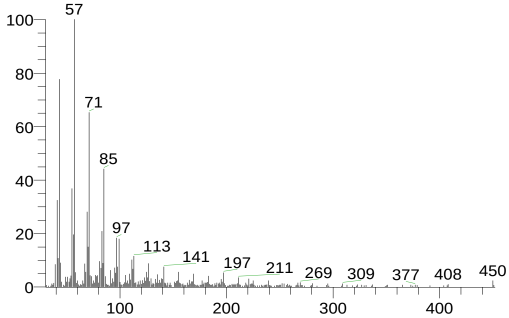

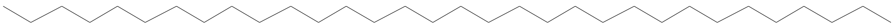

14-á-H-PREGNA
Formula C21H36, MW 288, CAS# NA, Entry# 178939
14-á-PREGNA

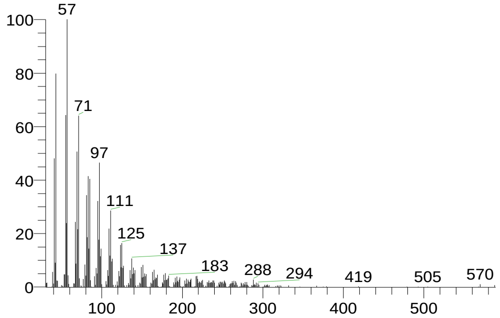

14-á-H-PREGNA
Formula C21H36, MW 288, CAS# NA, Entry# 178938
14-á-PREGNA

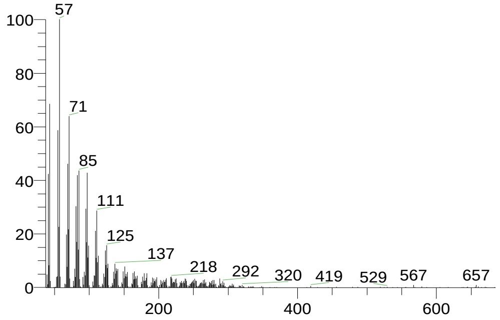

SI 739, RSI 751, WileyRegistry8e, Entry# 225807, CAS# NA, ISOCHIAPIN B

ISOCHIAPIN B
Formula C19H22O6, MW 346, CAS# NA, Entry# 225807

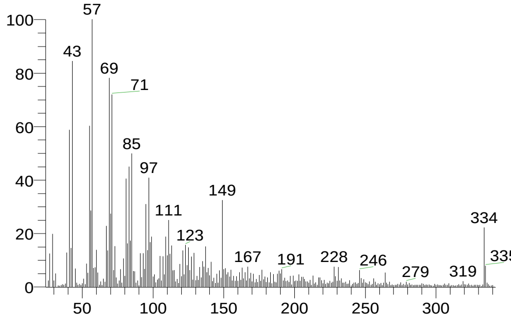

RT	Compound Name	Molecular Weight	Molecular Formula	Cas #	Area %	Library
26.79	DOTRIACONTANE	450	C32H66	544-85-4	3.79	WileyRegistry8e
26.79	14-á-H-PREGNA	288	C21H36	NA	3.79	WileyRegistry8e
26.79	14-á-H-PREGNA	288	C21H36	NA	3.79	WileyRegistry8e
26.79	2,2-DIDEUTERO OCTADECANAL	270	C18H34D2O	56555-07-8	3.79	WileyRegistry8e

My GC-MS Report

Hit Spectrum	Compound Structure

DOTRIACONTANE
Formula C32H66, MW 450, CAS# 544-85-4, Entry# 274478
AI3-52367

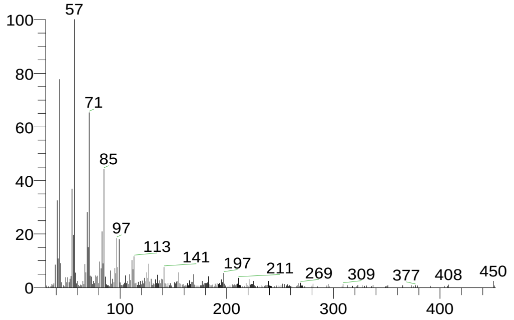

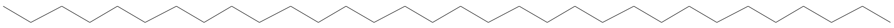

14-á-H-PREGNA
Formula C21H36, MW 288, CAS# NA, Entry# 178939
14-á-PREGNA

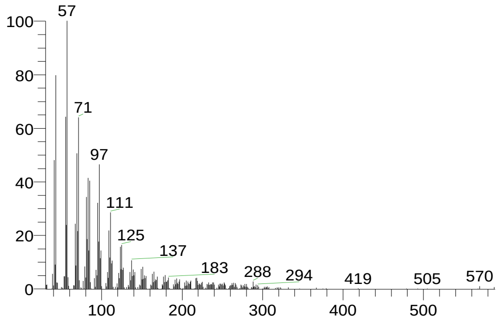

14-á-H-PREGNA
Formula C21H36, MW 288, CAS# NA, Entry# 178938
14-á-PREGNA

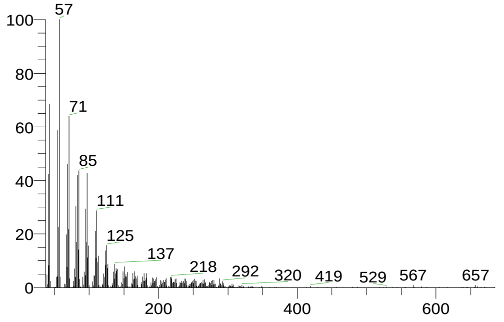

2,2-DIDEUTERO OCTADECANAL
Formula C18H34D2O, MW 270, CAS# 56555-07-8, Entry# 159359

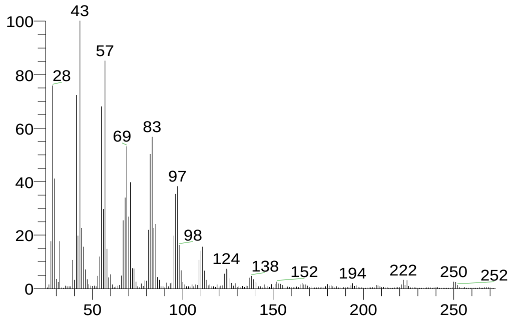

RT	Compound Name	Molecular Weight	Molecular Formula	Cas #	Area %	Library
27.30	14-á-H-PREGNA	288	C21H36	NA	2.97	WileyRegistry8e
27.30	14-á-H-PREGNA	288	C21H36	NA	2.97	WileyRegistry8e
27.30	HAHNFETT	0	N/A	NA	2.97	WileyRegistry8e
27.30	HAHNFETT	0	N/A	NA	2.97	WileyRegistry8e

My GC-MS Report

Hit Spectrum | Compound Structure

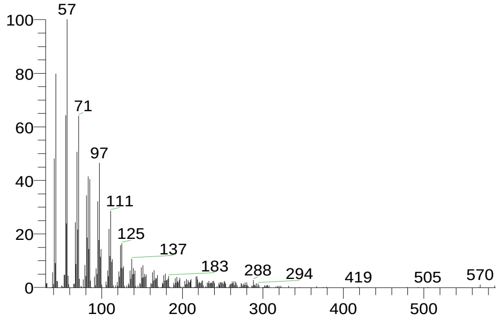

14-á-H-PREGNA
Formula C21H36, MW 288, CAS# NA, Entry# 178939
14-á-PREGNA

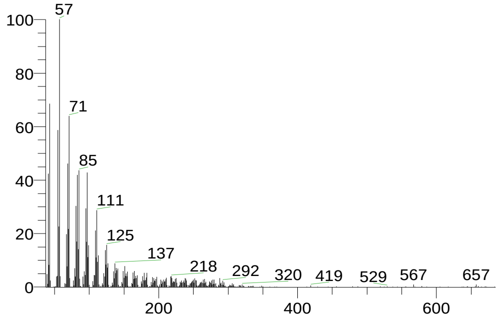

14-á-H-PREGNA
Formula C21H36, MW 288, CAS# NA, Entry# 178938
14-á-PREGNA

SI 706, RSI 712, WileyRegistry8e, Entry# 305496, CAS# NA, HAHNFETT

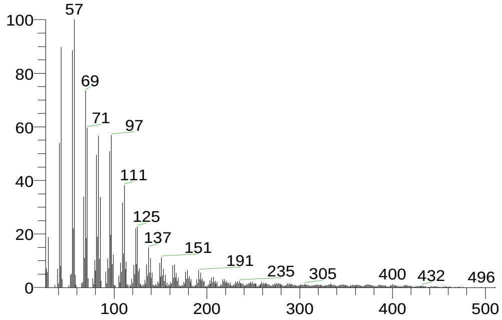

HAHNFETT
Formula , MW 0, CAS# NA, Entry# 305496

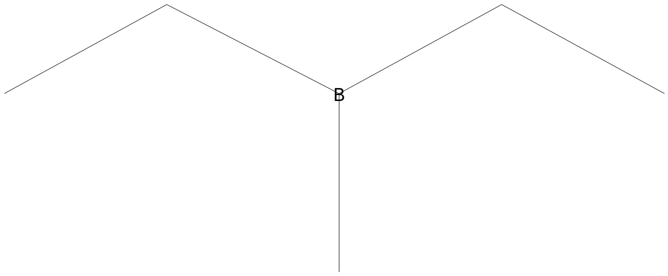

SI 706, RSI 712, WileyRegistry8e, Entry# 391160, CAS# NA, HAHNFETT

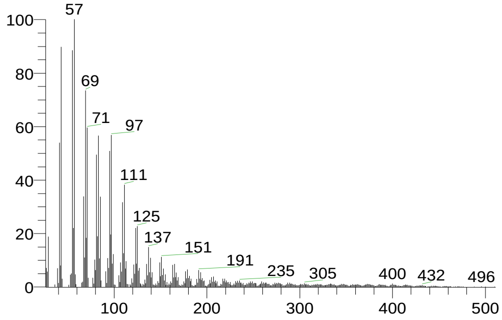

HAHNFETT
Formula , MW 0, CAS# NA, Entry# 391160

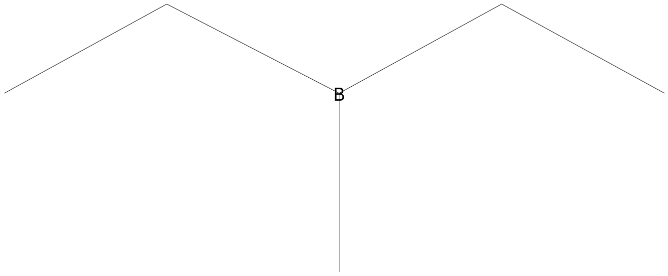

RT	Compound Name	Molecular Weight	Molecular Formula	Cas #	Area %	Library
27.58	DOTRIACONTANE	450	C32H66	544-85-4	2.35	WileyRegistry8e
27.58	14-á-H-PREGNA	288	C21H36	NA	2.35	WileyRegistry8e
27.58	ISOCHIAPIN B	346	C19H22O6	NA	2.35	WileyRegistry8e
27.58	ISOCHIAPIN B %2<	350	C19H26O6	NA	2.35	WileyRegistry8e

My GC-MS Report

Hit Spectrum | Compound Structure

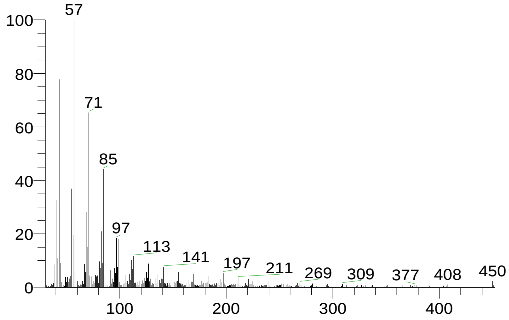

DOTRIACONTANE
Formula C32H66, MW 450, CAS# 544-85-4, Entry# 274478
AI3-52367

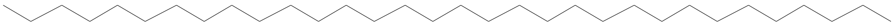

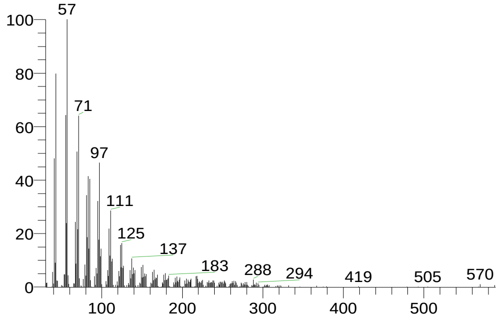

14-á-H-PREGNA
Formula C21H36, MW 288, CAS# NA, Entry# 178939
14-á-PREGNA

SI 748, RSI 760, WileyRegistry8e, Entry# 225807, CAS# NA, ISOCHIAPIN B

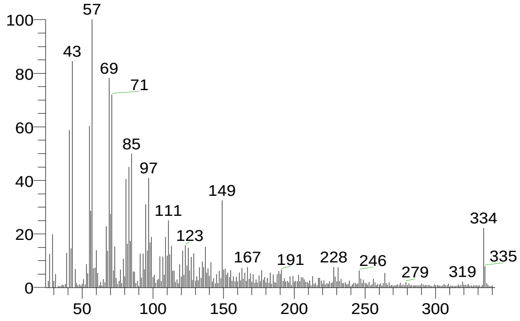

ISOCHIAPIN B
Formula C19H22O6, MW 346, CAS# NA, Entry# 225807

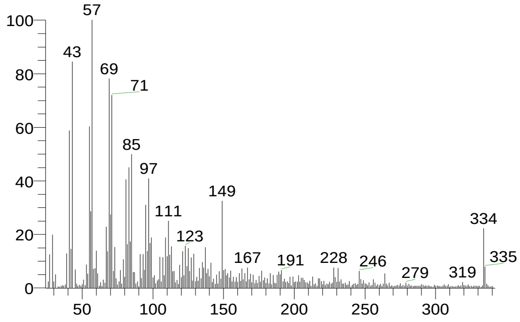

ISOCHIAPIN B %2<
Formula C19H26O6, MW 350, CAS# NA, Entry# 228500

RT	Compound Name	Molecular Weight	Molecular Formula	Cas #	Area %	Library
28.05	DOTRIACONTANE	450	C32H66	544-85-4	13.79	WileyRegistry8e
28.05	14-á-H-PREGNA	288	C21H36	NA	13.79	WileyRegistry8e
28.05	14-á-H-PREGNA	288	C21H36	NA	13.79	WileyRegistry8e
28.05	1-HEXADECANOL, 2-METHYL-	256	C17H36O	2490-48-4	13.79	WileyRegistry8e

My GC-MS Report

Hit Spectrum | Compound Structure

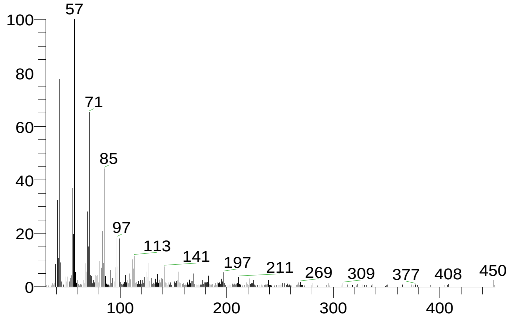

DOTRIACONTANE
Formula C32H66, MW 450, CAS# 544-85-4, Entry# 274478
AI3-52367

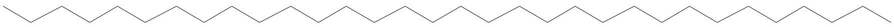

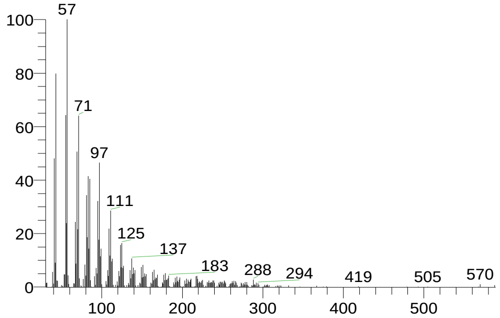

14-á-H-PREGNA
Formula C21H36, MW 288, CAS# NA, Entry# 178939
14-á-PREGNA

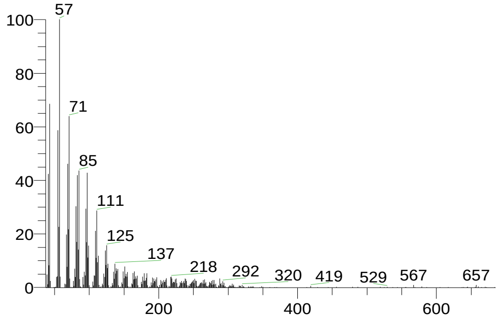

14-á-H-PREGNA
Formula C21H36, MW 288, CAS# NA, Entry# 178938
14-á-PREGNA

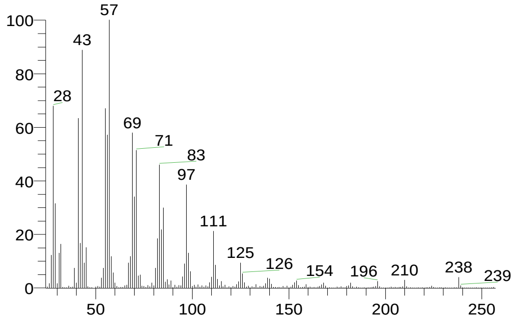

1-HEXADECANOL, 2-METHYL-
Formula C17H36O, MW 256, CAS# 2490-48-4, Entry# 146911
2-METHYLHEXADECAN-1-OL

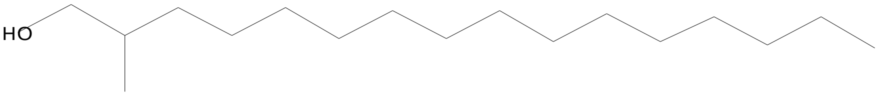

RT	Compound Name	Molecular Weight	Molecular Formula	Cas #	Area %	Library
28.25	DOTRIACONTANE	450	C32H66	544-85-4	0.28	WileyRegistry8e
28.25	14-á-H-PREGNA	288	C21H36	NA	0.28	WileyRegistry8e
28.25	14-á-H-PREGNA	288	C21H36	NA	0.28	WileyRegistry8e
28.25	1-HEXADECANOL, 2-METHYL-	256	C17H36O	2490-48-4	0.28	WileyRegistry8e

My GC-MS Report

Hit Spectrum	Compound Structure

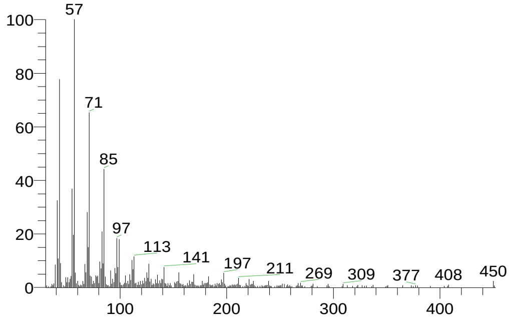

DOTRIACONTANE
Formula C32H66, MW 450, CAS# 544-85-4, Entry# 274478
AI3-52367

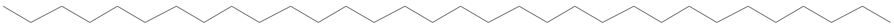

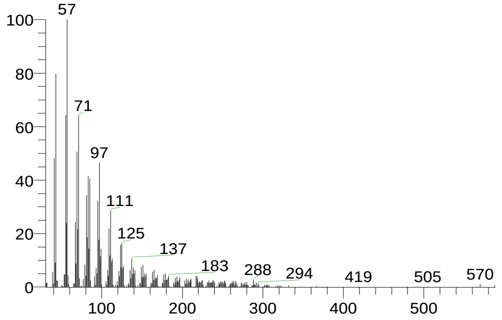

14-á-H-PREGNA
Formula C21H36, MW 288, CAS# NA, Entry# 178939
14-á-PREGNA

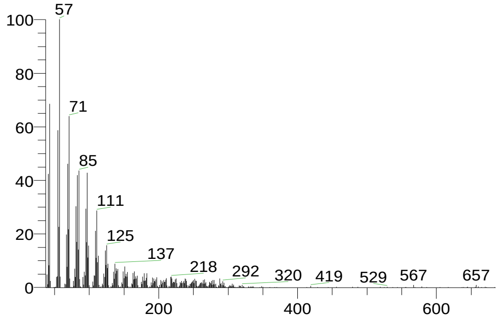

14-á-H-PREGNA
Formula C21H36, MW 288, CAS# NA, Entry# 178938
14-á-PREGNA

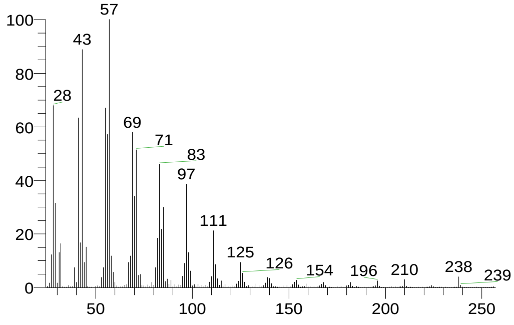

1-HEXADECANOL, 2-METHYL-
Formula C17H36O, MW 256, CAS# 2490-48-4, Entry# 146911
2-METHYLHEXADECAN-1-OL

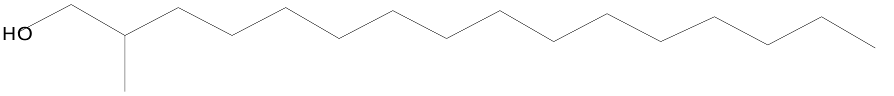

RT	Compound Name	Molecular Weight	Molecular Formula	Cas #	Area %	Library
28.49	DOTRIACONTANE	450	C32H66	544-85-4	3.50	WileyRegistry8e
28.49	14-á-H-PREGNA	288	C21H36	NA	3.50	WileyRegistry8e
28.49	14-á-H-PREGNA	288	C21H36	NA	3.50	WileyRegistry8e
28.49	2,2-DIDEUTERO OCTADECANAL	270	C18H34D2O	56555-07-8	3.50	WileyRegistry8e

My GC-MS Report

Hit Spectrum | Compound Structure

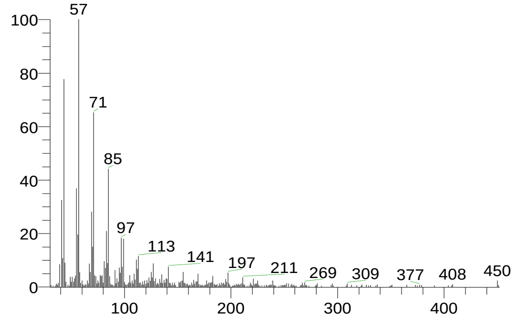

DOTRIACONTANE
Formula C32H66, MW 450, CAS# 544-85-4, Entry# 274478
AI3-52367

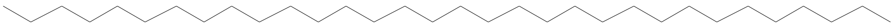

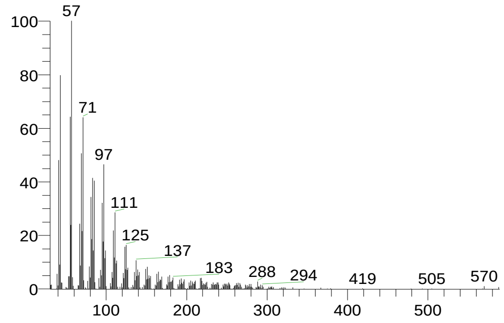

14-á-H-PREGNA
Formula C21H36, MW 288, CAS# NA, Entry# 178939
14-á-PREGNA

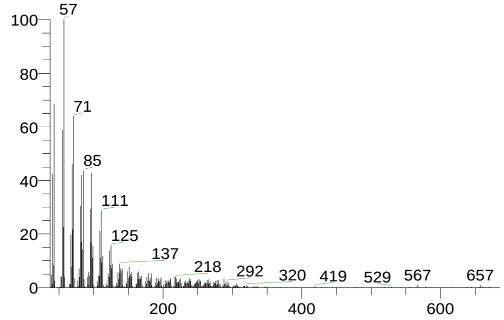

14-á-H-PREGNA
Formula C21H36, MW 288, CAS# NA, Entry# 178938
14-á-PREGNA

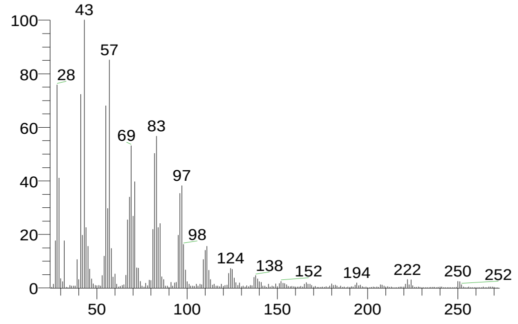

2,2-DIDEUTERO OCTADECANAL
Formula C18H34D2O, MW 270, CAS# 56555-07-8, Entry# 159359

RT	Compound Name	Molecular Weight	Molecular Formula	Cas #	Area %	Library
28.88	DOTRIACONTANE	450	C32H66	544-85-4	4.29	WileyRegistry8e
28.88	14-á-H-PREGNA	288	C21H36	NA	4.29	WileyRegistry8e
28.88	14-á-H-PREGNA	288	C21H36	NA	4.29	WileyRegistry8e
28.88	ISOCHIAPIN B	346	C19H22O6	NA	4.29	WileyRegistry8e

My GC-MS Report

Hit Spectrum | Compound Structure

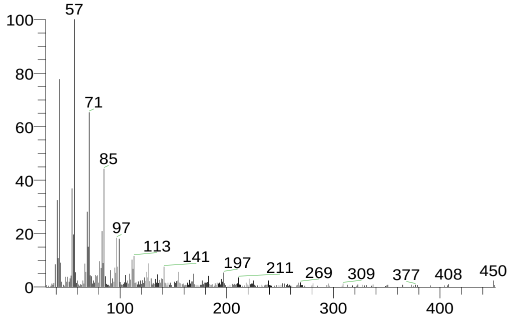

DOTRIACONTANE
Formula C32H66, MW 450, CAS# 544-85-4, Entry# 274478
AI3-52367

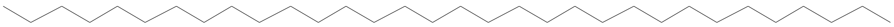

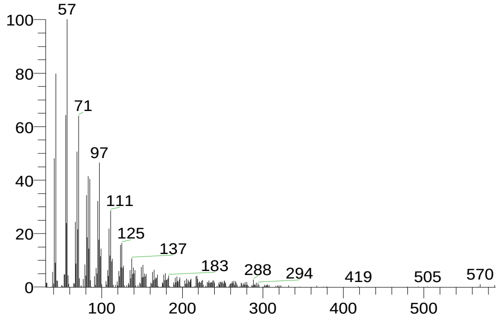

14-á-H-PREGNA
Formula C21H36, MW 288, CAS# NA, Entry# 178939
14-á-PREGNA

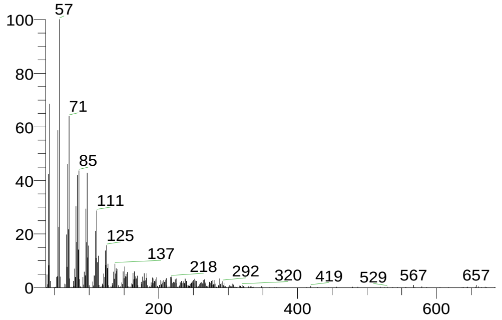

14-á-H-PREGNA
Formula C21H36, MW 288, CAS# NA, Entry# 178938
14-á-PREGNA

SI 730, RSI 749, WileyRegistry8e, Entry# 225807, CAS# NA, ISOCHIAPIN B

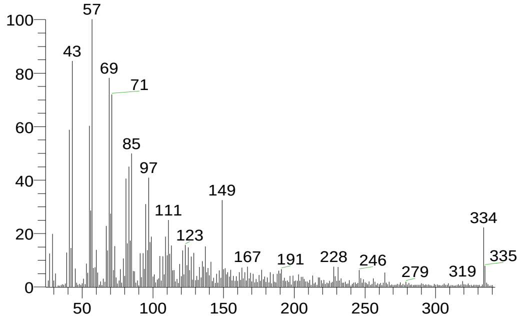

ISOCHIAPIN B
Formula C19H22O6, MW 346, CAS# NA, Entry# 225807

RT	Compound Name	Molecular Weight	Molecular Formula	Cas #	Area %	Library
29.10	DOTRIACONTANE	450	C32H66	544-85-4	4.04	WileyRegistry8e
29.10	14-á-H-PREGNA	288	C21H36	NA	4.04	WileyRegistry8e
29.10	14-á-H-PREGNA	288	C21H36	NA	4.04	WileyRegistry8e
29.10	ISOCHIAPIN B	346	C19H22O6	NA	4.04	WileyRegistry8e

My GC-MS Report

Hit Spectrum | Compound Structure

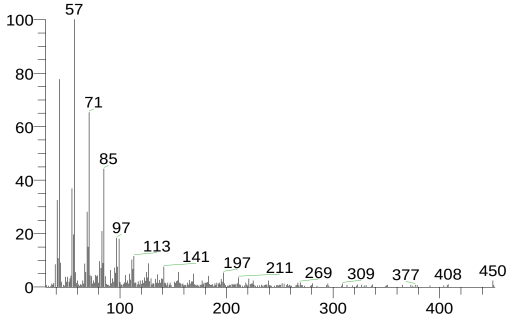

DOTRIACONTANE
Formula C32H66, MW 450, CAS# 544-85-4, Entry# 274478
AI3-52367

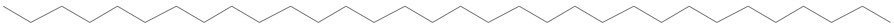

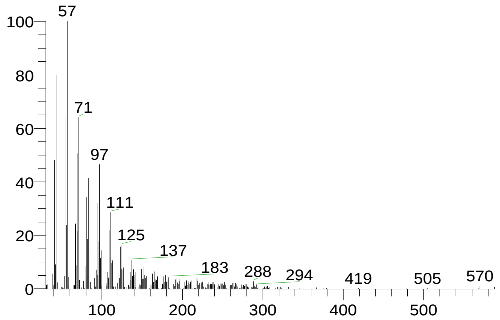

14-á-H-PREGNA
Formula C21H36, MW 288, CAS# NA, Entry# 178939
14-á-PREGNA

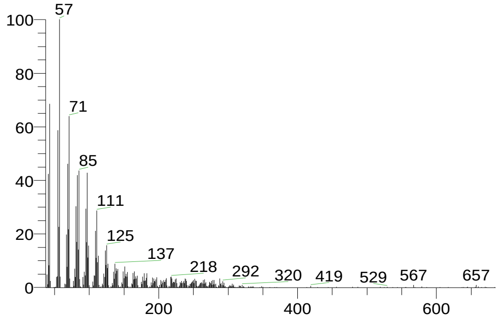

14-á-H-PREGNA
Formula C21H36, MW 288, CAS# NA, Entry# 178938
14-á-PREGNA

SI 731, RSI 748, WileyRegistry8e, Entry# 225807, CAS# NA, ISOCHIAPIN B

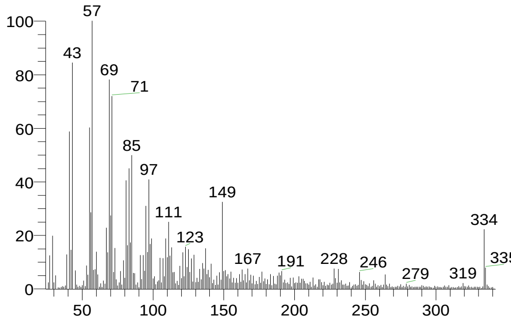

ISOCHIAPIN B
Formula C19H22O6, MW 346, CAS# NA, Entry# 225807

RT	Compound Name	Molecular Weight	Molecular Formula	Cas #	Area %	Library
29.33	DOTRIACONTANE	450	C32H66	544-85-4	1.96	WileyRegistry8e
29.33	14-á-H-PREGNA	288	C21H36	NA	1.96	WileyRegistry8e
29.33	14-á-H-PREGNA	288	C21H36	NA	1.96	WileyRegistry8e
29.33	17-Pentatriacontene	490	C35H70	6971-40-0	1.96	mainlib

My GC-MS Report

Hit Spectrum | Compound Structure

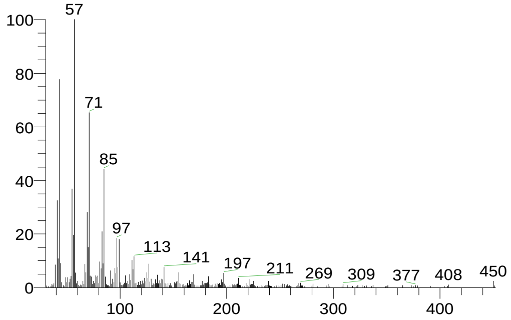

DOTRIACONTANE
Formula C32H66, MW 450, CAS# 544-85-4, Entry# 274478
AI3-52367

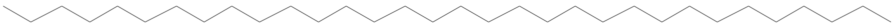

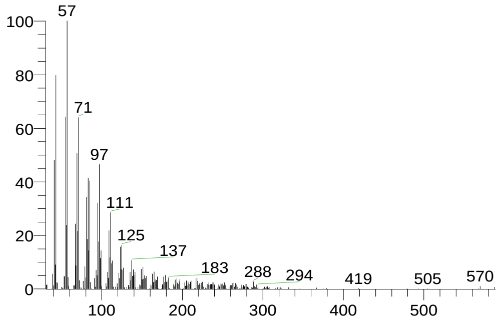

14-á-H-PREGNA
Formula C21H36, MW 288, CAS# NA, Entry# 178939
14-á-PREGNA

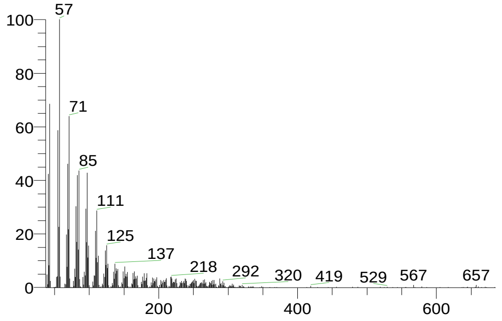

14-á-H-PREGNA
Formula C21H36, MW 288, CAS# NA, Entry# 178938
14-á-PREGNA

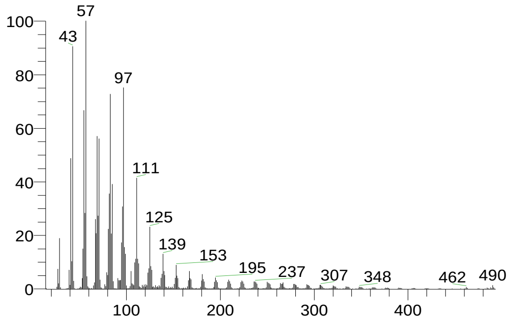

17-Pentatriacontene
Formula C35H70, MW 490, CAS# 6971-40-0, Entry# 21018
(17E)-17-Pentatriacontene #

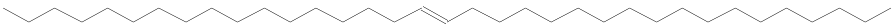

RT	Compound Name	Molecular Weight	Molecular Formula	Cas #	Area %	Library
29.55	DOTRIACONTANE	450	C32H66	544-85-4	2.26	WileyRegistry8e
29.55	14-á-H-PREGNA	288	C21H36	NA	2.26	WileyRegistry8e
29.55	14-á-H-PREGNA	288	C21H36	NA	2.26	WileyRegistry8e
29.55	ISOCHIAPIN B	346	C19H22O6	NA	2.26	WileyRegistry8e

My GC-MS Report

Hit Spectrum | Compound Structure

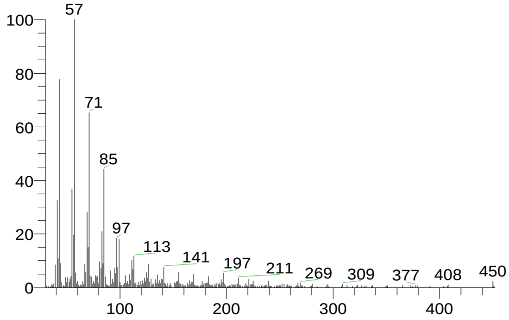

DOTRIACONTANE
Formula C32H66, MW 450, CAS# 544-85-4, Entry# 274478
AI3-52367

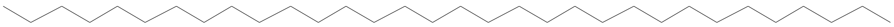

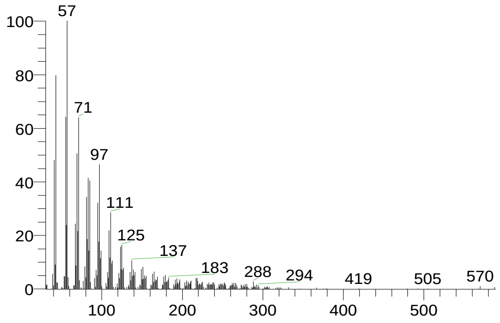

14-á-H-PREGNA
Formula C21H36, MW 288, CAS# NA, Entry# 178939
14-á-PREGNA

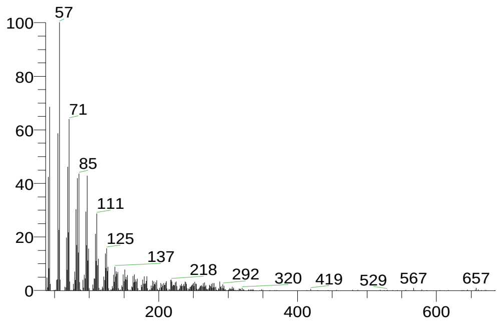

14-á-H-PREGNA
Formula C21H36, MW 288, CAS# NA, Entry# 178938
14-á-PREGNA

SI 740, RSI 755, WileyRegistry8e, Entry# 225807, CAS# NA, ISOCHIAPIN B

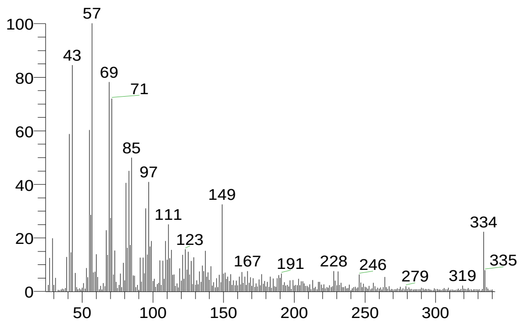

ISOCHIAPIN B
Formula C19H22O6, MW 346, CAS# NA, Entry# 225807

RT	Compound Name	Molecular Weight	Molecular Formula	Cas #	Area %	Library
29.73	DOTRIACONTANE	450	C32H66	544-85-4	0.67	WileyRegistry8e
29.73	14-á-H-PREGNA	288	C21H36	NA	0.67	WileyRegistry8e
29.73	14-á-H-PREGNA	288	C21H36	NA	0.67	WileyRegistry8e
29.73	17-Pentatriacontene	490	C35H70	6971-40-0	0.67	mainlib

My GC-MS Report

Hit Spectrum | Compound Structure

DOTRIACONTANE
Formula C32H66, MW 450, CAS# 544-85-4, Entry# 274478
AI3-52367

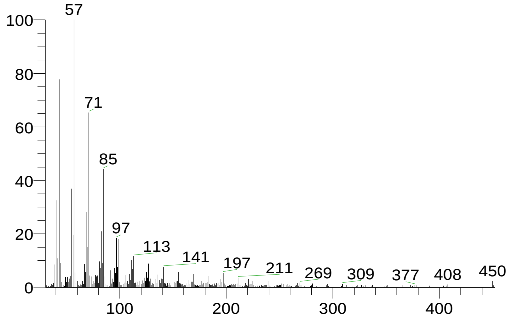

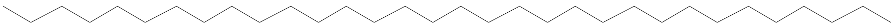

14-á-H-PREGNA
Formula C21H36, MW 288, CAS# NA, Entry# 178939
14-á-PREGNA

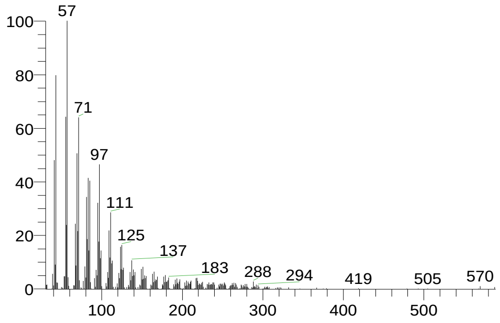

14-á-H-PREGNA
Formula C21H36, MW 288, CAS# NA, Entry# 178938
14-á-PREGNA

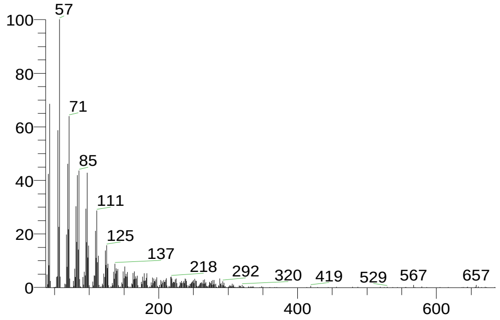

17-Pentatriacontene
Formula C35H70, MW 490, CAS# 6971-40-0, Entry# 21018
(17E)-17-Pentatriacontene #

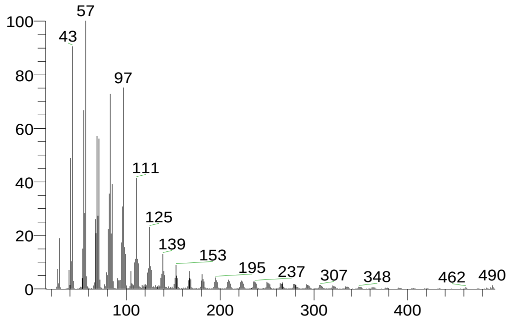

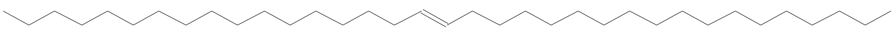

RT	Compound Name	Molecular Weight	Molecular Formula	Cas #	Area %	Library
29.80	DOTRIACONTANE	450	C32H66	544-85-4	0.10	WileyRegistry8e
29.80	14-á-H-PREGNA	288	C21H36	NA	0.10	WileyRegistry8e
29.80	14-á-H-PREGNA	288	C21H36	NA	0.10	WileyRegistry8e
29.80	ISOCHIAPIN B	346	C19H22O6	NA	0.10	WileyRegistry8e

My GC-MS Report

Hit Spectrum | Compound Structure

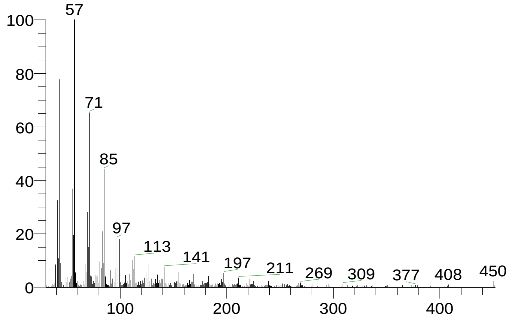

DOTRIACONTANE
Formula C32H66, MW 450, CAS# 544-85-4, Entry# 274478
AI3-52367

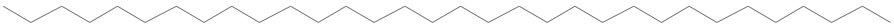

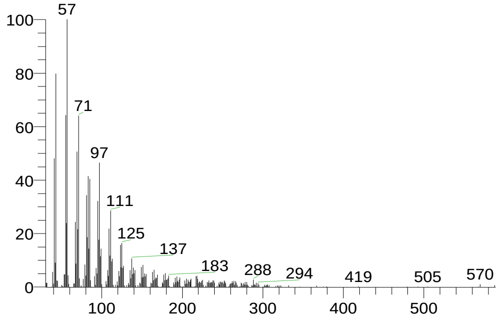

14-á-H-PREGNA
Formula C21H36, MW 288, CAS# NA, Entry# 178939
14-á-PREGNA

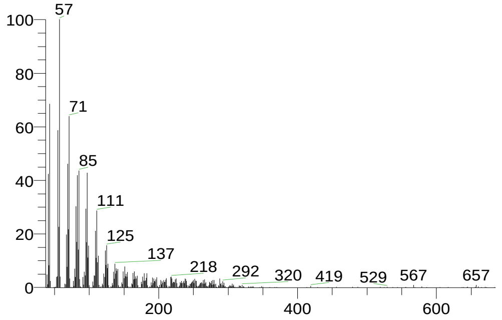

14-á-H-PREGNA
Formula C21H36, MW 288, CAS# NA, Entry# 178938
14-á-PREGNA

SI 747, RSI 768, WileyRegistry8e, Entry# 225807, CAS# NA, ISOCHIAPIN B

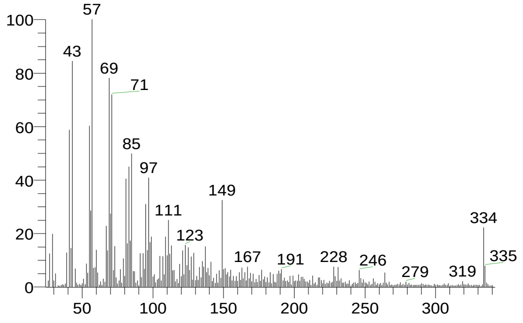

ISOCHIAPIN B
Formula C19H22O6, MW 346, CAS# NA, Entry# 225807

RT	Compound Name	Molecular Weight	Molecular Formula	Cas #	Area %	Library
29.95	DOTRIACONTANE	450	C32H66	544-85-4	1.57	WileyRegistry8e
29.95	14-á-H-PREGNA	288	C21H36	NA	1.57	WileyRegistry8e
29.95	14-á-H-PREGNA	288	C21H36	NA	1.57	WileyRegistry8e
29.95	ISOCHIAPIN B	346	C19H22O6	NA	1.57	WileyRegistry8e

My GC-MS Report

Hit Spectrum | Compound Structure

DOTRIACONTANE
Formula C32H66, MW 450, CAS# 544-85-4, Entry# 274478
AI3-52367

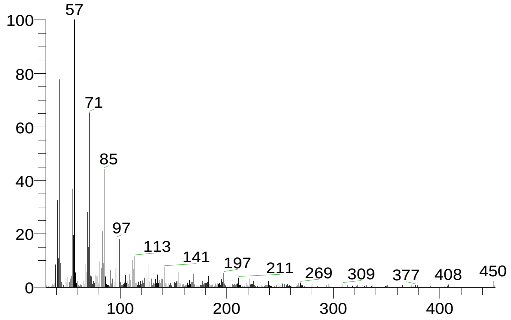

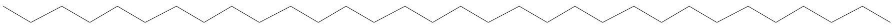

14-á-H-PREGNA
Formula C21H36, MW 288, CAS# NA, Entry# 178939
14-á-PREGNA

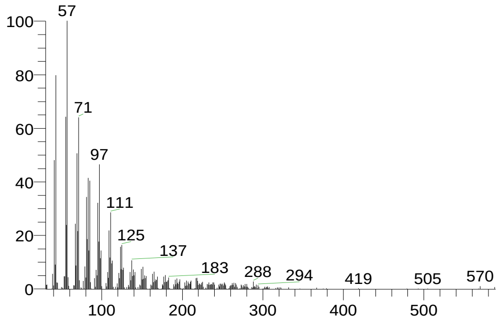

14-á-H-PREGNA
Formula C21H36, MW 288, CAS# NA, Entry# 178938
14-á-PREGNA

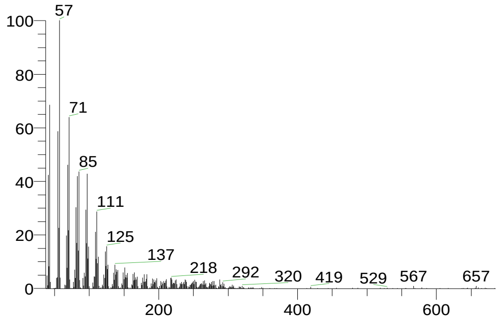

SI 732, RSI 750, WileyRegistry8e, Entry# 225807, CAS# NA, ISOCHIAPIN B

ISOCHIAPIN B
Formula C19H22O6, MW 346, CAS# NA, Entry# 225807

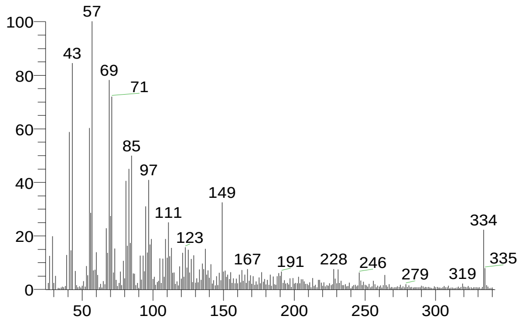

RT	Compound Name	Molecular Weight	Molecular Formula	Cas #	Area %	Library
30.21	DOTRIACONTANE	450	C32H66	544-85-4	3.74	WileyRegistry8e
30.21	14-á-H-PREGNA	288	C21H36	NA	3.74	WileyRegistry8e
30.21	14-á-H-PREGNA	288	C21H36	NA	3.74	WileyRegistry8e
30.21	ISOCHIAPIN B	346	C19H22O6	NA	3.74	WileyRegistry8e

My GC-MS Report

Hit Spectrum | Compound Structure

DOTRIACONTANE
Formula C32H66, MW 450, CAS# 544-85-4, Entry# 274478
AI3-52367

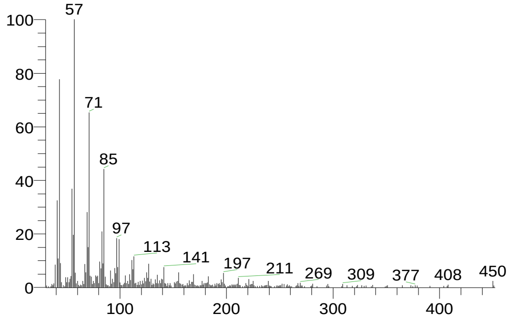

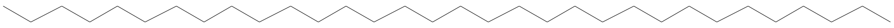

14-á-H-PREGNA
Formula C21H36, MW 288, CAS# NA, Entry# 178939
14-á-PREGNA

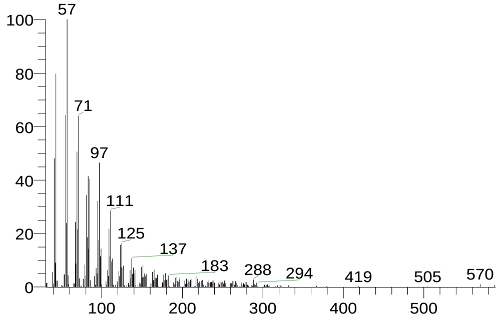

14-á-H-PREGNA
Formula C21H36, MW 288, CAS# NA, Entry# 178938
14-á-PREGNA

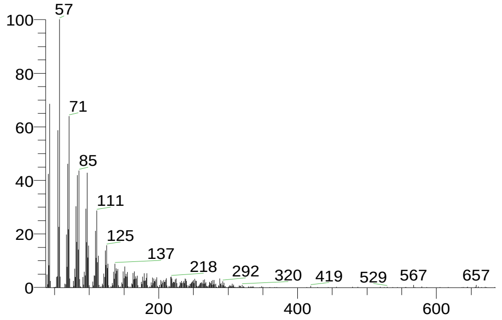

SI 732, RSI 750, WileyRegistry8e, Entry# 225807, CAS# NA, ISOCHIAPIN B

ISOCHIAPIN B
Formula C19H22O6, MW 346, CAS# NA, Entry# 225807

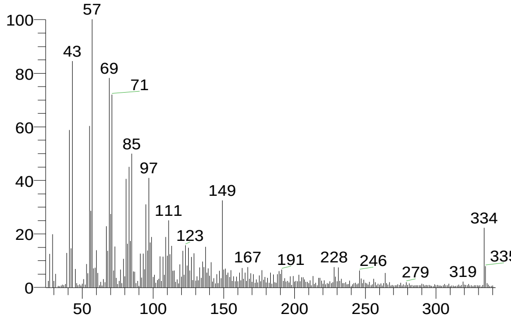

Supplement: Supplementary file 1 [file molecules-24-04280-s001.pdf]
